# Gut microbial trimethylamine *N*-oxide generation promotes risk of atrial fibrillation via muscarinic receptor–mediated autonomic dysfunction

**DOI:** 10.1172/JCI201684

**Published:** 2026-07-24

**Authors:** Selvam Arjunan, Isaiah Pemberton, Xinmin S. Li, Naseer Sangwan, Lydia Akino, Emmanuel Opoku, Dmitriy Verbovetskiy, Ina Nemet, Hyun Su Kim, Haruko Masumiya, Zeneng Wang, Joseph A. Lupica, Melissa Y. Tian, Karis Mao, Deepthi P. Mallela, Maradumane L. Mohan, Sarah M. Schumacher, Julie H. Rennison, Sathyamangla V. Naga Prasad, Kenneth R. Laurita, Vamsi Chodisetty, Mina K. Chung, David R. Van Wagoner, John Barnard, Jonathan D. Smith, Oussama Wazni, Stanley L. Hazen, Robert A. Koeth

**Affiliations:** 1 Department of Heart, Blood & Kidney Research, Cleveland Clinic Research, Cleveland Clinic, Cleveland, Ohio, USA.; 2 Department of Biomedical Engineering, Case School of Engineering School of Medicine, Case Western Reserve University, Cleveland, Ohio, USA.; 3 Heart and Vascular Research Center, MetroHealth Campus, Case Western Reserve University, Cleveland, Ohio, USA.; 4 Department of Cardiovascular Medicine, Heart, Vascular & Thoracic Institute, Cleveland Clinic, Cleveland, Ohio, USA.

**Keywords:** Cardiology, Cell biology, Clinical Research, Arrhythmias, Biomarkers, Drug therapy

## Abstract

Gut microbiota–derived trimethylamine *N*-oxide (TMAO) plays a role in the pathogenesis of cardiovascular disease, but its role in the pathogenesis of atrial fibrillation (AF) remains uncertain. TMAO levels were quantified in plasma from serial subjects undergoing elective cardiac catheterizations and shown to independently associate with prevalent AF following adjustment for risk factors. Human cAMP response element modulator isoform IbΔC-X transgenic mice (CREM-IbΔC-X) supplemented with a TMAO diet developed AF sooner. C57BL/6J mice on and off a TMAO diet had more inducible AF via a transesophageal pacing study compared with chow-fed controls. Dietary choline supplementation increased circulating TMAO levels and significantly accelerated AF onset in CREM-IbΔC-X mice. Iodomethylcholine (IMC) reduced circulating TMAO levels and choline-induced AF onset. Cecal metagenomic analyses showed that choline supplementation induced changes in microbial communities associated with AF, while many of these changes were attenuated by IMC. Choline supplementation promoted overall adverse atrial remodeling with left atrial dilation. Optical mapping studies showed that mice supplemented with choline exhibited reduced conduction velocity, shortened action potential duration at 80% repolarization, and decreased wavelength. TMAO inhibited muscarinic receptor 2, resulting in autonomic dysfunction that promotes AF. In summary, TMAO, independently associated with AF risk in subjects, enhanced AF in multiple mouse models via autonomic dysfunction and is a therapeutic target for preventing AF.

## Abstract 2

This work links a western diet, the gut microbiome, and atrial fibrillation together by causing dysfunction in our flight or flight response.

## Graphical abstract

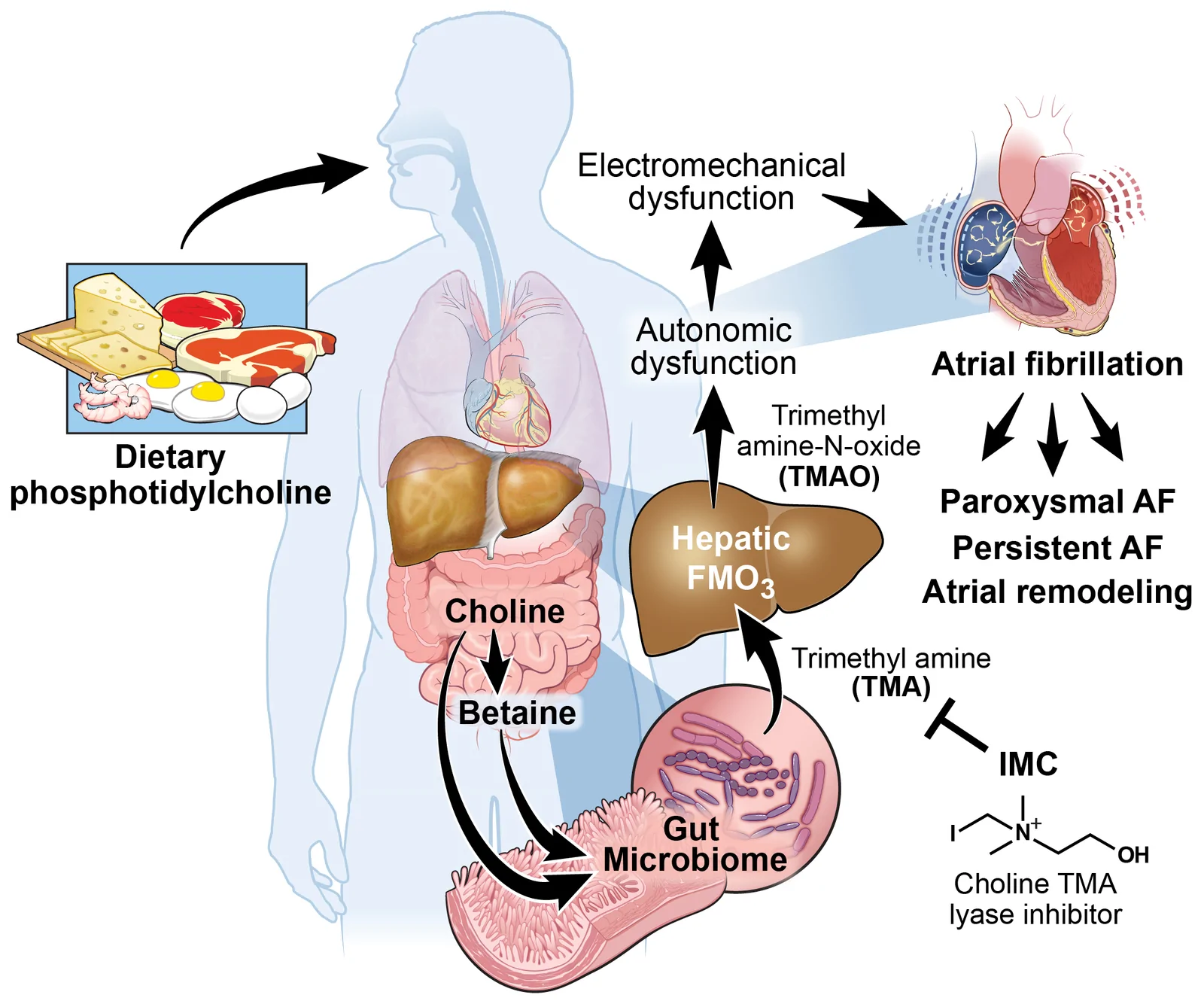

## Introduction

The trimethylamine *N*-oxide (TMAO) pathway is mechanistically linked to the pathogenesis of numerous cardiovascular diseases (CVDs) (1–3). Direct administration of TMAO, inhibition of the TMAO pathway using small-molecule inhibitors (4–8), and gut microbial transplant studies (6, 9, 10) have further supported a mechanistic link between TMAO and the gut microbiome with metabolic diseases (1, 9–11). Choline is both an abundant nutrient in Western diets and an endogenous component of bile. It is metabolized by the gut microbiota to yield trimethylamine (TMA), which is subsequently oxidized by host hepatic flavin monooxygenases (FMOs) to TMAO (12). TMAO has been linked to the initiation, progression, and sequelae of CVD in multiple animal and in vitro studies (2, 12–16). In large clinical cohort studies TMAO is independently associated with major adverse cardiovascular events and adverse heart failure outcomes (1, 2, 17–22). Mechanistically, TMAO has been implicated in the pathogenesis of CVD by increasing foam cell formation (1), disrupting reverse cholesterol transport (23), increasing platelet hyperreactivity (14), and upregulating the endoplasmic reticulum stress response by activating the protein kinase R–like endoplasmic reticulum kinase (15). TMAO has also been implicated in the pathogenesis of heart failure and kidney disease by promoting fibrosis and inflammation, pathways also important in the etiology of atrial fibrillation (AF) (8, 24–26).

Despite advances such as the advent of catheter ablation, antiarrhythmic medications, and novel oral anticoagulants, AF remains a significant cause of morbidity and mortality worldwide, and its underlying pathogenic mechanisms are not fully understood (27, 28). TMAO is involved in the pathogenesis of kidney disease and heart failure by promoting renal and cardiac fibrosis and inflammation pathways associated with an increased risk of AF (8, 16, 29). The many shared risk factors between CVD and AF suggest that there may also be a link between TMAO and AF (30). Here, we explore the relationship between TMAO and AF risk, including clinical associations as well as both direct testing of the effect of provision of TMAO and examination of the impact of pharmacological targeting of the gut microbial TMAO pathway in a mouse model of AF disease.

## Results

### Plasma TMAO concentrations independently associate with prevalent AF.

**Figure 1:**
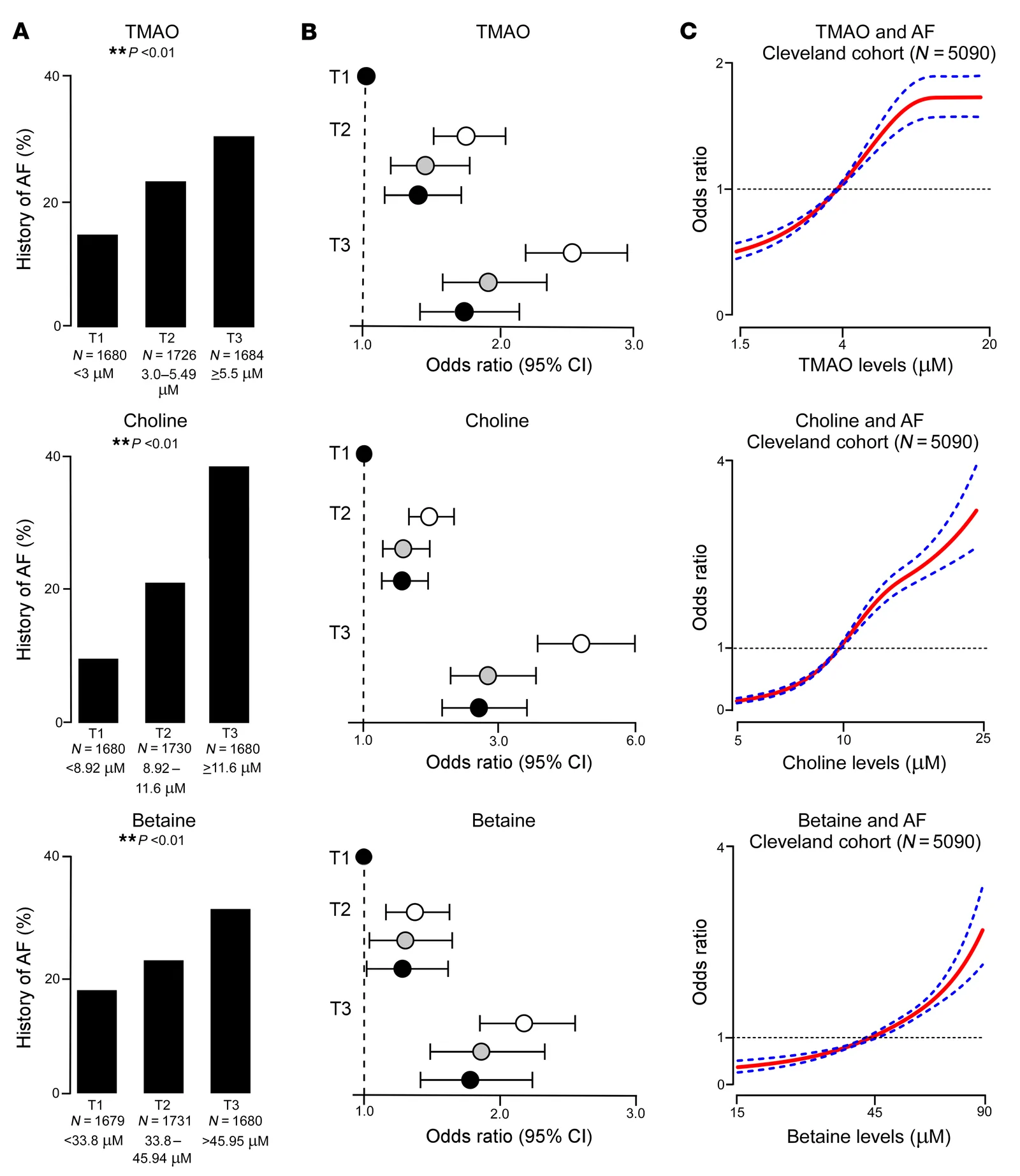
Plasma TMAO, choline, and betaine independently associate with prevalent AF. (**A**) The relationship between tertiles (T) of increasing plasma concentrations of TMAO, choline, and betaine and the proportion of prevalent AF in GeneBank subjects. The *P* value represents a Pearson’s χ^2^ test. (**B**) Forest plots of the OR of prevalent AF and tertiles of increasing concentrations of plasma TMAO, choline, and betaine with no adjustment (white circles), adjustment for patient characteristics and comorbidities (model 1: age, sex, diabetes mellitus, CVD, systolic blood pressure, current smoker, BMI, and high-sensitivity C-reactive protein; gray circles), and after full model adjustment (model 1 and eGFR; black circles). The circles represent the OR and the horizontal bars extend from the lower limit to the upper limit of the 95% CI. (**C**) Cubic spline plots of plasma concentrations of TMAO, choline, and betaine and the OR of prevalent AF. The solid red line represents the estimated OR from unadjusted logistic regression analysis at increasing values of the metabolite, and the dashed blue lines represent the 95% CI. **P < 0.01.

**Figure 2:**
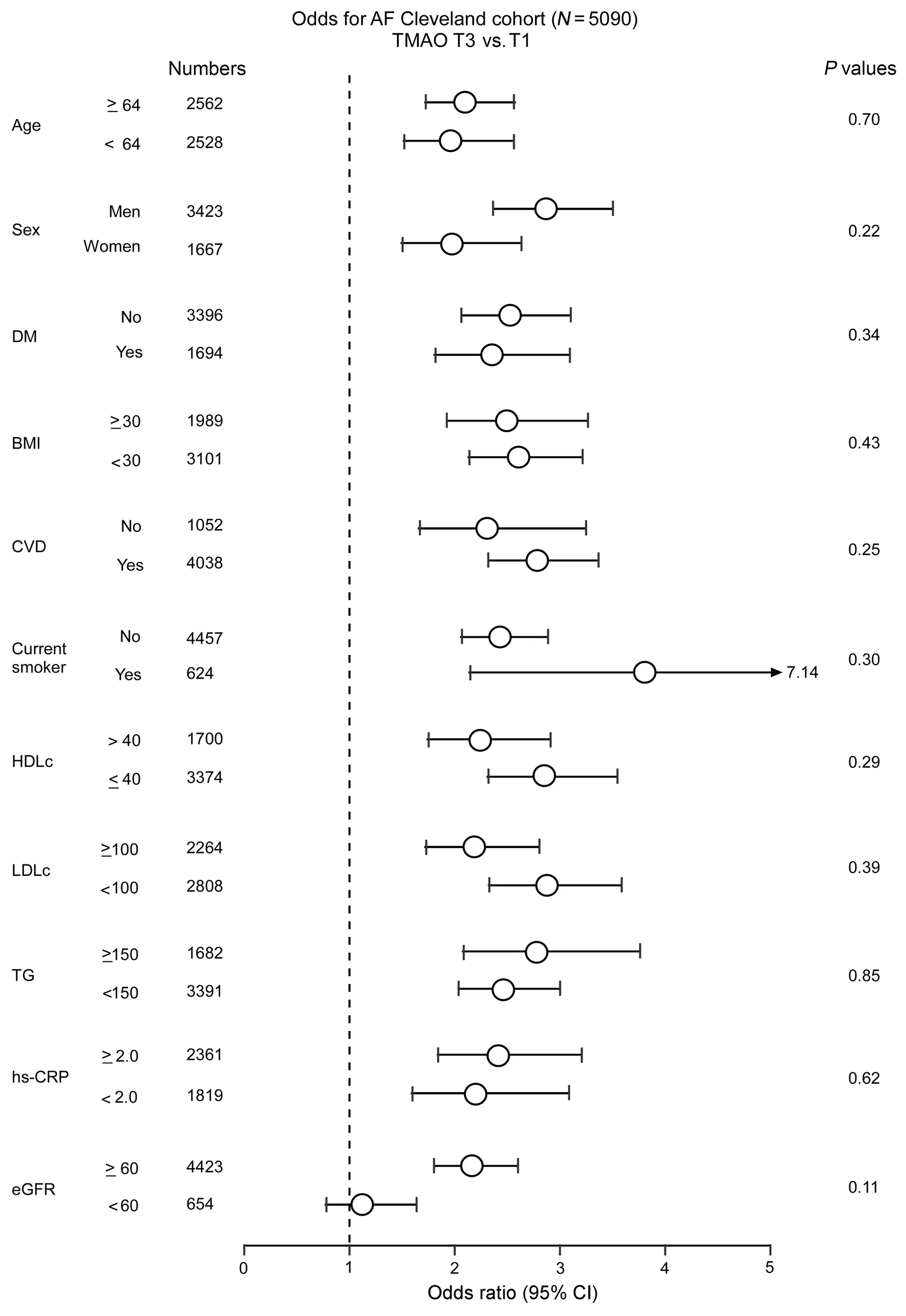
Association of baseline TMAO tertiles with odds of AF. Forest plots show OR for AF with increasing TMAO levels in the indicated subgroups. Open circles represent OR for tertile 3 (T3) versus tertile 1 (T1); horizontal lines indicate 95% CIs. *P* interaction (*P*) values were obtained from likelihood ratio tests comparing logistic models with and without the interaction between TMAO tertiles and each subgroup factor. Units for continuous variables: age (years), BMI (kg/m^2^), HDL cholesterol (HDLc; mg/dL), LDL cholesterol (LDLc; mg/dL), triglycerides (TG; mg/dL), high-sensitivity C-reactive protein (hs-CRP; mg/L), and eGFR (mL/min/1.73 m^2^). DM, diabetes mellitus.

Subjects were stratified into tertiles based on increasing plasma levels of TMAO, choline, and betaine. A significant association was observed between tertiles and history of AF for all 3 metabolites (Figure 1A). Tertiles of TMAO, choline, and betaine were also significantly associated with AF risk in an unadjusted model (Figure 1B, open circles), after adjustment for subject characteristics, comorbidities, and laboratory values (Figure 1B, gray circles, model 1) or after adjustment for subject characteristics, comorbidities, laboratory values, and kidney function (Figure 1B, black circles, model 1 + estimated glomerular filtration rate [eGFR]). Cubic spline plots of the unadjusted ORs demonstrated that low levels of plasma TMAs (TMAO, choline, and betaine) are associated with a lower likelihood of having AF and that TMAs at higher plasma concentrations are associated with a higher likelihood of having AF (Figure 1C). Forest plots of the ORs of TMAO tertiles and prevalent AF are shown for individual patient characteristics and laboratory values, and similar trends were observed across all key subgroups, with no statistically significant differences noted (Figure 2).

### Dietary TMAO supplementation accelerates AF onset and persistence in CREM-IbΔC-X transgenic mice.

**Figure 3:**
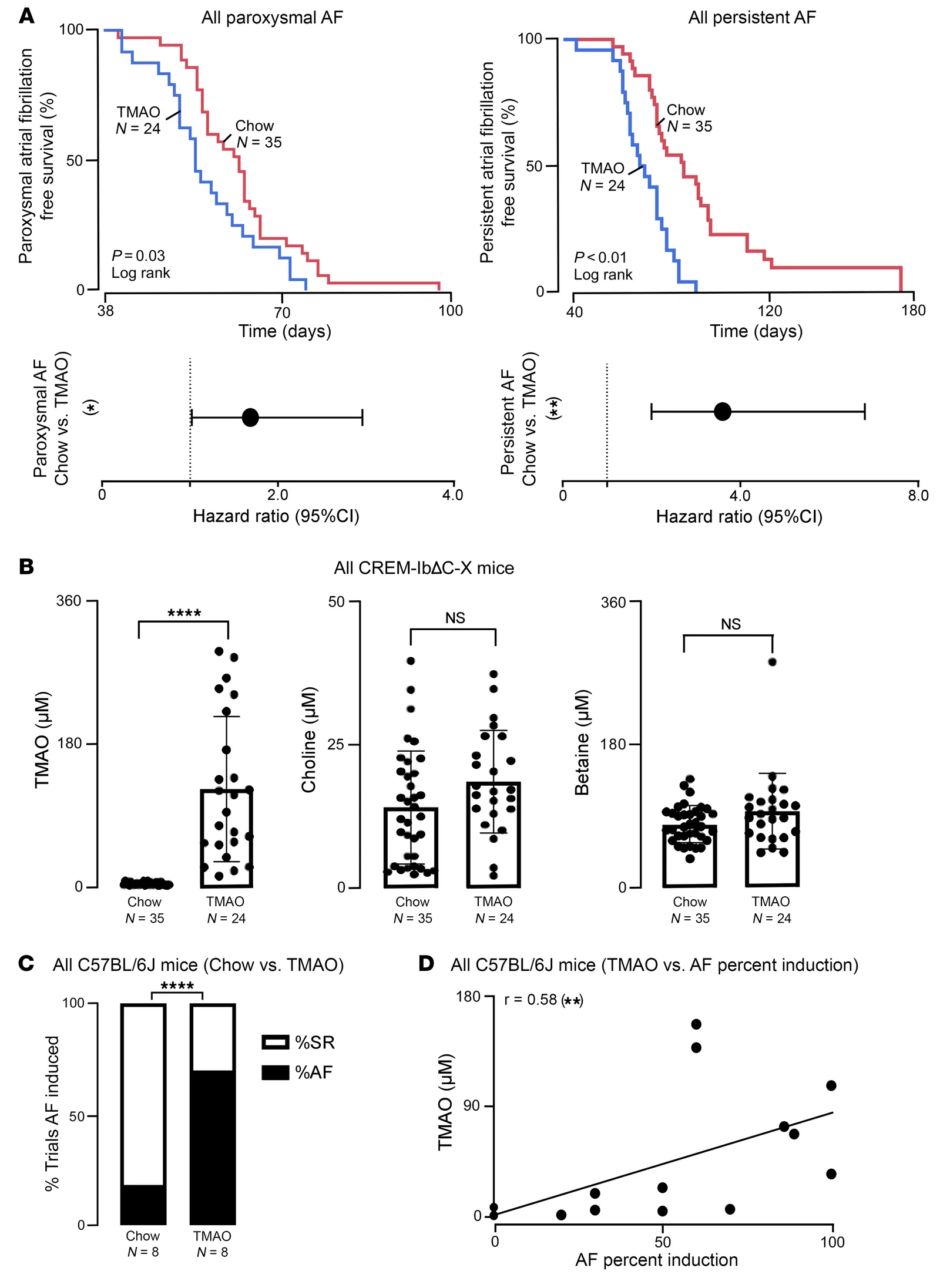
Kaplan-Meier plots and HRs of the incidence of AF in CREM-IbΔC-X mice on TMAO diet and plasma TMA concentrations. (**A**) The incidence of paroxysmal and persistent AF in all CREM-IbΔC-X mice on a chow or TMAO-supplemented diet, as represented by Kaplan-Meier plots and HRs. The circles represent the HR, and the horizontal bars extend from the lower limit to the upper limit of the 95% CI of the estimate of the HR. HRs between respective dietary groups were calculated using a Cox proportional hazard model. (**B**) Plasma concentrations of TMAs in all CREM-IbΔC-X mice used in the TMAO mouse ECG study. A Mann-Whitney test was used to assess significant differences between groups. Data represent mean ± SD. (**C**) Trials of AF percent induction in C57BL/6J mice treated with TMAO and chow diets. (**D**) TMAO levels and AF percent induction correlation in all C57BL/6J mice. Pearson’s correlation coefficient measuring the relationship between TMAO levels and AF percentage of AF. *P* values were determined using a linear regression model. *P < 0.05, **P < 0.01, ****P < 0.0001.

The finding that TMAO is independently associated with AF in humans suggested that it may contribute to AF pathogenesis and that inhibition of the gut microbial TMAO pathway could represent a potential preventive therapeutic approach. The CREM-IbΔC-X mouse is a model that develops spontaneous AF; it recapitulates human AF by first developing paroxysmal AF and then ultimately developing persistent AF (31, 32). Male and female CREM-IbΔC-X mice supplemented with dietary TMAO (0.3%) developed paroxysmal AF (first time onset of AF) and persistent AF (10 serial ECGs demonstrating AF) sooner compared with those fed a chow diet using serial needle electrode ECG measurements (Figure 3A and Supplemental Tables 1 and 2; supplemental material available online with this article; https://doi.org/10.1172/JCI201684DS1). CREM-IbΔC-X mice supplemented with dietary TMAO had significantly higher plasma levels of TMAO (Figure 3B). Similar trends were observed for the onset of paroxysmal and persistent AF and plasma TMA (TMA-containing compounds TMAO, choline, and betaine) levels in both male and female CREM-IbΔC-X mice, but female CREM-IbΔC-X mice failed to reach statistical significance for the onset of paroxysmal AF (first time AF) (Supplemental Figures 1 and 2 and Supplemental Tables 3 and 4). Notably, male and female CREM-IbΔC-X mice fed chow and TMAO-supplemented diets did not differ significantly in body weight (Supplemental Figure 3). These data suggest TMAO and, by extension, the gut microbiome has a direct role in the pathogenesis of AF.

### Dietary TMAO supplementation mediated an increase in AF susceptibility in C57BL/6J mice.

To demonstrate that the effects of TMAO were not strain dependent, we validated the key findings of our study using an alternative mouse model of AF. We performed a transesophageal AF induction study in male and female C57BL/6J mice on and off a TMAO diet. AF inducibility was increased in C57BL/6J mice fed a TMAO-supplemented diet compared with chow-fed controls (Figure 3C). Notably, TMAO supplementation resulted in an 11-fold increase in AF inducibility relative to controls (Figure 3C). When stratified by sex, both male and female C57BL/6J mice demonstrated similarly significant increases in AF inducibility (Supplemental Figure 4).

Plasma TMAO concentrations in C57BL/6J mice used in transesophageal pacing studies were also significantly increased in all C57BL/6J mice on a TMAO-supplemented diet compared with chow-fed controls (Supplemental Figure 5). Additionally, plasma levels of TMAO were significantly correlated with AF percent induction, and similar trends were observed when stratified by sex, but the correlation remained significant only in C57BL/6J male mice (Figure 3D and Supplemental Figure 6A). No consistent significant relationship was noted between AF percent induction and other plasma TMAs (Supplemental Figure 6, B and C).

### Inhibition of gut microbial TMAO production with the TMA lyase inhibitor iodomethylcholine significantly delays the onset of paroxysmal and persistent AF in CREM-IbΔC-X transgenic mice.

**Figure 4:**
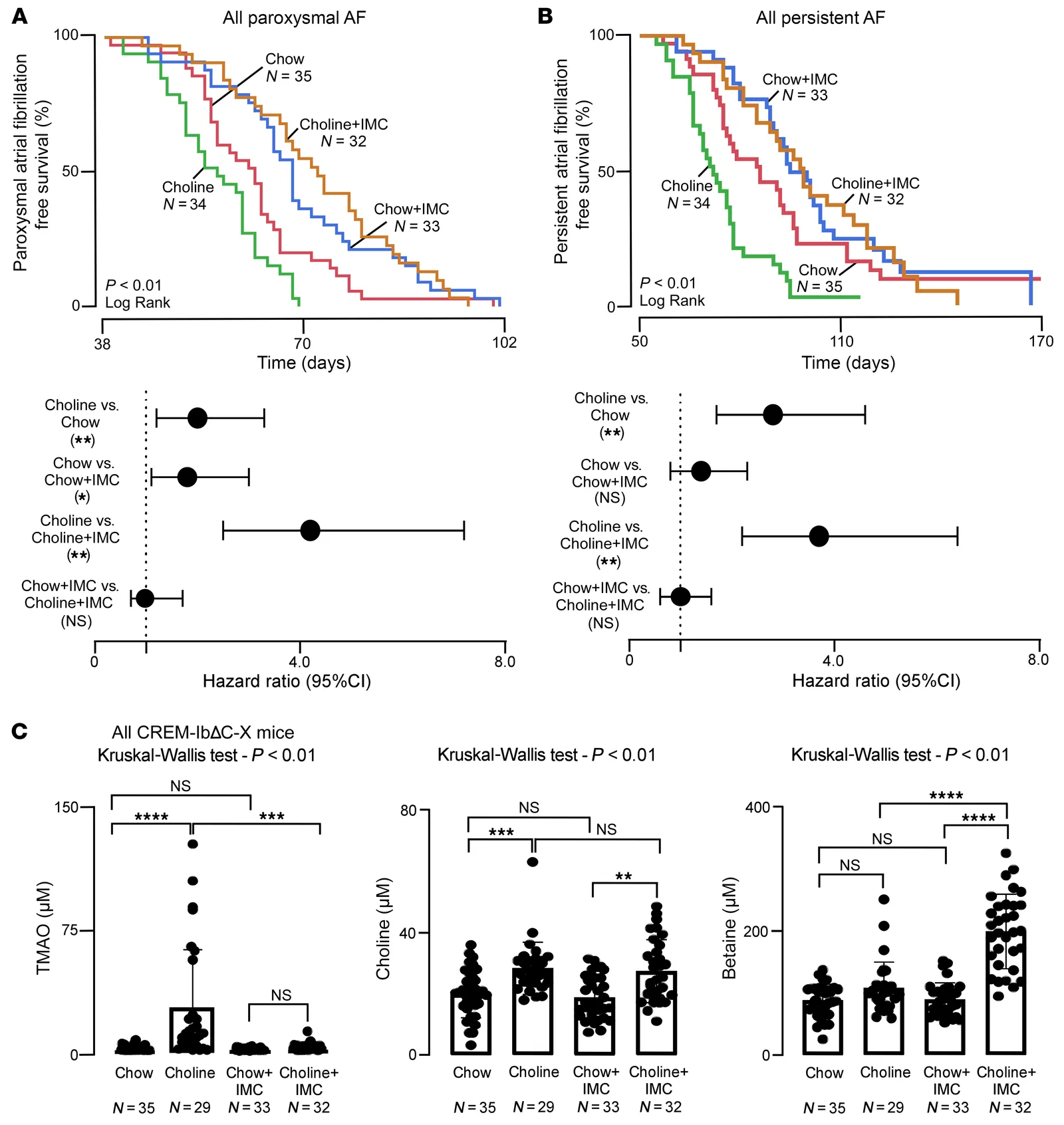
Kaplan-Meier plots and HRs of the incidence of paroxysmal AF and persistent AF in CREM-IbΔC-X mice on respective diets. (**A** and **B**) The incidence of paroxysmal (**A**) and persistent AF (**B**) in all CREM-IbΔC-X mice, as represented by a Kaplan-Meier plots and HRs, on a chow diet, a choline-supplemented diet, a chow + IMC-supplemented diet, and a choline + IMC-supplemented diet. The circles represent the HR, and the horizontal bars extend from the lower limit to the upper limit of the 95% CI of the estimate of the HR. HRs between respective dietary groups were calculated using a Cox proportional hazard model. (**C**) Average plasma concentrations of TMAO, choline, and betaine in all CREM-IbΔC-X mice used in the ECG study. Data represent mean ± SD. A Kruskal-Wallis test with Dunn’s post hoc analysis was used to determine the significant differences among the medians of 4 independent diet groups, and multiple paired comparison adjustments were performed. *P < 0.05, **P < 0.01, ***P < 0.001, ****P < 0.0001.

We next sought to determine whether targeting the gut microbial TMAO pathway could provide a potential therapeutic strategy for AF. CREM-IbΔC-X mice fed a choline-supplemented diet developed paroxysmal AF and persistent AF earlier (Figure 4, A and B, and Supplemental Tables 5 and 6) than chow-fed controls. Administration of iodomethylcholine (IMC), an inhibitor of microbial choline to TMA pathway (7), in animals maintained on a high-choline diet (choline + IMC) resulted in marked suppression of choline diet–induced AF (Figure 4, A and B, and Supplemental Tables 5 and 6). Comparable trends in the onset of paroxysmal and persistent AF were observed in each sex, but CREM-IbΔC-X female mice on choline-supplemented diet did not show a significant increase in paroxysmal AF (first time AF) compared with chow-fed controls (Supplemental Tables 7 and 8). Notably, in chow-fed mice (choline content 0.8%–0.9%) (1), IMC supplementation significantly delayed the development of paroxysmal AF compared with controls and showed a trend toward delayed onset of persistent AF (Figure 4, A and B, and Supplemental Tables 5 and 6).

CREM-IbΔC-X mice fed a choline-supplemented diet had significantly higher plasma concentrations of TMAO (Figure 4C) and TMA (Supplemental Figure 9) compared with the chow-fed (control) mice. Dietary supplementation of choline in combination with IMC led to marked reduction in plasma concentration TMAO and TMA (Figure 4C and Supplemental Figure 9). TMAO and TMA levels were not significantly reduced in mice fed a chow (control) diet in combination with IMC (Figure 4C and Supplemental Figure 9).

Consistent with the well-established observation that FMO3 (the liver enzyme responsible for the oxidation of TMA to TMAO) is expressed at higher levels in female than male mice (9), CREM-IbΔC-X female mice had higher circulation TMAO than male CREM-IbΔC-X mice (Supplemental Figure 10). This observation was also true of CREM-IbΔC-X female and male mice on a chow diet (CREM-IbΔC-X female chow TMAO, 3.1 ± 1.5 μM; CREM-IbΔC-X male chow TMAO, 0.5 ± 0.24 μM). Choline plasma concentrations were higher in the choline diet compared with the chow diet in both male and female CREM-IbΔC-X mice (Figure 4C). Betaine plasma concentrations tended to be highest in supplemented mice supplemented with choline + IMC compared with the other diets (Figure 4C and Supplemental Figure 10).

**Figure 5:**
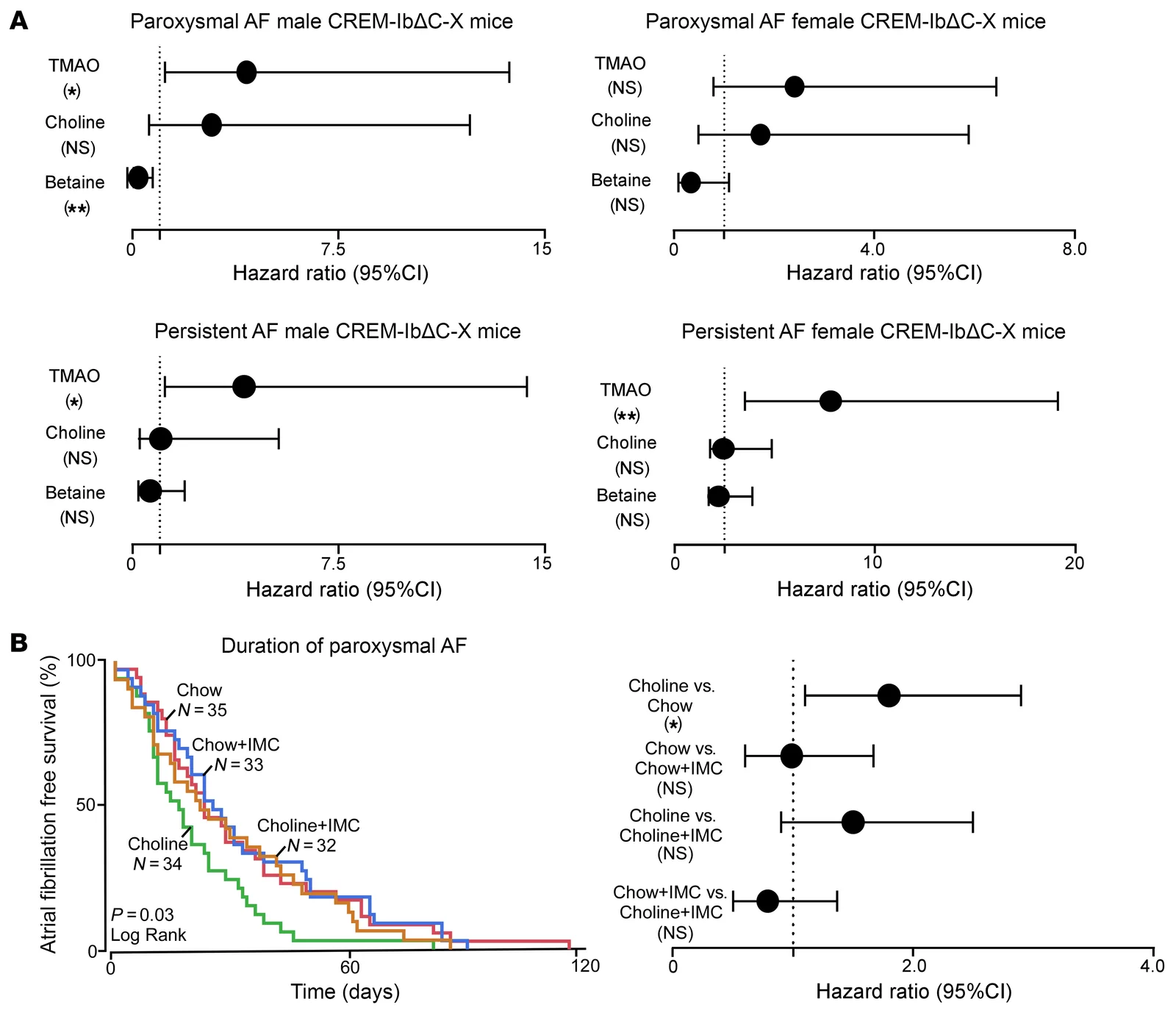
HRs and Kaplan-Meier plot of the incidence of paroxysmal and persistent AF. (**A**) The incidence of paroxysmal and persistent AF and average plasma TMAO, choline, and betaine concentrations in male and female CREM-IbΔC-X mice. (**B**) Kaplan-Meier plot and HRs of the duration of paroxysmal AF in male and female CREM-IbΔC-X mice. In **A** and **B**, the circles represent the HR, and the horizontal bars extend from the lower limit to the upper limit of the 95% CI of the estimate of the HR. HRs between respective dietary groups were calculated using a Cox proportional hazard model. Each HR represents the risk over the entire analyte concentration range. *P < 0.05, **P < 0.01.

Plasma TMAO concentrations significantly predicted the earlier onset of paroxysmal AF in all male CREM-IbΔC-X mice and approached significance in female CREM-IbΔC-X mice (Figure 5A). Higher plasma betaine concentrations associated with later onset of paroxysmal AF in male CREM-IbΔC-X mice, whereas plasma choline levels had no association with the onset of paroxysmal AF (Figure 5A). Higher TMAO levels predicted the earlier onset of persistent AF in both male and female CREM-IbΔC-X mice, whereas plasma betaine and choline plasma concentrations did not (Figure 5A).

In general, CREM-IbΔC-X female mice developed AF earlier and progressed to persistent AF more rapidly than male CREM-IbΔC-X mice (Supplemental Figures 7 and 8). In contrast, serial needle electrode ECGs performed in WT mice fed either chow or a choline-supplemented diet revealed no onset of AF over the study period (Supplemental Figure 11). Notably, CREM-IbΔC-X mice in the ECG studies showed no significant differences in body weight at the time of euthanasia, and no appreciable liver pathology was observed (Supplemental Figures 12 and 13). To determine whether the earlier development of persistent AF in CREM-IbΔC-X mice was simply due to earlier AF onset, we examined the duration of paroxysmal AF (the time between the onset of AF and persistent AF). This duration was significantly shorter in choline-supplemented CREM-IbΔC-X mice compared with chow-fed or IMC-treated mice (Figure 5B and Supplemental Table 9). Collectively, these data suggested that TMAO contributes to both the onset and progression of AF and that inhibition of the gut microbial TMAO pathway may represent a potential strategy to prevent AF development and progression.

### Pharmacological targeting of gut microbial CutC/D resides in the gut and is not readily absorbed.

**Figure 6:**
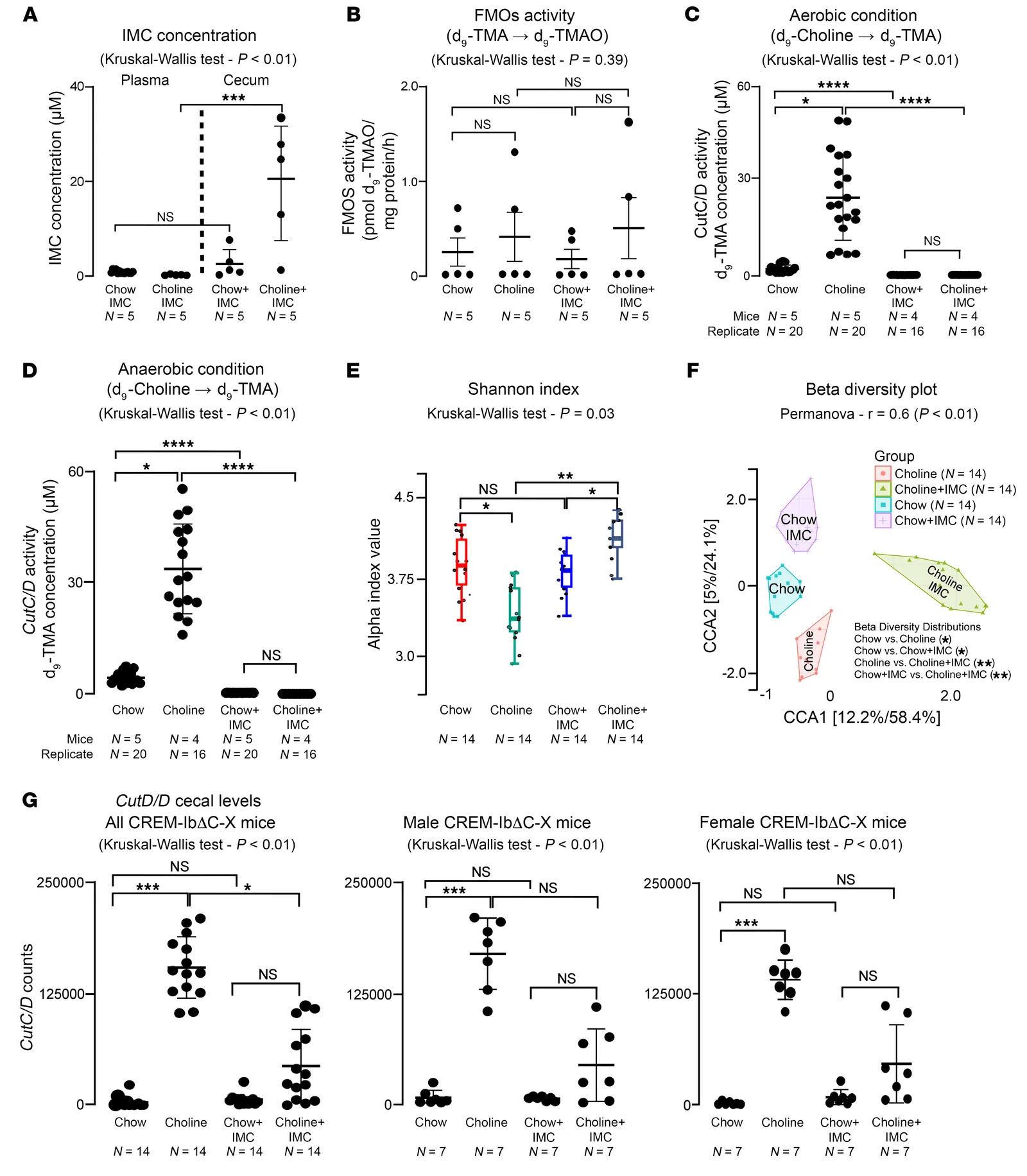
IMC concentrations in plasma and cecum samples and assessment of *CutC/D* activity. (**A**) Quantification of IMC in cecum and plasma samples of CREM-IbΔC-X mice on respective diets. (**B**) Quantification of the conversion of d_9_-TMA to d_9_-TMAO in liver samples of mice on respective diets. (**C** and **D**) Quantification of the conversion of d_9_-choline to d_9_-TMA in cecum of CREM-IbΔC-X mice under aerobic (**C**) and anaerobic (**D**) conditions on respective diets. Replicates represent 4 independent samples from each mouse. (**E** and **F**) α-Diversity plot (**E**; Shannon’s index) and principal component analysis (**F**) of β-diversity plot of CREM-IbΔC-X mice on respective diets. Permutational ANOVA (Permanova) was used to compare the β-diversity distributions of dietary groups. (**G**) *CutC/D* mRNA counts in cecum samples from all, male, and female CREM-IbΔC-X mice utilized in the mouse ECG study. In **A**–**E** and **G**, data represent mean ± SD. A Kruskal-Wallis test with Dunn’s post hoc analysis was used to determine the significant difference among the medians of 4 independent diet groups, and multiple paired comparison adjustments were performed. CCA, canonical correspondence analysis. *P < 0.05, **P < 0.01, ***P < 0.001, ****P < 0.0001.

To evaluate the potential of IMC as pharmacological therapy, we first assessed its absorption in mice receiving long-term IMC supplementation. Notably, the majority of IMC remained in the cecal contents of CREM-IbΔC-X mice, with minimal levels detected in plasma (Figure 6A). In addition, IMC treatment had no significant impact on hepatic FMO activity, as monitored by direct examination of the metabolic transformation activity of TMA into TMAO in liver homogenates (Figure 6B). In contrast, mice chronically treated with IMC showed potent inhibition in cecal microbial enzymatic transformation capacity of d_9_-choline into d_9_-TMA under both aerobic and anaerobic conditions (Figure 6, C and D). We observed that plasma TMAO concentrations in CREM-IbΔC-X mice fed a choline-supplemented diet and IMC were persistently suppressed for the duration of the ECG study (~5 months) (Supplemental Figure 14). In contrast, CREM-IbΔC-X mice fed a choline diet alone exhibited persistently high plasma TMAO levels over the same period (Supplemental Figure 14). The persistent suppression or production of TMAO over time suggested that there were stable microbiota alterations present. These data also suggest that IMC effectively and chronically inhibits the TMA/TMAO gut microbial pathway, is not the result of off-target effects on liver FMO activity, and could be considered a long-term preventive therapeutic pharmacological agent.

### Choline-supplemented diet altered gut microbiota composition in CREM-IbΔC-X transgenic mice, and treatment with IMC reversed these alterations.

We next characterized the gut microbiota using metagenomic sequence analysis of cecum in a subset of male and female CREM-IbΔC-X mice used in the mouse ECG study. CREM-IbΔC-X mice on the choline-supplemented diet in the ECG study showed a significant decrease in gut microbiome diversity, which was reversed by IMC treatment (Figure 6E). A canonical correspondence analysis plot demonstrated that mice on a choline diet showed differences in diversity with microbial taxa compared with respective control groups (Figure 6F). Metagenomic associations are provided in Supplemental Tables 10 and 11. Compositional differences of the gut microbiota showed that the relative abundance of the phylum Firmicutes was higher in CREM-IbΔC-X mice supplemented with a choline diet compared with chow and choline + IMC controls (Supplemental Figure 15A, species highlighted in yellow). At the species level, the relative abundance of GGB29005_SGB41723 was greater in the choline-supplemented diet compared with the chow and choline + IMC dietary control groups (Supplemental Figure 15, A and B). The species GGB18827_SGB41440 of the phylum Firmicutes was less abundant in the choline group compared with the respective dietary control groups (Supplemental Figure 15, A and B).

**Figure 7:**
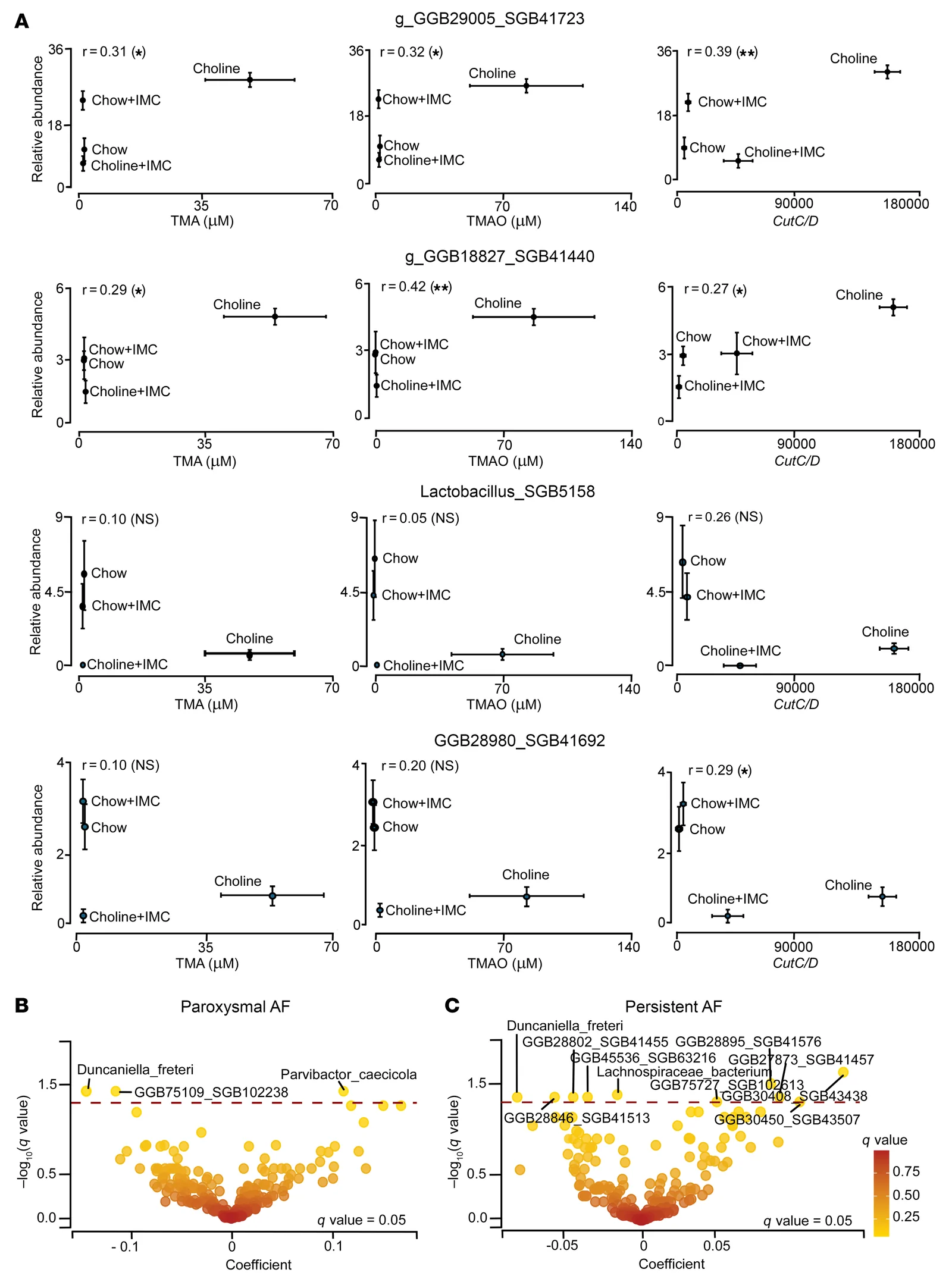
*CutC* levels and correlation with gut microbiome and differential abundance of gut microbial species with the time of onset of paroxysmal and persistent AF. (**A**) Gut microbiota taxa abundance and its association with mouse plasma TMA and TMAO and *CutC/D* mRNA counts in linked cecal samples of male and female CREM-IbΔC-X mice on respective diets. The circles represent the mean, and the bars represent SEM with regression and *P* values determined using the linear regression model. *P < 0.05, **P < 0.01. (**B** and **C**) Volcano plot showing the association of differential abundance of gut microbial species with the time of onset of paroxysmal (**B**) and persistent (**C**) AF. In the plot, the *x* axis shows the coefficient (effect size and direction), and the *y* axis shows the –log_10_(*q* value). Each circle represents a specific microbial species, colored on a yellow-to-red gradient indicating *q* values, with yellow being more significant. The dashed dark red line at *y* ≈ 1.30 marks the statistical significance threshold (*q* value = 0.05). Circles above this line and labeled are statistically significant (*q* < 0.05). Circles in the upper corners of the plot, far from 0 on the *x* axis and high on the *y* axis, represent features with both large effect sizes and high statistical significance. Benjamini-Hochberg false discovery rate correction was used to adjust *q* values for multiple testing.

*CutC/D* (catalytic subunit of choline TMA lyase) mRNA counts were significantly higher in the choline diet compared with the chow and choline + IMC groups (Figure 6G). The same trend was observed in CREM-IbΔC-X mice after stratification by sex (Figure 6G). We next investigated the association of genera that positively and inversely associated with the choline diet with the TMA, TMAO, and *CutC/D* levels in CREM-IbΔC-X mice. A strong and significant positive correlation between the relative abundance of the gut microbial species GGB29005_SGB41723 and GGB18827_SGB41440 was observed for TMA, TMAO, and *CutC/D* levels in CREM-IbΔC-X mice (Figure 7A). Although no significant inverse association was found between gut microbial species Lactobacillus_SGB5158 and GGB28980_SGB41692 with TMA and TMAO concentration, *CutC/D* levels showed a significant inverse relationship (Figure 7A). Other gut microbial species noted to have high or low relative abundances with CREM-IbΔC-X mice supplemented with a choline diet did not show a significant association with TMA, TMAO, or *CutC/D* (Supplemental Figure 16).

### Gut microbial taxa associate with the onset of paroxysmal and persistent AF.

In the final set of gut microbial studies, we assessed the changes in cecal microbial communities and the association with the time to onset of paroxysmal AF and persistent AF in CREM-IbΔC-X mice. Volcano plots (Figure 7, B and C) showed that several significant gut microbial species were associated with the time of onset of paroxysmal AF and persistent AF. In contrast, the duration of AF did not show any significant association with gut microbial species (Supplemental Figure 17). *Parvibacter caecicola* was the only species significantly associated with the time to paroxysmal AF. GGB30408_SGB43438 was significantly associated with the time to persistent AF. None of the gut microbial bacterial species had overlap with species that significantly associated with a choline diet.

### A TMAO-promoting diet was associated with atrial remodeling in CREM-IbΔC-X and C57BL/6J mice.

**Figure 8:**
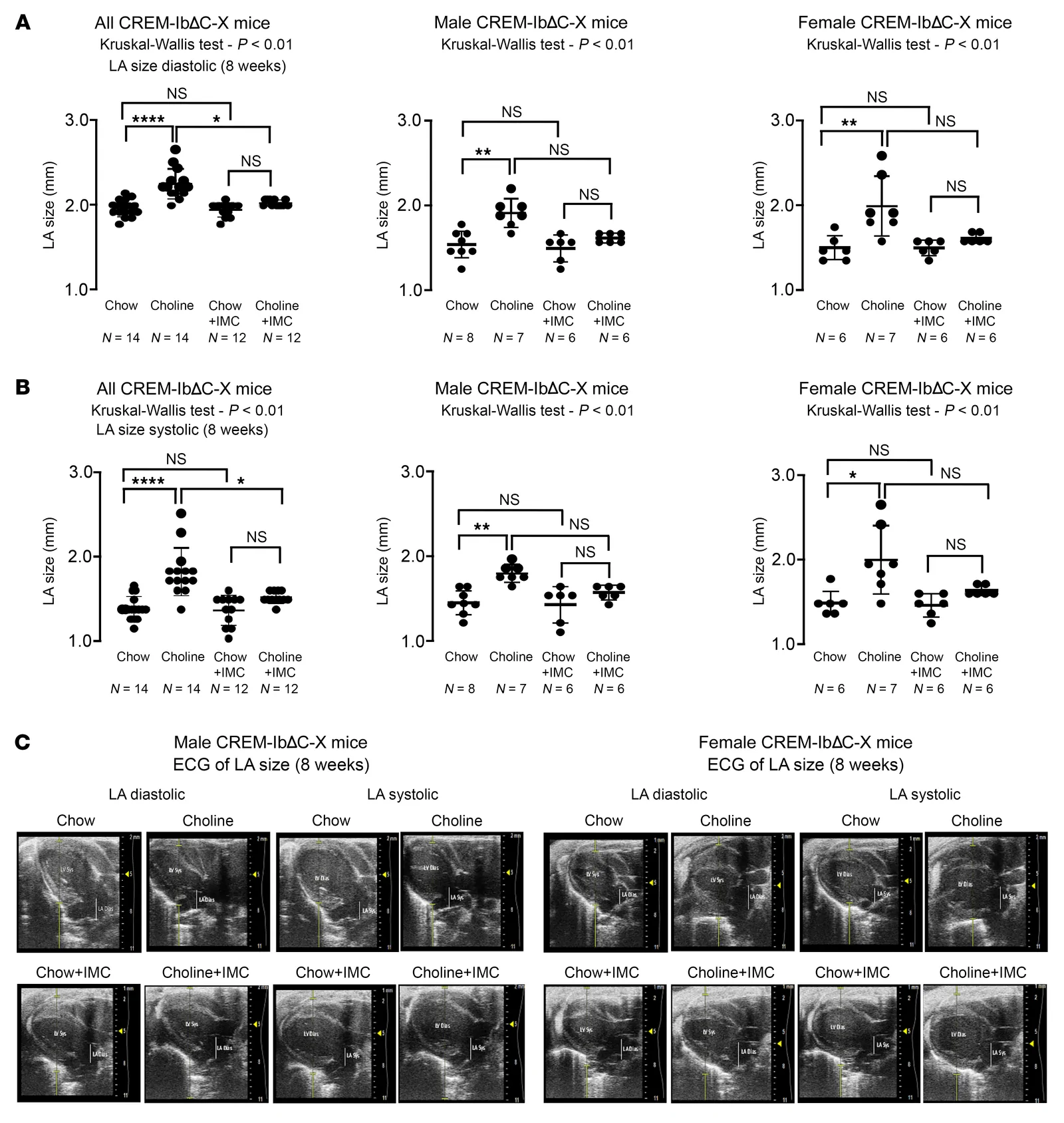
Choline diet increases the LA size during diastolic and systolic function. (**A**) LA diastolic size in male and female CREM-IbΔC-X mice at 8 weeks of age on respective diets. (**B**) LA systolic size in male and female CREM-IbΔC-X mice at 8 weeks of age on respective diets. (**C**) Echocardiography of LA size in female and male CREM-IbΔC-X mice at 8 weeks of age on respective diets. In **A** and **B**, data represent mean ± SD. A Kruskal-Wallis test with Dunn’s post hoc analysis was used to determine the significant difference among the medians of 4 independent diet groups, and multiple paired comparison adjustments were performed. *P < 0.05, **P < 0.01, ****P < 0.00001.

We next investigated whether dietary choline supplementation, with and without IMC, in CREM-IbΔC-X mice was associated with structural and functional changes in the atria and ventricles. Left atria (LA) size, measured in both diastole and systole, was significantly increased in male and female CREM-IbΔC-X mice fed a choline-supplemented diet compared with chow-fed controls (*P* < 0.01) at both 5 weeks (Supplemental Figure 18) and 8 weeks of age (Figure 8, A–C). Notably, the LA size in both systole and diastole was significantly attenuated in the choline + IMC dietary group at 8 weeks of age (Figure 8, A–C), suggesting that pharmacological treatment with IMC can prevent TMAO-associated structural changes. In contrast, no significant differences were observed in left ventricular (LV) mass, LV ejection fraction, cardiac output, fractional shortening, or heart rate among dietary groups, as measured by echocardiography at 5 and 8 weeks of age (Supplemental Figures 19–22). Since inflammation may play a role in atrial remodeling and AF, we also evaluated circulating inflammatory markers. Serum amyloid A and IL-6 were not significantly different between choline-supplemented mice and chow-fed controls (Supplemental Figure 23).

We also performed echocardiograms on C57BL/6J male and female mice used in the transesophageal AF induction studies. Similar to CREM-IbΔC-X mice, C57BL/6J mice on a TMAO-supplemented diet showed an increase in LA size that was significantly greater compared with chow-fed C57BL/6J mice in both atrial diastole (*P* < 0.01) and systole (*P* < 0.01) (Supplemental Figure 24, A and B). Similar trends were observed when stratifying mice by sex (Supplemental Figure 24A). In contrast, no significant differences were observed in LV mass, LV ejection fraction, cardiac output, fractional shortening, and heart rate, as measured by echocardiography among TMAO and chow groups in C57BL/6J mice at 6 weeks of age (Supplemental Figure 25).

### The NLRP3 inflammasome is not a significant contributor to TMAO-promoted AF.

We performed in vitro and in vivo studies examining the role of the NLRP3 (NOD-, LRR-, and pyrin domain-containing protein 3) inflammasome in the early onset of AF in choline-supplemented CREM-IbΔC-X mice. We first exposed multiple cell types to physiological relevant levels of TMAO and examined the expression of *NLRP3* and *IL-1β*. The cell types included human cardiac fibroblasts (Supplemental Figure 26, A and B), atrial engineered heart tissues (aEHTs) (Supplemental Figure 27, A and B), primary mouse atrial and ventricular cardiomyocytes, and primary mouse atrial and ventricular fibroblast treated with vehicle or TMAO (Supplemental Figures 28 and 29). We observed no significant increase in the relative mRNA expression of *NLRP3* or *IL-1β* in cells exposed to TMAO. However, a significant decrease was detected in *IL-1β* expression in primary atrial fibroblasts treated with TMAO at high concentrations (1 mM) compared with control (Supplemental Figures 29).

To investigate the role of the NLRP3 inflammasome in the early onset of AF observed in choline-supplemented CREM-IbΔC-X mice, we performed mouse ECG experiments using a potent and selective inhibitor of NLRP3 inflammasome, MCC950. CREM-IbΔC-X mice supplemented with both choline and MCC950 in water had no significant change in developing paroxysmal AF compared with CREM-IbΔC-X mice supplemented with a choline diet (Supplemental Figure 30A and Supplemental Table 12). CREM-IbΔC-X mice supplemented with both choline in combination with MCC950 developed persistent AF significantly earlier compared with CREM-IbΔC-X mice supplemented with a choline diet alone (Supplemental Figure 30B and Supplemental Table 13). We confirmed that MCC950 systemically inhibited the NLRP3 inflammasome in CREM-IbΔC-X mice by performing LPS i.p. injections and measuring plasma concentrations of IL-1β (a specific cytokine product of the NLRP3 inflammasome) and TNF-α, a nondependent NLRP3 cytokine. Compared with vehicle (PBS), there was no significant decrease in plasma concentrations of TNF-α, but there was a significant decrease in the concentration of IL-1β, consistent with MCC950 NLRP3 inhibition (Supplemental Figure 30, C and D). We also observed that the same CREM-IbΔC-X mice treated with choline in combination with MCC950 had higher TMAO levels compared with the control mice fed a chow diet (Supplemental Figure 30E). Together, these data suggest that the NLRP3 inflammasome is not a significant pathogenic mechanism in TMAO-promoted AF in CREM-IbΔC-X mice.

### A choline diet is associated with atrial electrophysiological dysfunction.

**Figure 9:**
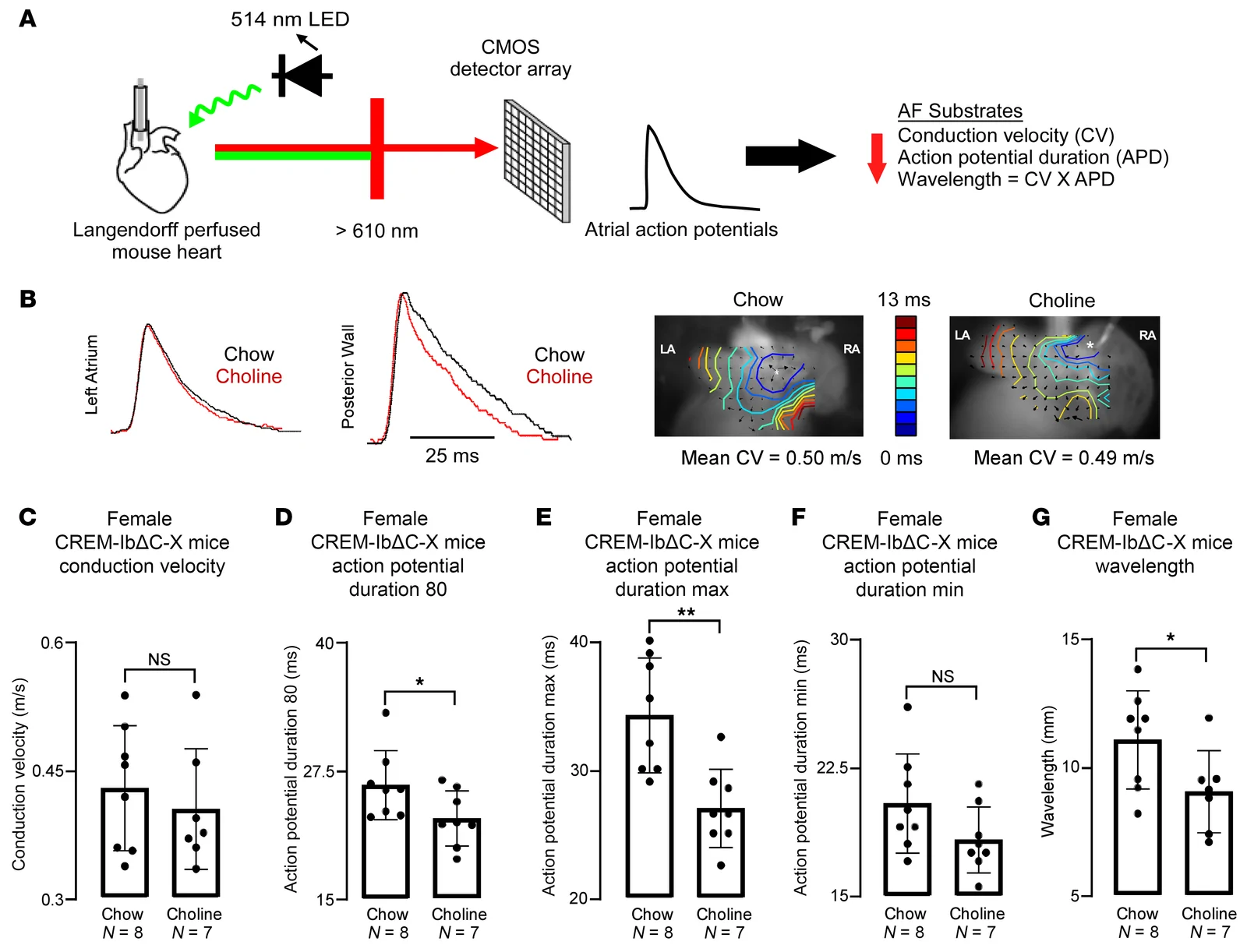
Analysis of CV and APD in CREM-IbΔC-X mouse hearts using ex vivo optical mapping on chow versus choline-supplemented diets. (**A**) Schematic of the optical mapping experimental setup used for whole-heart atrial action potential measurements (see text for details). (**B**) Representative examples of action potentials (left) measured from the LA and posterior wall for chow (black) and choline (red), and atrial activation maps (posterior view) during 130 ms pacing (asterisk) with local CV vectors superimposed for chow (middle) and choline (right) RA, right atria. (**C**–**G**) Grouped data of CV (**C**), APD80 (**D**), APD80 maximum (**E**), APD 80 minimum (**F**), and wavelength (**G**) at a pacing cycle length of 130 ms. All *P* values shown were determined using 2-tailed Student’s *t* test to compare the means of 2 independent groups, after confirmation of normality using Shapiro-Wilk and Kolmogorov-Smirnov tests. *P < 0.05, **P < 0.01.

To understand the electrophysiological implications of a choline-supplemented diet, we performed optical action potential mapping studies on female CREM-IbΔC-X mice (Figure 9A). Female CREM-IbΔC-X mice were selected due to relatively higher plasma TMAO levels, as observed in this study and previous studies (33). Representative examples of atrial action potentials, activation isochrone maps, and local conduction velocity (CV) vectors are shown in Figure 9B. Measurement of average local CV vectors demonstrated a nonsignificant decrease in CREM-IbΔC-X female mice fed a choline-supplemented diet compared with mice fed a chow diet (Figure 9C). In contrast, action potential duration at 80% repolarization (APD80) and maximum APD80 were significantly reduced in the choline group, while minimum APD80 showed a trend toward significance (Figure 9, D–F). Shortening of APD is a well-established mechanism underlying increased susceptibility to AF (34–39). Consistent with this, the calculated cardiac wavelength (CV × APD80) was also significantly shorter in mice receiving choline supplementation (Figure 9G). Notably, reduced wavelength is associated with an increased vulnerability to AF (34–39).

In contrast, we found no significant change in beat rate, APD at 50% repolarization (APD50), or calcium amplitude in aEHTs exposed to TMAO (Supplemental Figure 31). We also performed an ex vivo study of male and female CREM-IbΔC-X Langendorff perfused mouse hearts with direct TMAO exposure. Similarly, we found no significant changes in APD50, APD80, or CV in optical mapping studies performed on ex vivo male and female CREM-IbΔC-X Langendorff perfused mouse hearts with direct TMAO exposure (Supplemental Figures 32–34).

### TMAO promotes autonomic dysfunction by inhibition of muscarinic receptor 2.

**Figure 10:**
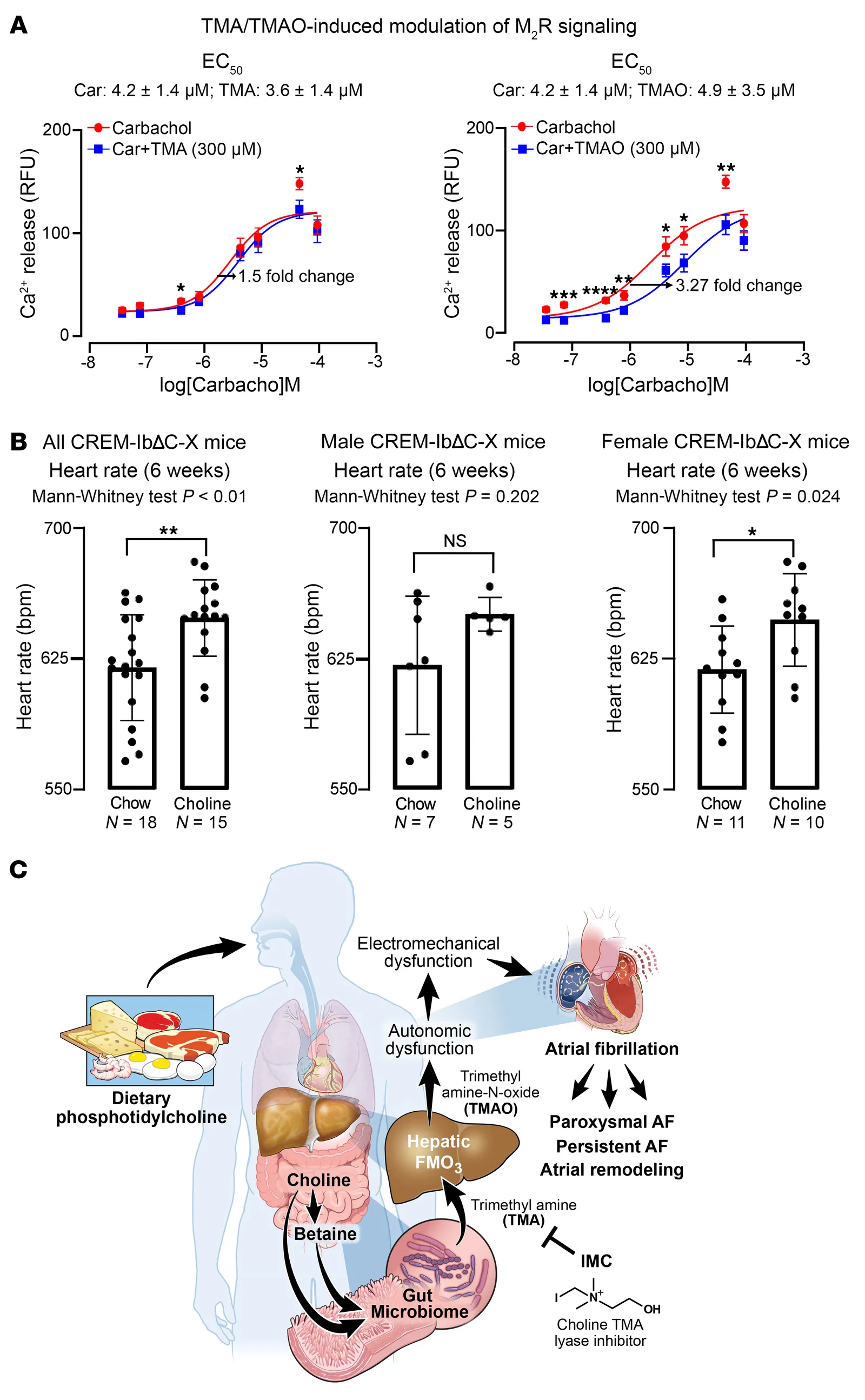
TMAO modulates autonomic signaling. (**A**) TMA/TMAO induced inhibition of M_2_R (clone 1) signaling in HEK293 cells. Unpaired 2-tailed Welch’s *t* test was used to obtain the fold change *P* value by comparing the mean EC_50_ values of carbachol (Car) and TMA/TMAO (*n* = 6 independent experiments). (**B**) Heart rate in CREM-IbΔC-X mice treated with choline and chow diet. Data represent mean ± SD. A Mann-Whitney test was used to assess significant differences between groups. *P < 0.05, **P < 0.01. (**C**) Schematic demonstrating that IMC inhibits TMA/ TMAO formation, AF induction, and progression. Choline diet alters gut microbial TMA and its metabolite formation. TMAO formation plays a key role in autonomic dysfunction via inhibition of M_2_R and the resulting increased sympathetic tone and susceptibility to AF. IMC that inhibits formation of TMA/TMAO can reduce AF induction and progression.

TMAO has structural similarities to acetylcholine. Optical mapping studies demonstrated that mice fed a choline-supplemented diet exhibited significantly shortened APD (Figure 9D). In addition, a prior report has shown that direct injection of TMAO into the ganglion plexi of the cardiac autonomic nervous system shortens the effective refractory period, a surrogate for APD duration, and increased AF inducibility in canine myocardium using a burst pacing protocol (30). Together, these data suggested that TMAO may promote AF through autonomic dysfunction, potentially via interaction with muscarinic receptors. To explore this mechanism, we generated HEK293 cells stably expressing M_2_ muscarinic receptor (M_2_R) (Figure 10 and Supplemental Figure 35A; clone 1 was used for subsequent experiments), the most abundant muscarinic receptor subtype in the myocardium. In this system, TMAO acted as a significant inhibitor of M_2_R signaling in the presence of the M_2_R agonist carbachol (Figure 10A). Furthermore, heart rate measurements in unanesthetized male and female CREM-IbΔC-X mice demonstrated that choline-supplemented mice had significantly higher heart rates, which were accompanied by elevated plasma TMAO levels (Figure 10B and Supplemental Figure 35). Collectively, these data support a role for TMAO in autonomic dysfunction and the promotion of AF.

## Discussion

The present study reveals both a clinical association between circulating levels of TMAO and AF risk and — through multiple animal model studies — a direct mechanistic link between elevated TMAO and AF onset and persistence that is reversed by pharmacological targeting of the gut microbial TMA/TMAO pathway. The promotion of AF by TMAO may in part be mediated by autonomic dysfunction (Figure 10C). We first confirmed and extended previous clinical data linking TMAO to AF by demonstrating that plasma TMAO, choline, and betaine independently associated with prevalent AF. Using a mouse model of spontaneous AF, we next showed that a choline-supplemented diet causes the CREM-IbΔC-X mouse to develop paroxysmal AF (first time) and persistent AF sooner than chow-fed control mice. Plasma TMAO associated with the onset of both paroxysmal AF and persistent AF. This effect was completely abolished using the TMA lyase inhibitor IMC. IMC is poorly absorbed into plasma and has no off-target effects on liver FMO activity. In contrast, IMC is highly abundant in the gut and effectively inhibited microbial TMA production from choline, resulting in decreased TMAO production and a delay in AF onset. To demonstrate that the effects of TMAO are not strain dependent, we validated that the direct supplementation of TMAO in the male and female C57BL/6J enhanced AF susceptibility. Characterization of the gut microbiome from CREM-IbΔC-X mice with a choline-supplemented diet showed a significant increase in *CutC/D* mRNA counts and altered gut microbial species, demonstrating that the choline-supplemented diet alters gut microbial TMA and its metabolite formation. Consumption of a choline- or TMAO-supplemented diet was significantly associated with atrial enlargement and atrial electromechanical dysfunction. This may in part be precipitated by chronic TMAO inhibition of M_2_R.

The TMAO pathway has been associated with the incidence of multiple CVD phenotypes in both animal models (1, 2, 14, 15, 40) and large clinical cohort studies (41–43). In 2 community-based cohorts, TMAO and its metabolites, including carnitine and choline, were associated with risk of heart failure (21). Similarly, this study extends the association of the TMAO pathway to the pathogenesis of AF in a large clinical biorepository. There are a few clinical studies reporting an association of increased circulating TMAO levels with increased AF risk. A study of 2 Norwegian cohorts with a long-term follow-up period of 7.3 years showed that a baseline elevated plasma TMAO concentration was associated with AF (HR: 1.16, 95% CI: 1.05, 1.28 and HR: 1.10, 95% CI: 1.004, 1.19) (33). In the recent AF-RISK study by Nguyen et al., it was observed that the elevated levels of TMAO associated with clinical AF progression (44). We add to these studies by demonstrating that TMAO is independently associated with prevalent AF in a large clinical cohort at Cleveland Clinic. TMAO is a host–microbiome co-metabolite generated from dietary precursors such as choline, phosphatidylcholine, and l-carnitine through gut microbial metabolism, followed by hepatic oxidation via FMO3 (3, 12). Thus, interindividual variability in TMAO levels may reflect differences in gut microbial composition and functional capacity, in addition to host factors such as liver enzyme activity and renal clearance. Although the absolute difference in TMAO concentrations between tertiles may not appear large, the associated risk for AF is substantial: there is an approximately 2-fold increase in the odds of developing AF. This is analogous to classic cardiovascular risk factors such as LDL-cholesterol, where even modest elevations are associated with significant lifetime risk. These findings underscore that small differences in exposure over long periods can have large cumulative effects on disease risk. Despite these limitations, plasma TMAO remains an independent predictor of AF and other CVD phenotypes (1–3). We also demonstrate for the first time, to our knowledge, that choline and betaine independently associate with prevalent AF in humans.

One of the most remarkable observations of this study was the demonstration that a choline-supplemented diet was associated with the development of both earlier onset paroxysmal AF and persistent AF in the CREM-IbΔC-X mouse model of spontaneous AF. This effect was eliminated with the use of the gut microbial TMA lyase inhibitor IMC, demonstrating that the gut microbiome is mechanistically involved in the pathogenesis of AF. After stratification by sex, the same trend was observed; however, in female mice, the choline diet did not show a statistically significant difference compared with a chow diet for the onset of paroxysmal AF. This was also true of TMAO CREM-IbΔC-X mice on the TMAO-supplemented diet. While CREM-IbΔC-X male and female mice fed the choline diet had a similar onset of paroxysmal AF, chow-fed CREM-IbΔC-X female mice appeared to develop paroxysmal AF sooner than male CREM-IbΔC-X mice. Notably, chow-fed CREM-IbΔC-X female mice had higher circulating plasma TMAO levels than male CREM-IbΔC-X mice. This difference is related to CREM-IbΔC-X female mice producing more TMAO due to higher expression of hepatic FMO3 enzyme than male mice (9). Sex-dependent regulation of hepatic FMO3 expression is already well established. Prior studies have demonstrated that FMO3 expression in mice is markedly higher in females than males (1, 12), largely due to androgen-mediated repression and estrogen-associated upregulation (45, 46). Interestingly, CREM-IbΔC-X mice supplemented with IMC on a chow diet also showed a trend, and in the case of the paroxysmal AF (first time) in female CREM-IbΔC-X mice, a significant delay in the onset of AF. This is likely related to the presence of dietary choline in the chow diet. Notably, despite the differences in TMAO plasma levels in male and female mice, we observed enhanced AF susceptibility in both sexes across 2 different mouse models of AF, possibly suggesting there is a threshold effect of TMAO-inducing AF.

Prevailing evidence suggests the need for future research to study the dietary and pharmacological inhibition of gut microbial metabolites like TMAO formation as a potential treatment strategy for attenuating the development of AF. To our knowledge, this is the first study to demonstrate the use of TMA lyase inhibitors as pharmacologic agents for the treatment of AF. In addition to mouse ECG studies that suggested using IMC to prevent AF and mouse studies that showed IMC reverses TMAO induced atrial remodeling, we also demonstrate that the vast majority of IMC remains in the gut and relatively little circulates in mouse plasma. Moreover, the unaltered conversion of d_9_-TMA to d_9_-TMAO in the liver suggests there are no further effects of IMC on the oxidation of TMAO by FMOs in the liver that could account for lower overall circulating TMAO concentrations. These animal studies support the use of TMA lyase inhibitors in human studies.

Our characterization of the gut microbiome showed that CREM-IbΔC-X mice supplemented with a choline diet had significantly less diversity and an increased abundance of the phylum Firmicutes (GGB29005_SGB41723 and GGB18827_SGB41440) when compared with control groups. Not surprisingly, we observed that relative abundance of both species of the phylum Firmicutes was strongly and positively correlated with the TMA, TMAO, and *CutC/D* levels in CREM-IbΔC-X mice supplemented with different diets. Our study findings are in line with previous studies that found that IMC treatment alters the gut microbiome in favor of the host (5, 15, 47–50). It was observed that the Firmicutes/Bacteroidetes ratio, which has been linked to obesity in human and mice, was significantly suppressed by IMC treatment in mice fed a high-fat diet (47–50). In a previous Schugar et al. observed that IMC significantly reduced the proportions of some microbial taxa, including Clostridiales, that belong to the phylum Firmicutes and that it is significantly associated with plasma TMA and body weight in a mouse model (5). In the current study, the significant positive correlation of the 2 species GGB29005_SGB41723 and GGB18827_SGB41440 from the phylum Firmicutes with the plasma levels of TMA, TMAO, and *CutC/D* may explain the gut microbial alterations with IMC treatment. Interestingly, none of the gut microbial bacterial species associating with the choline diet, TMA, TMAO, or *CutC/D* had an association with the time of onset of paroxysmal AF or persistent AF. The association of *P*. *caecicola* and GGB30408_SGB43438 with the onset of paroxysmal AF and persistent AF warrants further study.

In our study, IMC effectively inhibited TMAO without off-target liver effects, and its long-term impact on the gut microbiota was stable for 5 months. However, AF recurrence following IMC cessation was not studied, as CREM-IbΔC-X is a progressive mouse model of AF leading to permanent AF. Once established, it cannot be reversed due to significant atrial myopathy (51, 52). The mouse model follows a similar pathway of the irreversible nature of human persistent and permanent AF. Although microbiota perturbations have been associated with AF phenotypes, including structural and electrophysiological remodeling, causal and reversible microbiota interventions in AF remain an area for future study.

This study suggests that TMAO is mechanistically involved in the onset and progression of AF. While plasma concentration of TMAO was higher in CREM-IbΔC-X mice supplemented with a choline diet and predicted the onset of both paroxysmal AF and persistent AF, levels of betaine and choline did not reliably predict the onset of paroxysmal AF or persistent AF. This observation suggests a crucial role for TMAO in the progression of AF. To further assess the role of TMAO in the pathogenesis of AF, we provided provisionally supplemented CREM-IbΔC-X mice with a TMAO diet and demonstrate that, similar to CREM-IbΔC-X mice supplemented with choline, TMAO causes mice to develop paroxysmal AF and persistent AF sooner compared with a chow diet. This clearly demonstrates that TMAO is directly involved in the pathogenesis of AF.

We demonstrate that CREM-IbΔC-X-TG female mice supplemented with a choline diet had a shorter cardiac wavelength compared with controls. A shortened wavelength is known to be associated with an increased vulnerability to AF and is a mechanism of reentrant excitation (34–39). Although there was not a statically significant decrease in CV in choline-supplemented mice, a shortened APD (equivalent to effective refractory period) has been implicated in AF in mice supplemented with a high-fat diet (53) and in increased vulnerability of both AF and ventricular tachycardia in humans (36, 39). A longer path length (a larger atrial area) likely from LA dilation is part of the wavelength hypothesis and can also contribute to an increase in stability (e.g., persistent AF) and vulnerability to AF (38). Thus, a combination of both structural and electrophysiological dysfunction is likely contributing to AF in choline-supplemented mice.

Overall, systemic inflammation appears to be a major mechanism in AF promoted by TMAO. Studies show that inflammatory markers like C-reactive protein and IL-6 are positively correlated with LA diameter, suggesting that inflammation may play a role in atrial remodeling and prolong the duration of AF (54). In our study, we did not find an association of a choline diet with systemic serum amyloid A protein in mice, an acute-phase inflammatory protein similar to CRP or IL-6 in humans. TMAO has been implicated in the priming and activation of the NLRP3 inflammasome in studies examining vascular inflammation and dysfunction (55), and work lead by Li and colleagues has demonstrated that there is enhanced activation of NLRP3 inflammasome in patients with paroxysmal AF and persistent AF and that the NLRP3 inflammasome is critical in the pathogenesis of AF in CREM-IbΔC-X mice (56). This suggested a possible link between TMAO, the NLRP3 inflammasome, and AF. We performed comprehensive in vivo and in vitro studies that demonstrate that the mechanism of TMAO-enhanced AF does not appear to be through the NLRP3 inflammasome in CREM-IbΔC-X mice. These observations suggest that TMAO and the priming/activation of the NLRP3 inflammasome is not a universal pathogenic mechanism in CVDs.

A possible pathogenic culprit for the development of atrial myopathy and electromechanical dysfunction is chronic autonomic dysfunction from TMAO inhibition of M_2_R and increasing sympathetic tone. Classically, M_2_R agonists acutely shorten APD and increase AF susceptibility. In contrast, M_2_R acute inhibition prolongs APD and decreases AF susceptibility. However, the long-term effects of M_2_R inhibition are not as well understood. Clinically, inhaled long-acting muscarinic receptor antagonists are associated with worse composite cardiovascular outcomes, including cardiac arrhythmias. In a subgroup analysis, there was significantly increased arrhythmogenic events amongst subjects taking long-acting muscarinic receptor antagonists (57). Another study of antipsychotic drugs, well known to have anticholinergic side effects, first found that subjects that were concomitantly taking anticholinergic mediations had an increase in incident AF. Additionally, subjects that chronically took antipsychotics drugs were found to have an increase in AF incidence. This association was found to correlate with antipsychotic drugs with increasing binding affinity to muscarinic receptors. This effect was most notable with antipsychotic drugs that had a strong affinity for M_2_R blockade, where a 22% increase in AF incidence was noted (58). The pathogenic mechanism responsible for more AF is hypothesized to be a relative increase in sympathetic tone relative to the inhibited parasympathetic pathway. Consistent with autonomic imbalance and increase in sympathetic tone, is the observation that unanesthetized CREM-IbΔC-X mice supplemented with a choline diet had significantly elevated heart rates.

In a classic work by Patterson et al., a complex autonomic interplay between parasympathetic and sympathetic activity demonstrated that shortening of the APD was associated with triggered firing from the canine pulmonary veins and an increase in AF susceptibility (59). Additionally, increases in sympathetic tone are well known to shorten APD and increase susceptibility to AF (60, 61). Together, these data suggest a role of TMAO in autonomic dysfunction via inhibition of the M_2_R, resulting in an increase in sympathetic tone and susceptibility to AF. Short-term exposure of TMAO to ex vivo CREM-IbΔC-X mouse hearts or aEHTs did not cause electromechanical dysfunction. This suggests that TMAO involvement in the pathogenesis of AF may require a longer-term TMAO exposure to cardiomyocytes or that it could produce its effects via other cell types found in the heart or produce its proarrhythmogenic effects via extracardiac mechanisms. Together, these data suggest that TMAO plays a role in autonomic dysfunction via M_2_R inhibition and the resulting increased sympathetic tone and susceptibility to AF.

In conclusion, TMAO promotes AF in a gut microbiota–dependent manner by causing autonomic dysfunction, atrial myopathy, and electromechanical dysfunction. TMAO-promoted AF is inhibited by dietary supplementation with IMC. These data represent a novel link between the gut microbiome and AF and suggest that pharmacological inhibition of the choline catabolic pathway may be a preventive treatment for AF.

## Methods

Additional details may be found in the supplemental materials.

### Sex as a biological variable.

In human participants, sex was considered a biological variable, as demonstrated in the subgroup analysis in Figure 2. In mouse studies, all findings are reported for both sexes except for optical mapping studies. Female CREM-IbΔC-X mice were selected due to relatively higher plasma TMAO levels compared with male mice (1).

### Cardiovascular research subjects.

Plasma TMAO, choline, and betaine concentrations were quantified in subjects by stable isotope dilution liquid chromatography with online electrospray ionization tandem mass spectrometry, as previously described (40). These investigations used archival plasma specimens (*N* = 5,090) from the Cleveland Clinic cohort GeneBank (NCT00590200, ClinicalTrials.gov), a research tissue repository comprising sequential consenting stable subjects undergoing elective cardiac evaluation with connecting clinical longitudinal outcome data (2, 3, 62, 63). Exclusion criteria for the GeneBank included patients with a recent myocardial infarction (<4 weeks) or elevated troponin I (>0.03 mg/dL) at enrollment. Quantitative TMAO, choline, and betaine data were available for all subjects (*N* = 5,090). CVD was clinically defined as having a previous history of documented coronary artery disease, peripheral artery disease, cerebral vascular disease (history of a transient ischemic attack or cerebrovascular accident), history of revascularization (coronary artery bypass graft, angioplasty, or stent), or significant angiographic evidence of coronary artery disease (≥50% stenosis) in at least 1 major coronary artery at the time of coronary angiography. History of AF, heart failure, myocardial infarction, hypertension, and diabetes was adjudicated by medical personnel at the time of subject enrollment into GeneBank.

### Experimental animals.

CREM-IbΔC-X mice were obtained from the laboratory of Na Li (Baylor College of Medicine, Houston, Texas, USA) with permission from Frank U. Müeller Institute of Pharmacology and Toxicology, University of Münster, Münster, Germany. All WT (C57BL/6J) mice were obtained from The Jackson Laboratory. Characteristics of cardiomyocyte-directed overexpression of cAMP response element modulator (CREM-IbΔC-X) mice were described previously (31, 32). CREM-IbΔC-X hemizygous mice were used in experiments and were bred by crossing a hemizygous CREM-IbΔC-X mouse with an FVB/NJ mouse strain (The Jackson Laboratory, JAX-001800) and WT C57BL/6J mice (The Jackson Laboratory, JAX-000664) using needle electrode ECG and a transesophageal pacing technique, respectively, as previously described (64). More details about the experimental groups can be found in Supplemental Methods.

### Mouse model of AF and mouse ECG.

Cardiac rhythm was assessed in hemizygous CREM-IbΔC-X and WT mice (C57BL/6J, The Jackson Laboratory, JAX-000664) using needle electrode ECG and a transesophageal pacing technique, as previously described (52, 65). More details about the ECG recordings can be found in Supplemental Methods.

### Shotgun metagenomics sequencing and bioinformatics analysis.

Gut microbial sequencing was performed using cecum samples from CREM-IbΔC-X mice on chow, choline, chow + IMC, and choline + IMC diets. Quality assessment of metagenomic sequences was performed following established protocols (66). Additional details on the metagenomic sequencing and analysis can be found in Supplemental Methods.

### Quantification of TMAO, TMA, choline, betaine, and IMC.

Stable isotope dilution liquid chromatography–tandem mass spectrometry on a Shimadzu 8050 triple quadrupole mass spectrometer was used to quantify TMAO, TMA, choline, betaine, and IMC from acidified mouse plasma samples using electrospray ionization in positive ion mode with multiple reaction monitoring, as previously described (2, 40, 67).

### Creation and maintenance of EHTs.

We created EHTs from dissociated atrial-like induced atrial-like cardiomyocytes suspended in a fibrin hydrogel between flexible silicon posts (EHT Technologies, DiNAQOR) (68). More details on the preparation and maintenance of EHTs can be found in Supplemental Methods.

### Statistics.

Baseline characteristics, demographics, and laboratory values of GeneBank subjects with and without a history of AF were compared using Wilcoxon’s rank-sum test for continuous variables and Pearson’s χ^2^ test for categorical variables. Pearson’s χ^2^ test was also used to assess the relationship between the proportion of prevalent AF and tertiles of increasing plasma concentrations of TMAO, choline, and betaine. OR of prevalent AF and tertiles of increasing concentrations of plasma TMAO, choline, and betaine with no adjustment, after adjustment for patient characteristics and comorbidities (model 1: age, sex, diabetes mellitus, CVD, systolic blood pressure, current smoker, BMI, and high-sensitivity C reactive protein), and full model adjustment were determined (model 2: model 1 + eGFR). Cubic spline plots were performed using an unadjusted logistic regression analysis of increasing plasma choline, betaine, and TMAO plasma concentrations. In the cubic spline plots, associations were modeled using restricted cubic splines with 3 knots in a logistic regression model, yielding 2 spline degrees of freedom (1 for the linear term and 1 for nonlinearity). Knot locations were placed at the default quantiles for 3 knots (approximately the 10th, 50th, and 90th percentiles of the metabolite distribution). *P* interaction values were obtained from likelihood ratio tests comparing logistic models with and without the interaction between TMAO tertiles and each subgroup factor. Kaplan-Meier analysis with Cox proportional hazards regression was used for time-to-event analysis to determine HR and 95% CIs. Multiple groups were compared with a Kruskal-Wallis test with a Dunn post hoc analysis to determine the significant difference between the medians of multiple independent diet groups, and multiple paired comparison adjustments were performed. The Mann-Whitney test was used to compare the 2 medians of 2 independent groups. Permutational ANOVA was used to compare the β-diversity distributions of different dietary groups. Student’s *t* test was used to compare the means of CV, APD, and wavelength between 2 diet groups, after confirmation of normality using Shapiro-Wilk and Kolmogorov-Smirnov tests. Benjamini-Hochberg false discovery rate correction to adjust *q* values for multiple testing was performed to assess the gut microbial abundance and its association with the onset of paroxysmal AF, persistent AF, and duration of AF. The statistical significance level was set at *P* < 0.05. All data were analyzed using R software version 4.3.3 (R Core Team, 2024) (69, 70) and Prism (GraphPad Software).

### Study approval.

All animal experiments were approved by the Cleveland Clinic IACUC. All human participants from GeneBank gave written informed consent, and the Cleveland Clinic IRB approved the study protocol.

### Data availability.

The data supporting the findings of this study are available within the article and in Supplemental Tables 1–13 and Supplemental Figures 1–35. Values for all data points in graphs are reported in the Supporting Data Values file. The next-generation sequencing data generated in this study have been deposited in a MINSEQE-compliant public database and are available under the NCBI BioProject accession number PRJNA1482443 (https://www.ncbi.nlm.nih.gov/bioproject/?term=PRJNA1482443). All other data supporting the findings of this study are available from the corresponding author upon reasonable request.

## Author contributions

SA conducted the experiments, analyzed the data, and wrote the manuscript. XSL, NS, and KRL conducted experiments, helped design the experiments, analyzed the data, and revised the manuscript. IP, LA, EO, DV, IN, HSK, HM, ZW, JAL, MYT, KM, DPM, SMS, JHR, and VC helped conduct the experiments, analyzed the data, and revised the manuscript. MLM, SVNP, DRVW, JB, MKC, JDS, OW, and SLH helped design the experiments and revised the manuscript. RAK conceived the project, conducted the experiments, analyzed the data, designed the experiments, and wrote the manuscript.

## Conflict of interest

ZW and SLH are named as coinventors on pending and issued patents held by the Cleveland Clinic relating to cardiovascular diagnostics and therapeutics (including AU2019280094B2, US10983100B2, US11911399B2, US10064830B2, AU2017291211B2, US20230091848A1, US20160129075A1, US20190099384A1 US20200138887A1, and WO2026030488A1) and have the right to receive royalty payments for inventions or discoveries related to cardiovascular diagnostics or therapeutics from Cleveland Heart Lab, a fully owned subsidiary of Quest Diagnostics and Zehna Therapeutics. SLH also reports having been paid as a consultant for Zehna Therapeutics and has received research funds from Zehna Therapeutics.

## Funding support

This study is the result of NIH funding, in whole or in part, and is subject to the NIH Public Access Policy. Through acceptance of this federal funding, the NIH has been given a right to make the work publicly available in PubMed Central.

- NIH grant P01 HL158502 (to RAK).
- NIH grants R01 HL167831-A1 and P01HL147823 (to SLH).
- A gift from Richard Morrison (to RAK).

## Supplementary Material

Supplemental data

Unedited blot and gel images

Supporting data values

## Version 1 07/24/2026

In-Press Preview

## Version 2 09/01/2026

Electronic publication
