# Supplementary material for: Gut microbial trimethylamine N-oxide generation promotes risk of atrial fibrillation via muscarinic receptor–mediated autonomic dysfunction
Source: J Clin Invest. 2026 Jul 24;136(17):e201684. doi: 10.1172/JCI201684 (PMC13528925; doi:10.1172/JCI201684)
Supplement: Supplementary file 1 [file jci-136-201684-s216.pdf]

Gut microbial trimethylamine *N*-oxide generation promotes risk of atrial fibrillation via muscarinic receptor–mediated autonomic dysfunction

Selvam Arjunan,[1] Isaiah Pemberton,[1] Xinmin S. Li,[1] Naseer Sangwan,[1] Lydia Akino,[2,3] Emmanuel Opoku,[1] Dmitriy Verbovetskiy,[1] Ina Nemet,[1] Hyun Su Kim,[1] Haruko Masumiya,[1] Zeneng Wang,[1] Joseph A. Lupica,[1] Melissa Y. Tian,[1] Karis Mao,[1] Deepthi P. Mallela,[1] Maradumane L Mohan,[1] Sarah M Schumacher,[1] Julie H. Rennison,[1] Sathyamangla V Naga Prasad,[1] Kenneth R. Laurita,[2,3] Vamsi Chodisetty,[1] Mina K. Chung,[1] David R. Van Wagoner,[1] John Barnard,[1] Jonathan D. Smith,[1] Oussama Wazni,[1,4] Stanley L. Hazen,[1,4] and Robert A. Koeth,[1,4]

[1]Department of Heart, Blood & Kidney Research, Cleveland Clinic Research, Cleveland Clinic, Cleveland, Ohio, USA. [2]Department of Biomedical Engineering, Case School of Engineering School of Medicine, Case Western Reserve University, Cleveland, Ohio, USA. [3]Heart and Vascular Research Center, MetroHealth Campus, Case Western Reserve University, Cleveland, Ohio, USA. [4]Department of Cardiovascular Medicine, Heart, Vascular & Thoracic Institute, Cleveland Clinic, Cleveland, Ohio, USA.

Address for Correspondence:*
Robert Koeth, MD, PhD
Department of Cardiovascular Medicine, and Department of Heart, Blood and Kidney Research, Cleveland Clinic Research, Cleveland Clinic, Cleveland, OH
9500 Euclid Avenue, J2-2
Cleveland, OH 44195
Phone: (216) 445-7610
Fax: (216) 445-6195
E-mail: koethr@ccf.org

Supplemental Materials

Experimental animals.

Male and female mice were divided into 6 experimental groups: chemically defined control diet [1] Chow diet (2018 Teklad Global 18% Protein Rodent Diets – Inotiv), [2] 1.3% Choline diet (C1879 Sigma-Aldrich), [3] 0.06% IMC added to the control diet Chow (Chow + IMC), [4] 0.06% IMC added to 1.3% choline diet (Choline + IMC), [5] TMAO (0.3%) diet (T-0514 Sigma-Aldrich), [6] 0.03% MCC950 (B1370-474692 Boc Science) added to 1.3% choline diet (C1879 Sigma-Aldrich) as previously described (1). All noted dietary supplements were dissolved into mouse water (g/100 ml). Mice were placed on respective diets at the time of weaning (4 weeks of age).

Mouse ECG studies.

Cardiac rhythm was assessed in hemizygous CREM-IbΔC-X mice and WT (C57BL/6J mice - JAX -000664) using needle electrode electrocardiograms (ECG), and transesophageal pacing technique, respectively (2), as previously described (3, 4). Briefly, mice were anesthetized with 2-3% isoflurane in oxygen and maintained with 1-3% isoflurane in oxygen using a portable anesthesia machine. Subdermal needle electrodes (ADI instruments MLA 1214) were used to record ECGs using an ADI Bio Amp and a Powerlab receiver with associated analysis software. ECGs were recorded at 37-38 °C using a heating pad under the surgical board. Electrodes were placed in the limbs, cleaned with 70% ethanol, sub-dermally (5). Digital ECGs were acquired 3 days/week for 5 minutes, at the same time of day. AF occurrence and duration over the monitoring time (time in AF / time monitored) were recorded during each ECG session. Serial changes in heart rate, P-wave morphology and duration, R-R interval and changes in atrial ectopy were monitored. Any detection of AF for the first time was classified as the onset of paroxysmal AF. Persistent AF was defined as 10 consecutive readings of AF with <30 seconds of normal sinus rhythm (6).

Collection and analysis of unanesthetized mouse ECG recordings.

Electrocardiograms (ECGs) were recorded from conscious, unstrained and 6-weeks-old CREM mice, using the ECGenie apparatus (Mouse Specifics, Inc., Framingham, MA). This system comprises a heating light, heart shaped pad, recording tower with electrodes located in the floor, a preamplifier, and analog-to-digital conversion via LabChart and EzCG software version 5.2 (ADInstruments, Colorado Springs, CO). ECGenie was set to record cardiac electrical signals at 60Hz. CREM-IbΔC-X mice were placed on the heart shaped pad of the tower with a heating light

on the top for ECG recording. ECG recordings were obtained when the two front paws of the mouse are on the heart shaped pad. At least three clean readings were recorded from each mouse to determine the heart rate. Selected regions of interest were exported as text files and analyzed using the e-MOUSE platform (Mouse Specifics, Inc.). The software applies automated peak detection algorithms to identify R waves and utilizes advanced computational approaches to delineate P–QRS–T waveforms and calculate interval durations.

Electrophysiological study in C57BL/6J mice.

For electrophysiological studies, an octapolar catheter (ADInstruments: ERP800) was inserted through the esophagus (transesophageal) of the C57BL/6J mice, a well-characterized and mostly commonly used laboratory strain in the biomedical research, maintained under 2–3% isoflurane with controlled body temperature (2). Programmed electrical stimulation was performed using a modified burst pacing protocol to induce AF (2). A modified two-phase burst stimulation protocol was used to induce atrial arrhythmias, consisting of 10 phase I preconditioning stimuli (cycle length 60 ms) followed by phase II burst stimuli (cycle length 20 ms), with a 30 second interval between phases; this programmed stimulation sequence was repeated 10 times (each sequence represents a "trial"). AF was defined as rapid, disorganized atrial activity with irregular ventricular rhythm lasting ≥2 seconds. Total 10 trials per mouse, unless AF lasts for 5 min. Data acquisition and analysis were conducted using LabChart software. Power analyses were performed a priori to ensure adequate statistical power for detecting differences in cardiac function and arrhythmia incidence.

Echocardiography.

Echocardiography was performed on CREM-IbΔC-X mice at 8 weeks of age following 5 weeks of the indicated diet supplementation. Briefly, mice were anesthetized with 2-3% isoflurane in oxygen and maintained with 1-3% isoflurane in oxygen using a portable anesthesia machine. Echocardiography was performed using the Vevo 2100 imaging system from VisualSonics (7). Briefly, two-dimensional echocardiographic views of the mid-ventricular short axis were obtained at the level of the papillary muscle tips below the mitral valve. M-mode measurements were determined at the plane bisecting the papillary muscles to assess heart and chamber dimensions. The left atrial diameter size was measure in systole and diastole conditions as described (4) and LV functions (LV mass, LV EF, Cardiac Output, Fractional Shortening and Heart Rate) were analyzed (7).

Whole heart optical action potential mapping.

CREM-IbΔC-X mice were injected with 500 units heparin and anesthetized via intraperitoneal injection of ketamine (100 mg/Kg)/xylazine (10 mg/kg) mixture. Hearts were excised and Langendorf perfused with oxygenated Tyrode solution containing (mM): NaCl 140, KCl 4.5, $CaCl_2$ 1.25, $MgCl_2$ 0.7, HEPES 10, and dextrose 5.5 (pH 7.4), maintained at 34–36°C by placing the solution in a heated custom-built Plexiglas chamber, as described (8). During the experiment, electrocardiograms, perfusion pressure maintained at 40–60 mmHg and bath temperature were measured continuously. Blebbistatin (6 μM) was added in the Tyrode solution to reduce motion artifacts during optical recordings. To optically map electrical activity, hearts were perfused with a bolus of the voltage-sensitive dye di-4-ANEPPS (30 μM). Optical action potentials were recorded simultaneously from the posterior atria within a 10×10 mm mapping field of view. Fluorescence was excited with uniform light from a high-power green LED filtered at 514 ± 5 nm, then collected using a custom, high magnification tandem lens assembly and transmitted (emission filter >610 nm) to a CMOS camera (MiCAM ULTIMA, SciMedia, Costa Mesa, CA). Frames (100×100 pixels) were acquired at a rate of 2000 Hz and binned in software (5×5) yielding an equally spaced 20×20 array of action potential recordings with 0.5 mm inter-pixel spatial resolution. Other than pixel binning, no additional spatial or temporal filtering was utilized. In every mouse heart, steady state atrial epicardial pacing was performed at cycle lengths ranging from 130 to 60 ms with 10 ms decrements. Arrhythmia inducibility was determined by programmed electrical stimulation with up to three premature stimuli (S1-S4 pacing protocol), rapid pacing, and an isoproterenol challenge (400 nM). To determine AF electrophysiological substrates, action potential activation times and local conduction velocity (CV) vectors were calculated as previously described (8). Additionally, atrial action potential duration (APD) APD 80 was calculated as the time difference between activation time and when the membrane potential returned to 80% of repolarization. Local CV and APD80 data were acquired from the left atrium (LA), atrial posterior wall (PW), and right atrium (RA). Cardiac wavelength was calculated as the product of mean APD 80 and CV in each animal.

Optical mapping of EHT action potentials and Ca2+ transients was performed using a custom designed inverted macroscope that we have described in detail previously (9). For pacing, field stimulation (IonOptix C-Pace EM) was used. EHTs on silicone racks were placed in 24-well tissue culture plates for all recordings. The CCD recording camera (MiCam02-HR, SciMedia, Costa Mesa, CA) was configured for 20 x 14 binned pixels for each EHT yielding a spatial resolution of

0.38 x 0.43 mm at a rate of 500 frames/sec. For Ca2+ transient and action potential recordings, EHTs were incubated with 5 μM Fluo-4 (Sigma/Aldrich) or 0.5x FluoVolt (Sigma/Aldrich) for 45 minutes. After incubation, all EHTs were washed with normal Tyrode's solution containing (mM) NaCl 134, KCl 2.9, $NaHCO_3$ 12, Na_2HPO_4 0.34, $CaCl_2$ 1.2, $MgCl_2$ 1.0, HEPES 10, and glucose 10 (pH 7.4) before recordings were performed. Experiments were performed at 34-36°C using a transparent heating plate underneath the multi-well plate. Blebbistatin (10 μM) was added in the Tyrode solution to reduce motion artifacts during optical recordings. Either Ca2+ or action potentials were recorded during intrinsic rhythm and then recorded during steady state pacing at 1.7 and 2.1 Hz. CV and APD50 were determined similarly as that described above.

Liver histology.

Liver tissues were harvested from CREM-IbΔC-X mice fed chow, choline-supplemented, chow + IMC, or choline-supplemented in combination IMC to analyze effect of these diets on liver tissues as previously described (10) . Mice were euthanized and liver tissues were fixed for 1–3 days in 4% paraformaldehyde at 4 °C. Following dehydration, tissues were paraffinized using a Microm STP 120 tissue processor (ThermoFisher), embedded in paraffin and sectioned. Liver sections were then de-paraffinized and stained with Hematoxylin and Eosin (H&E), Picrosirius Red, and Masson's Trichrome as previously described (11). Tissues were dehydrated with increasing ethanol concentrations followed by xylene and mounted with Permount. An Aperio AT2 digital whole slide scanner (Leica Biosystems) was used to scan tissue sections automatically using Image-Pro 10 software (Media Cybernetics).

Shotgun metagenomics sequencing and bioinformatics analysis.

We prepared metatranscriptomic libraries using the QIAseq FastSelect rRNA Removal Kit combined with the QIAseq Stranded mRNA Library Kit (QIAGEN) following established protocols [citations]. We started with 150 ng of high-quality total RNA extracted from samples using the RNeasy PowerMicrobiome Kit (QIAGEN) to isolate microbial RNA. We depleted rRNA using the QIAseq FastSelect 5S + 16S/18S + 23S/28S Kit to remove abundant ribosomal RNA, followed by mRNA enrichment. We fragmented the enriched RNA to an average size of 300 bp using a Covaris M220 focused-ultrasonicator with settings: duty factor 10%, peak incident power 50 W, 200 cycles per burst, 60 s treatment time. We purified fragmented RNA using QIAseq Beads (1.8× ratio).

We performed first-strand cDNA synthesis using random hexamers and reverse transcriptase at 25 °C for 10 min, followed by 42 °C for 50 min and 70 °C for 15 min. Second-strand cDNA synthesis was carried out with DNA polymerase. We then performed end-repair, A-tailing, and adapter ligation in a single reaction using the QIAseq Stranded Enzyme Mix at 20 °C for 15 min followed by 65 °C for 15 min. We used QIAseq Unique Dual Index Adapters (QIAGEN) for multiplexing. We cleaned ligated products with QIAseq Beads (0.8× ratio). We enriched libraries by PCR in 50 µL reactions containing 25 µL of QIAseq HiFi Master Mix, 5 µL of QIAseq IL-Index Primers, and 15 µL of ligated cDNA. Cycling conditions were 98 °C for 30 s, followed by 12–15 cycles of 98 °C for 15 s, 60 °C for 30 s, and 72 °C for 30 s, with a final extension at 72 °C for 1 min. We performed size selection using dual QIAseq Bead cleanup (0.6× followed by 1.0×) to retain fragments of 200–500 bp.

We quantified libraries using the Qubit RNA HS Assay and assessed size distribution on a Bioanalyzer High Sensitivity RNA or DNA chip. We pooled indexed libraries at equimolar concentrations and performed a final QIAseq Bead cleanup (0.8×). We validated the final pool by qPCR using the QIAseq Library Quant Assay Kit and diluted it to 1.8 nM. We denatured the pool with 0.2 N NaOH, neutralized it, and further diluted it to 300 pM. We loaded 1.3 mL onto an Illumina NovaSeq 6000 S4 flow cell and sequenced it using an S4 Reagent Kit (150–300 cycles) for 2 × 150 bp paired-end sequencing, targeting ~15 million read pairs per sample.

We assessed raw FASTQ quality using FastQC v0.11.9. We trimmed adapters and low-quality bases (Phred < 20) using Trimmomatic v0.407 with parameters: ILLUMINACLIP: QIAseq_adapters.fa:2:30:10, LEADING:20, TRAILING:20, SLIDINGWINDOW: 4:20, MINLEN:50. We removed contaminant host reads by mapping against the reference transcriptome or genome (e.g., GRCm39) using HISAT2 v2.2.1 with very-sensitive-local settings (https://sourceforge.net/projects/bbmap/) and discarding aligned reads (12). Subsequently, filtered reads were used for taxonomic and functional characterization using MetaPhlAn4 and HUMAnN3, respectively (13). For differential abundance analyses, we employed the random forest methodology integrated within the DAtest package. This involved comparing various differential abundance detection methods based on metrics such as the false discovery rate (FDR), area under the curve (AUC), empirical power, and the false positive rate (FPR). Our benchmarking analysis indicated that lefseq and metagenomeSeq were the preferred methods for conducting these abundance assessments. Throughout the analysis, we maintained a statistical significance threshold of $P<0.05$. When necessary, we adjusted P-values to account for

multiple comparisons, applying the Benjamini and Hochberg approach to mitigate the FDR. The statistical significance for individual pathways was evaluated using Tukey's Honest Significant Difference (HSD) test. Additionally, species abundance was analyzed in relation to metadata variables through both linear regression (a parametric test) and the Wilcoxon rank-sum test (a non-parametric alternative), utilizing native functions in R (version 4.1.2; R Core Team, 2021). The next-generation sequencing data generated in this study have been deposited in a MINSEQE-compliant public database and are available under the NCBI BioProject accession number PRJNA1482443.

Quantification of choline TMA lyase (*CutC/D*) activity and its inhibition with IMC in cecum microbial communities.

Metabolic transformation of choline into TMA (choline TMA lyase or *CutC/D* activity) was measured as previously described (14). Briefly, d_9-TMA production was monitored from d_9-choline incubated with intestine segments from CREM-IbΔC-X mice on chow, choline, chow+IMC and choline+IMC diets using stable-isotope-dilution LC-MS/MS analysis. Frozen cecum samples (kept on dry ice) were sectioned into two halves, each weighed (one half for aerobic conditions and other half for anaerobic conditions). IMC concentration in CREM-IbΔC-X mouse intestinal segments (cecum) on chow, choline, chow+IMC and choline+IMC diets were monitored using LC-MS/MS (15).

Measurement of FMOs activity in liver homogenate.

CREM-IbΔC-X mouse liver tissue (~20 mg) samples on chow, choline, chow+IMC and choline+IMC diets were homogenized with protease inhibitor cocktails. Soluble protein was incubated with d_9-TMA in HEPES (10 mM, pH 7.4) containing NADPH. The reaction was stopped after 8 h with formic acid followed by filtering through a 3 kDa cutoff spin filter (UFC500396, Amicon Ultra, Millipore, Temecula, CA, USA). d_9-TMAO was measured in filtrated by LC-MS/MS as previously described (16). FMOs activity was calculated in pmol d_9-TMAO/mg protein/h.

Stable cell lines.

HEK cells were purchased from American Type Culture Collection (ATCC, Manassas, VA, USA) stably overexpressing M2R was used in this study. A stable cell line was developed in-house by clonal selection using G418 (Geneticin - Gibco-10131-035) as antibiotic selection medium after transfecting HEK 293 cells with the mammalian expression vector plasmid CHRM2_OHu10556C_pcDNA3.1(+)-N-HA (M_2R) purchased from Genscript (17).

Cell culture.

For optimal growth of cells, Primary cardiomyocytes (MEM media, Gibco 11575-032), HA-CHRM2-HEK 293 cells (M_2R) (DMEM 11-500 + G418 (Geneticin, Gibco-10131-035), Human Cardiac Fibroblast (Lonza Biosciences CC-2904) (DMEM 11-500 was purchased from Cleveland Clinic Research, cell and media production core facility) supplemented with 10% FBS and 5% penicillin-streptomycin (were purchased from Cleveland Clinic Research, cell and media production core facility) was used. Cells were incubated at 37 °C and 5% CO_2. Cells were treated with control (water) or TMAO at the concentration of 150 μM, 300 μM and 1 mM for 24h.

Differentiation of iPSC-derived cardiomyocytes into a-iCMs.

Differentiation of induced pluripotent stem cell (iPSC)-derived cardiomyocytes to atrial-like myocytes (iCMs) was adapted from the ventricular predominant cardiomyocyte differentiation protocol of Burridge and colleagues (18), with inclusion of retinoic acid (1 μM) in the differentiation media from days 4-10 to enhance atrial-like differentiation (19). A metabolic selection step (no glucose, 4 mM lactate) was used from days 12-14 to minimize the abundance of non-cardiomyocytes in the cultures. Using this method, beating iCMs are typically evident by day 8.

Creation and maintenance of atrial like engineered heart tissues (aEHTs).

We created engineered heart tissues (aEHT) from dissociated atrial-like iCMs suspended in a fibrin hydrogel between flexible silicon posts (EHT Technologies DiNAQOR, Hamburg, Germany) (20). aEHTs were prepared under sterile conditions using 24-well tissue culture plates (21). Agarose (2%) was placed in each well. Teflon spacers were placed into the warm agarose, then removed from solidified agarose, and racks with silicone posts were placed in the agarose wells.

Atrial-like iCMs were dissociated on or around day 15 of differentiation using collagenase (Type 2; 1 mg/mL) (Worthington Biochemical, Lakewood, NJ) and TrypLE (ThermoFisher, Carlsbad, CA). aEHTs were created using 575,000 iCMs per construct suspended in a 100 μl aliquot of fibrinogen hydrogel. This suspension was mixed with thrombin and quickly pipetted into the agarose wells, then incubated at 37°C, 5% CO_2, 98% relative humidity for 2 hours. After addition of media to the wells, the silicone racks were removed from the agarose wells and transferred to a new 24-well plate containing pre-warmed EMT-media (21). aEHTs were incubated at 37°C, 5% CO_2, 40% O_2 and fed every other day with EMT media. Downstream protocols were performed 30 days after aEHT formation (45 days of differentiation).

Contractile Activity.

EHT contractility data were acquired with real-time edge detection using IonWizard software and analyzed with CytoSolver 3.1 software (both from IonOptix, Westwood, MA). Tissue culture plates were placed in an Oko-Labs Stagetop Incubator with a Digital Gas Composition Mixer (5% CO_2) and temperature control (37°C). Following EHT formation, contractile activity was monitored weekly to assess quality of constructs. For drug studies, EHTs were randomized into vehicle (water) or 10 mM TMAO treatment for 48 hours. During this period, contractility was assessed daily.

Measurement of calcium in TMAO mediated modulation of M_2R signaling.

M_2R-induced intracellular calcium mobilization was assessed in HEK-293T cells stably expressing M_2R (Clone -1), providing a receptor-specific model in a widely used human cell system. Calcium flux was measured using the Fluorescent Imaging Plate Reader Calcium 6 Assay Kit (Molecular Devices) following established protocols(17). Cells were loaded with Calcium 6 dye and treated with carbachol (positive control) or TMA/TMAO (300 μM) at 37 °C, and fluorescence changes were recorded using a FlexStation-3 microplate reader. Baseline fluorescence was acquired prior to compound addition, followed by real-time measurement of calcium responses over 120 s to capture peak signaling. A non-cumulative dose-response design was employed, with each concentration tested in triplicate wells. Intracellular calcium changes were quantified as ΔF/F and expressed as relative fluorescence units (RFU). Assay reproducibility and variability were evaluated across six independent experiments. Concentration–response curves were fitted using nonlinear regression in GraphPad Prism to determine EC_{50}/IC_{50} values. Metabolite concentrations were selected based on physiologically relevant ranges reported in the Human Metabolome Database (https://www.hmdb.ca/) and prior literature (3). Since disease-associated levels of TMAO have not been established, a ~100-fold higher concentration (300 μM) was used in this study.

Western blot analysis.

Protein lysates were prepared using T-PER buffer (ThermoFisher, catalog #78510) supplemented with Halt protease and phosphatase inhibitors (ThermoFisher, catalog #78440). Equal amounts of protein (10μg/lane) were separated by SDS-PAGE and transferred onto nitrocellulose membranes. Membranes were blocked with Intercept blocking buffer (LICOR Biosciences, catalog #927-70001) and incubated with primary antibodies: HA Tag Antibody (Roche Diagnostics

GmbH, clone #12CA5) and GAPDH (Santa Cruz, catalog #SC-25778) as internal control. Appropriate goat anti-mouse (Invitrogen, catalog#31430) and goat anti-rabbit secondary antibodies (Invitrogen, catalog#31460) were applied, and chemiluminescent substrates (Cytiva, RPN2232) were used to detect signals. Band intensities were visualized by manual film development in a dark room.

Quantitative RT-PCR.

Total RNA was isolated from the cell lines treated with TMAO using the Qiagen RNeasy Mini Kit according to the manufacturer's instructions (Qiagen). A total of 1,000 µg of RNA was used for cDNA using a SuperScript IV Vilo Master Mix w/ezDNase (Life Technologies Corp 11766050). qPCR was performed in triplicate in 96-well plates with TaqMan Fast Advanced Master Mix (Applied Biosystems 4444554). Relative mRNA transcript levels (normalized to GAPDH) were calculated using the CT (cycle number) method and are reported as fold change. The sequences of the primers used in the study were: NLRP3 - Mm00840904_m1 (Mouse NLRP3), Hs00918082_m1 (Human NLRP3) and IL-β - Mm00434228_m1 (Mouse IL1-Beta), Hs01555410_m1 (Human IL1-Beta) (Life Technologies Corp).

Isolation of mouse cardiomyocytes and fibroblast.

The heart was excised under sterile conditions from CREM-IbΔC-X mice during isoflurane anesthesia. After excision, the heart was cannulated and perfused in Langendorff mode with perfusion buffer containing (in mmol/L): NaCl 113, KCl 4.7, KH_2PO_4 0.6, Na_2HPO_4 0.6, $MgSO_4$ 1.2, HEPES 10, $NaHCO_3$ 12, $KHCO_3$ 10, BDM (2,3-Butanedione monoxime) 10, Taurine 30 and Glucose 5.5 at pH 7.0 for 5-10 minutes, 37 °C. Heart tissue were then digested with 1U Collagenase Type II (Cat.# LS004176, Worthington Biochemical Corporation) at 37 °C until digestion was complete. After digestion, the atria were dissected and minced with collagenase solution. Tissues were gently dissociated with sterile plastic transfer pipettes of different size opening (1.5mm and 1mm diameter) for 3- 5 min. Cells were centrifuged at 20 *g* x 3 min (Eppendorf 5810R) to separate the nonmyocytes such as fibroblast, endothelial cells and some round myocytes. Supernatant was aspirated and then the pellet was resuspended with calcium solution-1 with stop buffer (12.5 µM- $CaCl_2$, stop solution – 45 ml sterile perfusion buffer with 5 ml FBS) and 100 µl of 200 mM ATP (pH 7.2) until final concentration of 2 mM ATP and spun at 20 *g* x 3 min. The above step was repeated using calcium solution II (100 µM- $CaCl_2$, Stop solution –

45 ml sterile perfusion buffer with 5 ml FBS) and 100 µl of 200 mM ATP (pH 7.2) until final concentration of 2 mM ATP), calcium solution III (400 µM - $CaCl_2$, Stop solution – 45 ml sterile perfusion buffer with 5 ml FBS) and 100 µl of 200 mM ATP (pH 7.2) until final concentration of 2 mM ATP) and calcium solution IV (900 µM - $CaCl_2$, Stop solution – 45 ml sterile perfusion buffer with 5 ml FBS) and 100 µl of 200 mM ATP (pH 7.2) until final concentration of 2 mM ATP) with ATP added. Then the rod-shaped myocytes were separated and seeded into the culture medium containing 49 ml MEM medium (Gibco 11575-032), 0.5 ml penicillin/streptomycin (10,000 U/ml) and 0.5 ml of 100 mg/ml BSA (Sterile)(18). These cardiomyocytes were used for further treatment such as TMAO at various concentrations.

Inhibition of NLRP3 inflammasome via *in vivo* LPS challenge.

CREM-IbΔC-X mice on chow diet were either treated with i.p. injection of 10 mg/kg LPS Escherichia coli 055: B5 (Sigma Aldrich) or PBS. While CREM-IbΔC-X mice on 1.3% choline diet (C1879 Sigma-Aldrich) were treated with 0.3 mg/ml of MCC950 (B1370-474692 Boc Science) for a week in drinking water and after a week, 10 mg/kg LPS Escherichia coli 055: B5 (Sigma Aldrich) or PBS were injected intraperitoneally. Two hours after injection, CREM-IbΔC-X mice were sacrificed and plasma levels of IL-1β (abcam, catalog#ab108866), TNF-α (Becton Dickinson, catalog#560478), IL-6 (abcam, catalog#ab100712) and SAA (abcam, catalog#ab215090) were measured using the Enzyme-Linked Immunosorbent Assay Kit (ELISA).

Supplemental Tables of the hazard ratios of spontaneous AF. Hazard ratios (HR) between respective dietary groups in Supplementary Tables 1-9 were calculated using a Cox proportional hazard model.

Supplemental Table 1: Hazard ratios of the incidence of paroxysmal and persistent AF in CREM-IbΔC-X mice on TMAO diet

Group Comparisons	HR (95% CI)	*P* Value
Chow Vs TMAO	1.7 (1.02 - 3.0)	0.03

Supplemental Table 2: Hazard ratios of the incidence of persistent AF in CREM-IbΔC-X mice on TMAO diet

Group Comparisons	HR (95% CI)	*P* Value
Chow Vs TMAO	3.6 (2.0 – 6.8)	<0.01

Supplemental Table 3: Hazard ratios of the incidence of paroxysmal AF in male and female CREM-IbΔC-X mice on Chow and TMAO supplemented diets

Male paroxysmal AF

Group Comparisons	HR (95% CI)	*P* Value
Chow Vs TMAO	2.5 (1.1 - 5.7)	0.03

Female paroxysmal AF

Group Comparisons	HR (95% CI)	*P* Value
Chow Vs TMAO	1.3 (0.6 - 2.7)	0.47

Supplemental Table 4: Hazard ratios of the incidence of persistent AF in male and female CREM-IbΔC-X mice on Chow and TMAO supplemented diets

Male persistent AF

Group Comparisons	HR (95% CI)	*P* Value
Chow Vs TMAO	3.6 (1.4 - 9.1)	<0.01

Female persistent AF

Group Comparisons	HR (95% CI)	*P* Value
Chow Vs TMAO	3.0 (1.3 - 7.0)	<0.01

Supplemental Table 5: Hazard ratios of the incidence of paroxysmal AF in CREM-IbΔC-X mice on respective diets

Group Comparisons	HR (95% CI)	*P* Value
Choline Vs Chow	2.0 (1.2 – 3.3)	<0.01
Chow Vs Chow+IMC	1.8 (1.1 – 3.0)	0.01
Choline Vs Choline+IMC	4.2 (2.5 – 7.2)	<0.01
Chow+IMC Vs Choline+IMC	1.0 (0.7 – 1.8)	0.66

Supplemental Table 6: Hazard ratios of the incidence of persistent AF in CREM-IbΔC-X mice on respective diets

Group Comparisons	HR (95% CI)	*P* Value
Choline Vs Chow	2.8 (1.7 – 4.6)	<0.01
Chow Vs Chow+IMC	1.4 (0.8 – 2.3)	0.20
Choline Vs Choline+IMC	3.7 (2.2 – 6.4)	<0.01
Chow+IMC Vs Choline+IMC	1.0 (0.6 – 1.6)	0.89

Supplemental Table 7: Hazard ratios of the incidence of paroxysmal AF in male and female CREM-IbΔC-X mice on respective diets

Male paroxysmal AF

Group Comparisons	HR (95% CI)	*P* Value
Choline Vs Chow	2.5 (1.2 – 5.0)	<0.01
Chow Vs Chow+IMC	1.5 (0.8 – 2.9)	0.24
Choline Vs Choline+IMC	5.0 (2.3 – 11)	<0.01
Chow+IMC Vs Choline+IMC	1.3 (0.7 – 2.7)	0.43

Female paroxysmal AF

Group Comparisons	HR (95% CI)	*P* Value
Choline Vs Chow	1.4 (0.7 – 2.7)	0.39
Chow Vs Chow+IMC	3.4 (1.6 – 7.3)	<0.01
Choline Vs Choline+IMC	3.5 (1.7 – 7.2)	<0.01
Chow+IMC Vs Choline+IMC	0.8 (0.4 – 1.6)	0.46

Supplemental Table 8: Hazard ratios of the incidence of persistent AF in male and female CREM-IbΔC-X mice on respective diets

Male persistent AF

Group Comparisons	HR (95% CI)	*P* Value
Choline Vs Chow	2.5 (1.2 – 5.2)	0.02
Chow Vs Chow+IMC	1.4 (0.7 – 2.9)	0.39
Choline Vs Choline+IMC	3.6 (1.7 – 7.7)	<0.01
Chow+IMC Vs Choline+IMC	1.0 (0.5 – 2.2)	0.91

Female persistent AF

Group Comparisons	HR (95% CI)	*P* Value
Choline Vs Chow	4.0 (1.9 – 8.7)	<0.01
Chow Vs Chow+IMC	1.6 (0.8 – 3.4)	0.19
Choline Vs Choline+IMC	4.5 (2.1 – 1.0)	<0.01
Chow+IMC Vs Choline+IMC	0.7 (0.3 – 1.5)	0.34

Supplemental Table 9: Hazard ratios of the duration of paroxysmal AF in CREM-IbΔC-X mice

**Group Comparisons**	**HR (95% CI)**	***P* Value**
Choline Vs Chow	1.8 (1.1 – 2.9)	0.02
Chow Vs Chow+IMC	1.0 (0.6 – 1.7)	0.89
Choline Vs Choline+IMC	1.5 (0.9 – 2.5)	0.09
Chow+IMC Vs Choline+IMC	0.8 (0.5 – 1.4)	0.45

Supplemental Table 10: Metagenomic association at taxonomical level

Genus	Estimate	Statistic	*P* - value	Method	Alternative
Alistipes_sp	0.363909774	18612	0.00610192	Spearman's rank correlation rho	two.sided
Anaerotruncus_ sp_1XD42_93	0.284757348	20928	0.03376584	Spearman's rank correlation rho	two.sided
Bacteria_unclassified_ SGB41677	-0.64485383	48128.42	8.14E-08	Spearman's rank correlation rho	two.sided
Bacteroidales_bacterium	-0.266966308	37071.43	0.04670179	Spearman's rank correlation rho	two.sided
Candidatus_Arthromitus_ sp_SFB_mouse	0.274339381	21232.83	0.04074614	Spearman's rank correlation rho	two.sided
Clostridiaceae_bacterium	-0.564522383	45777.92	5.85E-06	Spearman's rank correlation rho	two.sided
Emergencia_sp_1XD21_10	-0.268421092	37114	0.04547346	Spearman's rank correlation rho	two.sided
GGB25052_SGB36974	-0.308848094	38296.9	0.02055737	Spearman's rank correlation rho	two.sided
GGB27476_SGB39769	-0.383079599	40468.91	0.00356709	Spearman's rank correlation rho	two.sided
GGB27849_SGB40283	-0.27696514	37364	0.0391192	Spearman's rank correlation rho	two.sided
GGB27866_SGB40300	-0.482843472	43388	0.00019639	Spearman's rank correlation rho	two.sided
GGB27873_SGB41457	-0.28880384	37710.4	0.0308738	Spearman's rank correlation rho	two.sided
GGB27892_SGB40326	0.368284347	18484	0.00548399	Spearman's rank correlation rho	two.sided
GGB28802_SGB41455	0.540943406	13432	1.68E-05	Spearman's rank correlation rho	two.sided
GGB28819_SGB41474	0.302258807	20415.91	0.02356637	Spearman's rank correlation rho	two.sided
GGB28823_SGB41479	0.321532982	19851.94	0.01567433	Spearman's rank correlation rho	two.sided
GGB28853_SGB41519	0.504622399	14494.75	7.32E-05	Spearman's rank correlation rho	two.sided
GGB28889_SGB41570	0.365541626	18564.25	0.00559964	Spearman's rank correlation rho	two.sided
GGB28898_SGB41580	0.373776825	18323.29	0.00454478	Spearman's rank correlation rho	two.sided
GGB28908_SGB41656	-0.313391645	38429.84	0.01867783	Spearman's rank correlation rho	two.sided
GGB28934_SGB41635	0.382095345	18079.89	0.00366089	Spearman's rank correlation rho	two.sided
GGB28946_SGB41652	-0.626572676	47593.52	2.40E-07	Spearman's rank correlation rho	two.sided
GGB28948_SGB41654	-0.380343269	40388.84	0.00383327	Spearman's rank correlation rho	two.sided
GGB28949_SGB41655	-0.310208134	38336.69	0.01997865	Spearman's rank correlation rho	two.sided
GGB28950_SGB41657	-0.321429773	38665.04	0.01570965	Spearman's rank correlation rho	two.sided
GGB28955_SGB41663	0.26723332	21440.75	0.04647433	Spearman's rank correlation rho	two.sided
GGB28956_SGB41665	-0.346606618	39401.71	0.00887235	Spearman's rank correlation rho	two.sided
GGB28959_SGB41668	-0.354706678	39638.72	0.00731085	Spearman's rank correlation rho	two.sided

GGB28960_SGB41669	-0.555408395	45511.25	8.87E-06	Spearman's rank correlation rho	two.sided
GGB28967_SGB41417	0.296035543	20598	0.02711272	Spearman's rank correlation rho	two.sided
GGB28967_SGB41678	-0.567959437	45878.49	4.98E-06	Spearman's rank correlation rho	two.sided
GGB28980_SGB41692	-0.316918477	38533.03	0.01732148	Spearman's rank correlation rho	two.sided
GGB28981_SGB41693	0.28760299	20844.74	0.03160911	Spearman's rank correlation rho	two.sided
GGB28988_SGB41701	0.359256547	18748.15	0.00654349	Spearman's rank correlation rho	two.sided
GGB29002_SGB41718	-0.287593883	37675	0.03161474	Spearman's rank correlation rho	two.sided
GGB29003_SGB41719	0.2763005	21175.45	0.03927198	Spearman's rank correlation rho	two.sided
GGB29005_SGB41723	0.277432007	21142.34	0.03844165	Spearman's rank correlation rho	two.sided
GGB29006_SGB41724	-0.272364585	37229.39	0.04227634	Spearman's rank correlation rho	two.sided
GGB29468_SGB42247	0.279109677	21093.25	0.03723726	Spearman's rank correlation rho	two.sided
GGB29546_SGB42338	0.27757801	21138.07	0.03833557	Spearman's rank correlation rho	two.sided
GGB30300_SGB43264	0.411756924	17211.99	0.00161629	Spearman's rank correlation rho	two.sided
GGB30302_SGB43266	0.33568564	19437.84	0.01143088	Spearman's rank correlation rho	two.sided
GGB30408_SGB43438	-0.295669358	37911.29	0.02693865	Spearman's rank correlation rho	two.sided
GGB30414_SGB43453	-0.59451152	46655.41	1.36E-06	Spearman's rank correlation rho	two.sided
GGB30463_SGB102197	0.284166141	20945.3	0.03379465	Spearman's rank correlation rho	two.sided
GGB31256_SGB44562	0.31493992	20044.86	0.01807166	Spearman's rank correlation rho	two.sided
GGB31793_SGB45163	0.319556506	19909.78	0.01636267	Spearman's rank correlation rho	two.sided
GGB42586_SGB59781	-0.387791374	40606.78	0.00314685	Spearman's rank correlation rho	two.sided
GGB62427_SGB41546	-0.271018455	37190	0.0436635	Spearman's rank correlation rho	two.sided
GGB74408_SGB43542	-0.296631741	37939.44	0.02642224	Spearman's rank correlation rho	two.sided
GGB75053_SGB43494	0.362904054	18641.43	0.00598012	Spearman's rank correlation rho	two.sided
GGB75900_SGB102322	0.520710868	14024	5.03E-05	Spearman's rank correlation rho	two.sided
GGB79698_SGB41545	0.348498803	19062.93	0.00848376	Spearman's rank correlation rho	two.sided
GGB79804_SGB41650	-0.303082396	38128.19	0.02317107	Spearman's rank correlation rho	two.sided
GGB79854_SGB41662	-0.45335152	42525.07	0.00045009	Spearman's rank correlation rho	two.sided
GGB79854_SGB41664	-0.553589806	45458.04	9.62E-06	Spearman's rank correlation rho	two.sided
GGB79857_SGB42426	-0.363233541	39888.21	0.00593138	Spearman's rank correlation rho	two.sided
GGB79948_SGB41679	-0.333888748	39029.58	0.01190771	Spearman's rank correlation rho	two.sided

GGB9819_SGB40890	0.327614491	19674	0.01406399	Spearman's rank correlation rho	two.sided
Lachnospiraceae_bacterium MD335	-0.268957901	37129.71	0.04502695	Spearman's rank correlation rho	two.sided
Muribaculaceae_ bacterium_Isolate_104_HZI	-0.299061734	38010.55	0.02515519	Spearman's rank correlation rho	two.sided
Neglectibacter_sp_X4	0.289405332	20792	0.03087485	Spearman's rank correlation rho	two.sided
Parabacteroides_goldsteinii	0.327546138	19676	0.01408496	Spearman's rank correlation rho	two.sided
Paramuribaculum_intestinale	-0.270198223	37166	0.04432322	Spearman's rank correlation rho	two.sided
Prevotella_sp_MGM1	0.315379357	20032	0.01827283	Spearman's rank correlation rho	two.sided

Supplemental Table 11: Metagenomic association at functional level

Pathway	Estimate	Statistic	*P* - value	Method	Alternative
ANAEROFRUCAT-PWY: homolactic fermentation	0.301379231	20441.64369	0.02399481	Spearman's rank correlation rho	two.sided
CITRULBIO-PWY: L-citrulline biosynthesis	0.266233418	21470.01018	0.047330803	Spearman's rank correlation rho	two.sided
GALACTUROCAT-PWY: D-galacturonate degradation I	-0.345309205	39363.74734	0.00914763	Spearman's rank correlation rho	two.sided
GLYCOL-GLYOXDEG-PWY: superpathway of glycol metabolism and degradation	-0.269790427	37154.0679	0.044341574	Spearman's rank correlation rho	two.sided
ILEUDEG-PWY: L-isoleucine degradation I	-0.286042264	37629.59665	0.032586536	Spearman's rank correlation rho	two.sided
P122-PWY: heterolactic fermentation	0.265098258	21503.22497	0.048318719	Spearman's rank correlation rho	two.sided
P124-PWY: Bifidobacterium shunt	0.378165877	18194.86644	0.004057459	Spearman's rank correlation rho	two.sided
P161-PWY: acetylene degradation (anaerobic)	0.323022019	19808.37573	0.01517224	Spearman's rank correlation rho	two.sided
P163-PWY: L-lysine fermentation to acetate and butanoate	0.276646517	21165.32291	0.039016508	Spearman's rank correlation rho	two.sided
P221-PWY: octane oxidation	-0.376540579	40277.57733	0.004232261	Spearman's rank correlation rho	two.sided
P461-PWY: hexitol fermentation to lactate, formate, ethanol and acetate	0.288606253	20815.38104	0.030993798	Spearman's rank correlation rho	two.sided
PWY-2941: L-lysine biosynthesis II	0.339501422	19326.1884	0.010472674	Spearman's rank correlation rho	two.sided
PWY-4984: urea cycle	0.272880805	21275.50764	0.04187185	Spearman's rank correlation rho	two.sided
PWY-5130: 2-oxobutanoate degradation I	0.344442003	19181.627	0.009335737	Spearman's rank correlation rho	two.sided
PWY-5690: TCA cycle II (plants and fungi)	-0.463726608	42828.64055	0.000318775	Spearman's rank correlation rho	two.sided
PWY-7379: mRNA capping II	-0.277218198	37371.40447	0.038597427	Spearman's rank correlation rho	two.sided
PWY-7409: phospholipid remodeling (phosphatidylethanolamine, yeast)	-0.289340125	37726.09205	0.030550062	Spearman's rank correlation rho	two.sided
PWY4LZ-257: superpathway of fermentation (Chlamydomonas reinhardtii)	0.303924844	20367.15906	0.022772524	Spearman's rank correlation rho	two.sided
PWY66-398: TCA cycle III (animals)	-0.418495175	41505.16882	0.001328247	Spearman's rank correlation rho	two.sided
PWY6666-2: dopamine degradation	-0.274184602	37282.64147	0.040864403	Spearman's rank correlation rho	two.sided

Supplemental Table 12: Hazard ratios of the incidence of paroxysmal AF in CREM-IbΔC-X mice on MCC950+Choline diet. Hazard ratios (HR) between respective dietary groups were calculated using a Cox proportional hazard model.

Group Comparisons	HR (95% CI)	*P* Value
MCC950+Choline Vs Choline	0.8 (0.4 - 1.5)	0.55

Supplemental Table 13: Hazard ratios of the incidence of persistent AF in CREM-IbΔC-X mice on MCC950+Choline diet

Group Comparisons	HR (95% CI)	*P* Value
MCC950+Choline Vs Choline	2.0 (1.0 - 4.0)	0.03

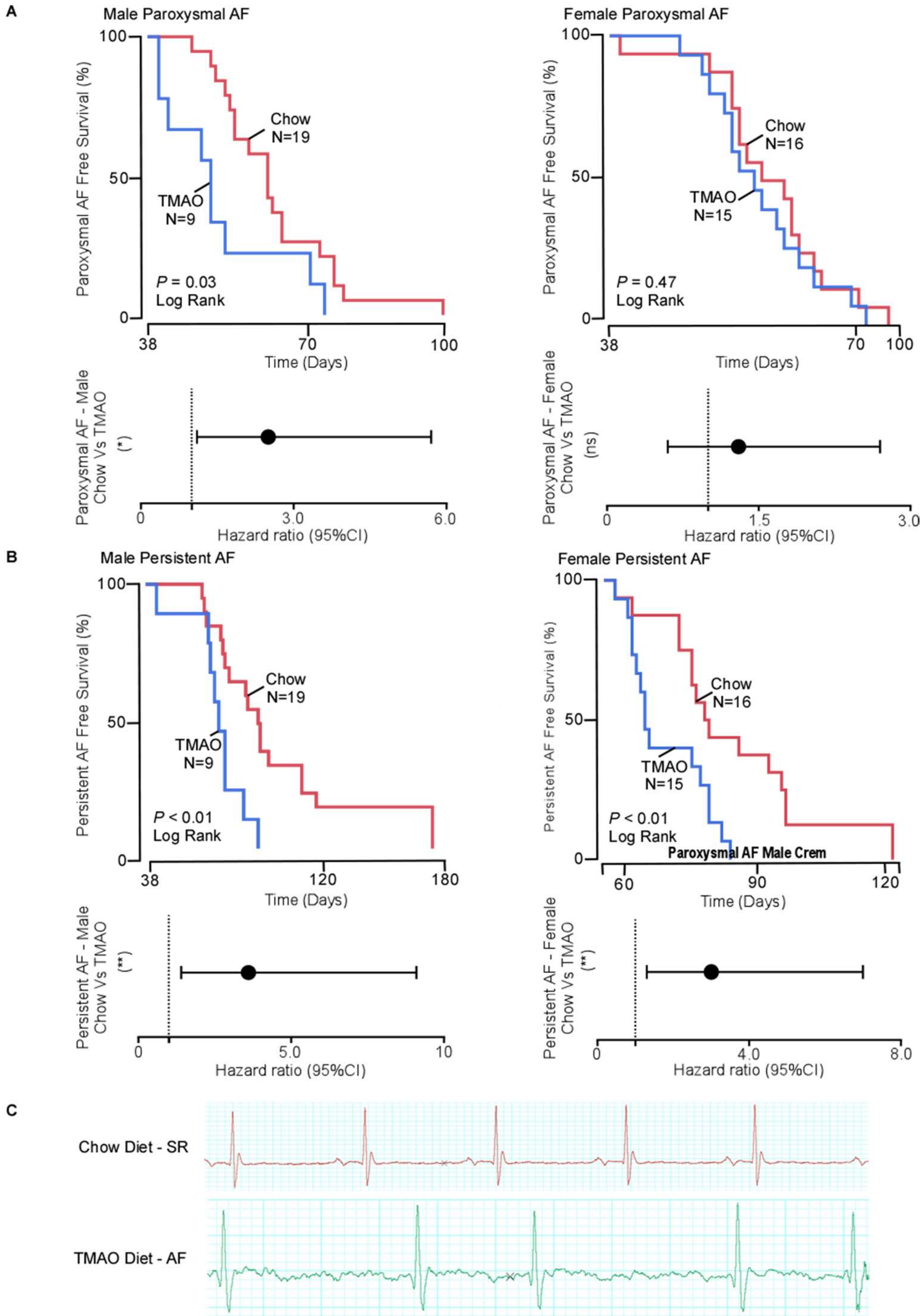

Supplemental Figure 1: Kaplan Meier plots and hazard ratios of the incidence of paroxysmal AF and persistent AF in CREM-IbΔC-X mice on Chow and TMAO supplemented diets. The incidence of (**A**) Paroxysmal AF (**B**) Persistent AF in male and female CREM-IbΔC-X mice represented by a Kaplan Meier Plot (top panel) and Hazard ratios (bottom panel) between respective dietary groups. In A-B, the circles represent the HR and the horizontal bars extend from the lower limit to the upper limit of the 95% CI of the estimate of the HR. HR between respective dietary groups were calculated using a Cox proportional hazard model. (**C**) ECG traces showing characterization of SR (top panel) and AF (bottom panel) in CREM-IbΔC-X mice treated with chow diet and TMAO diet, respectively. AF, atrial fibrillation; CI, confidence interval. Hazard ratios between respective dietary groups were calculated using a Cox proportional hazard model. **P <.05, **P < .01*.

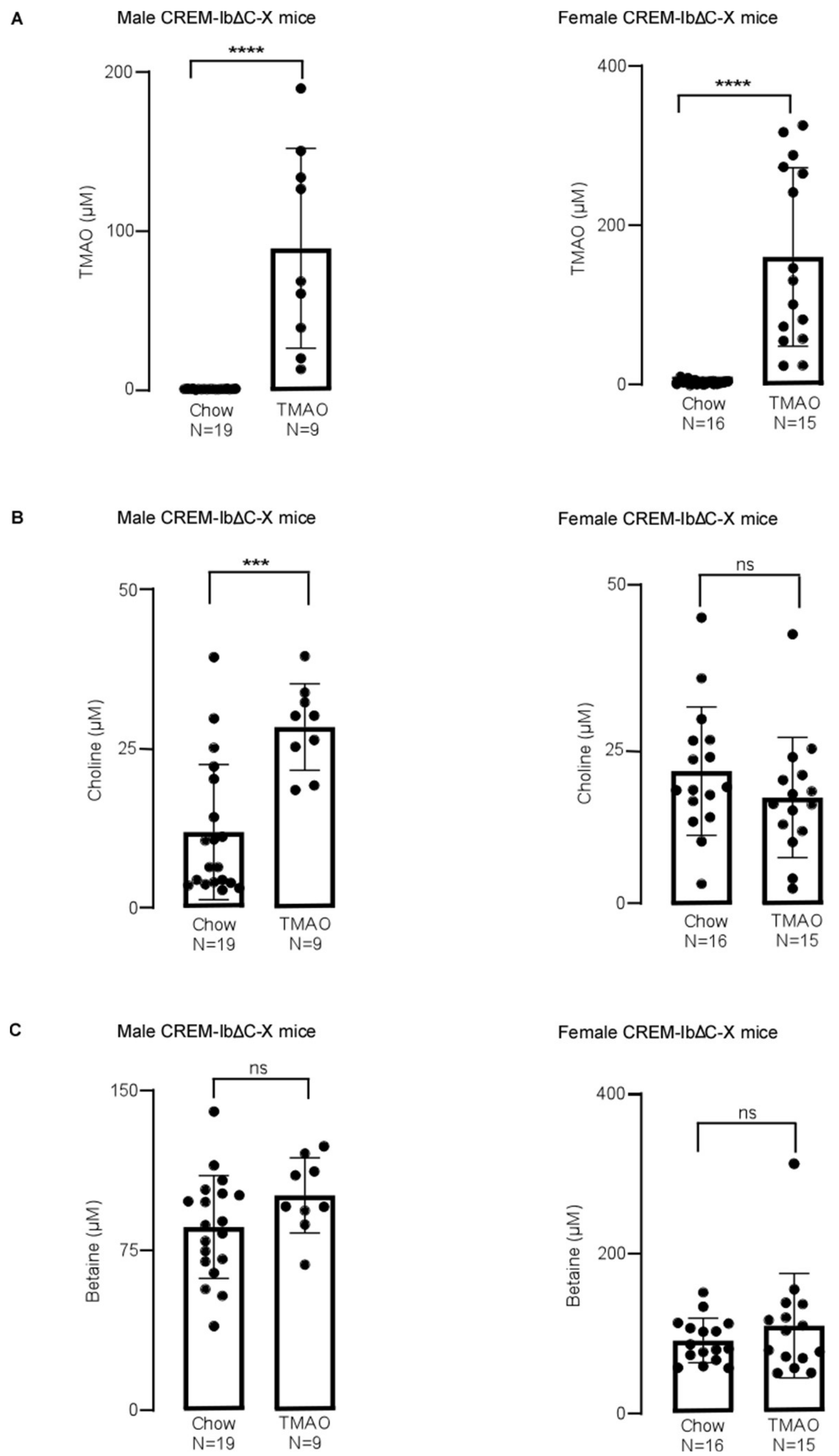

Supplemental Figure 2: Plasma concentrations of TMAs (TMAO, choline, and betaine) in male and female CREM-IbΔC-X mice used in the TMAO ECG study. Each box plot represents the mean ±SD. A Mann Whitney test was used to assess significant differences between groups. ****P < 0.001 ****P < .0001*, ns, non-significant.

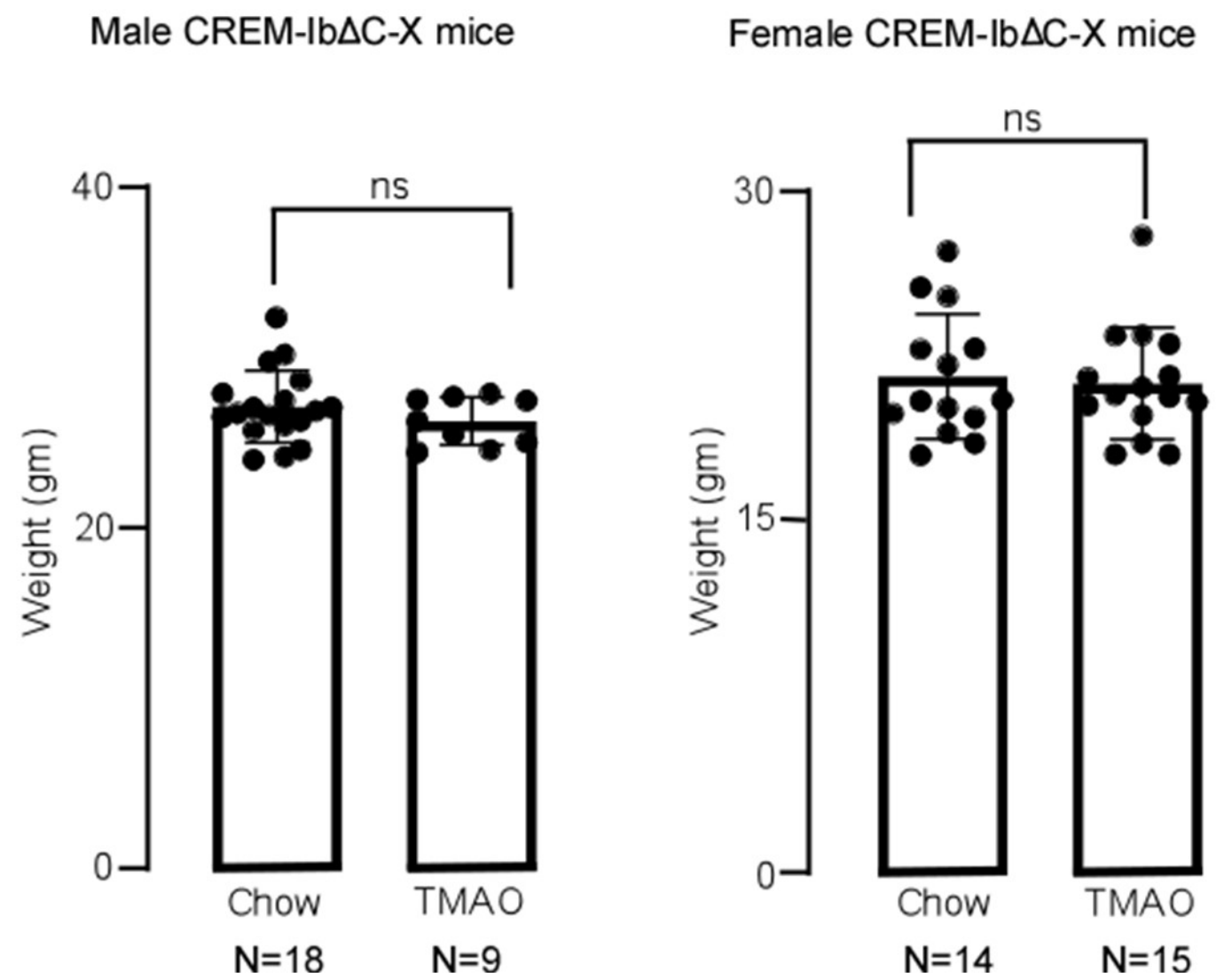

Supplemental Figure 3: CREM-IbΔC-X male and female mouse weight grams (g) used in the TMAO ECG study. Each box plot denotes mean ±SD. A Mann Whitney test was used to assess significant differences between groups. ns, non-significant.

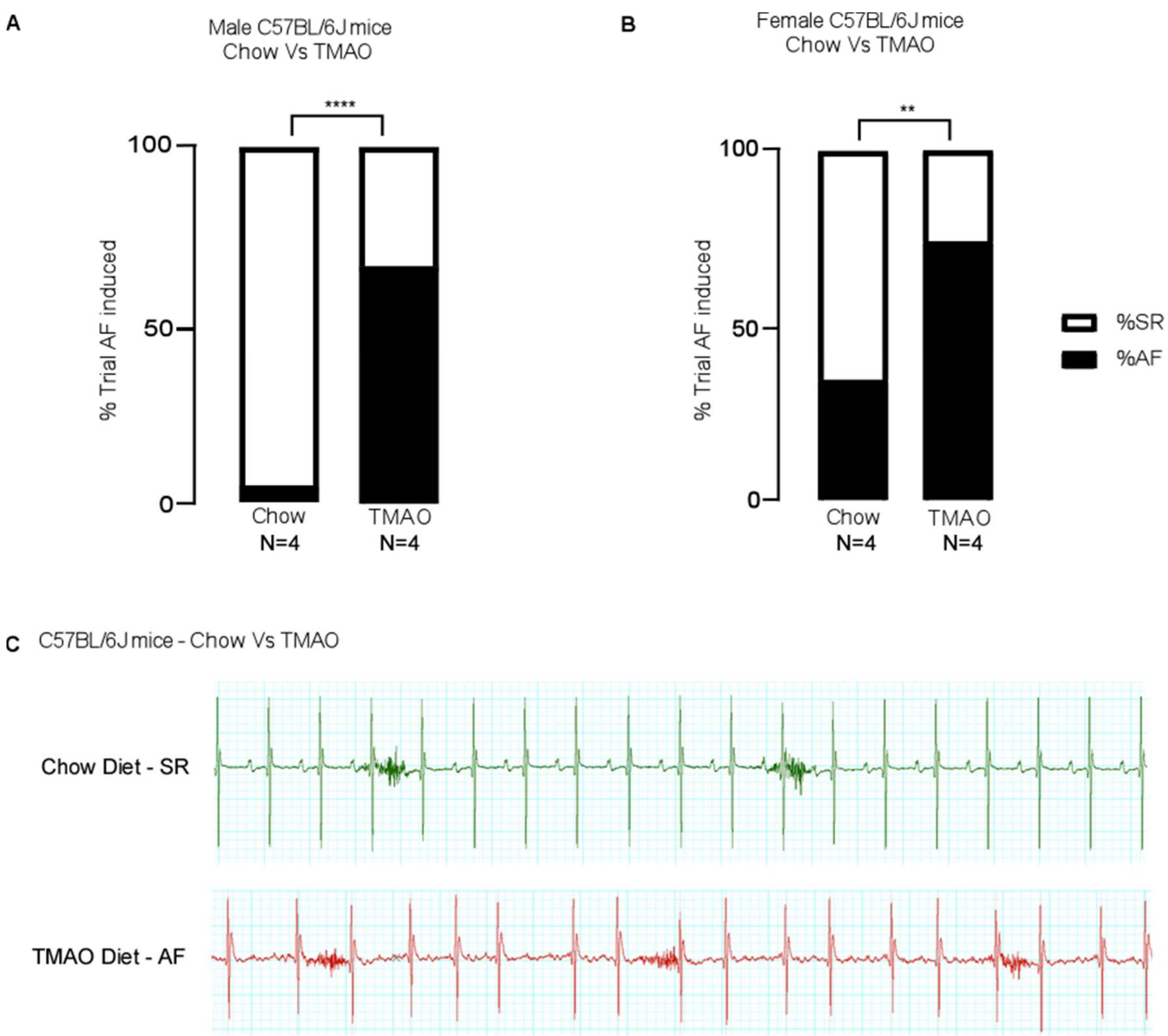

Supplemental Figure 4: Trials of AF (%) in male (A), female (B) C57BL/6J mice treated with TMAO and chow diets. Each box plot represents denotes mean ±SD values. A Chi-square (and Fisher's exact) test was used to assess significant differences between groups. ***P < 0.01, ****P < .0001*. (**C**) ECG traces showing characterization of SR (top panel) and AF (bottom panel) in C57BL/6J mice treated with chow diet and TMAO diet, respectively. AF, atrial fibrillation; SR, Sinus Rhythm.

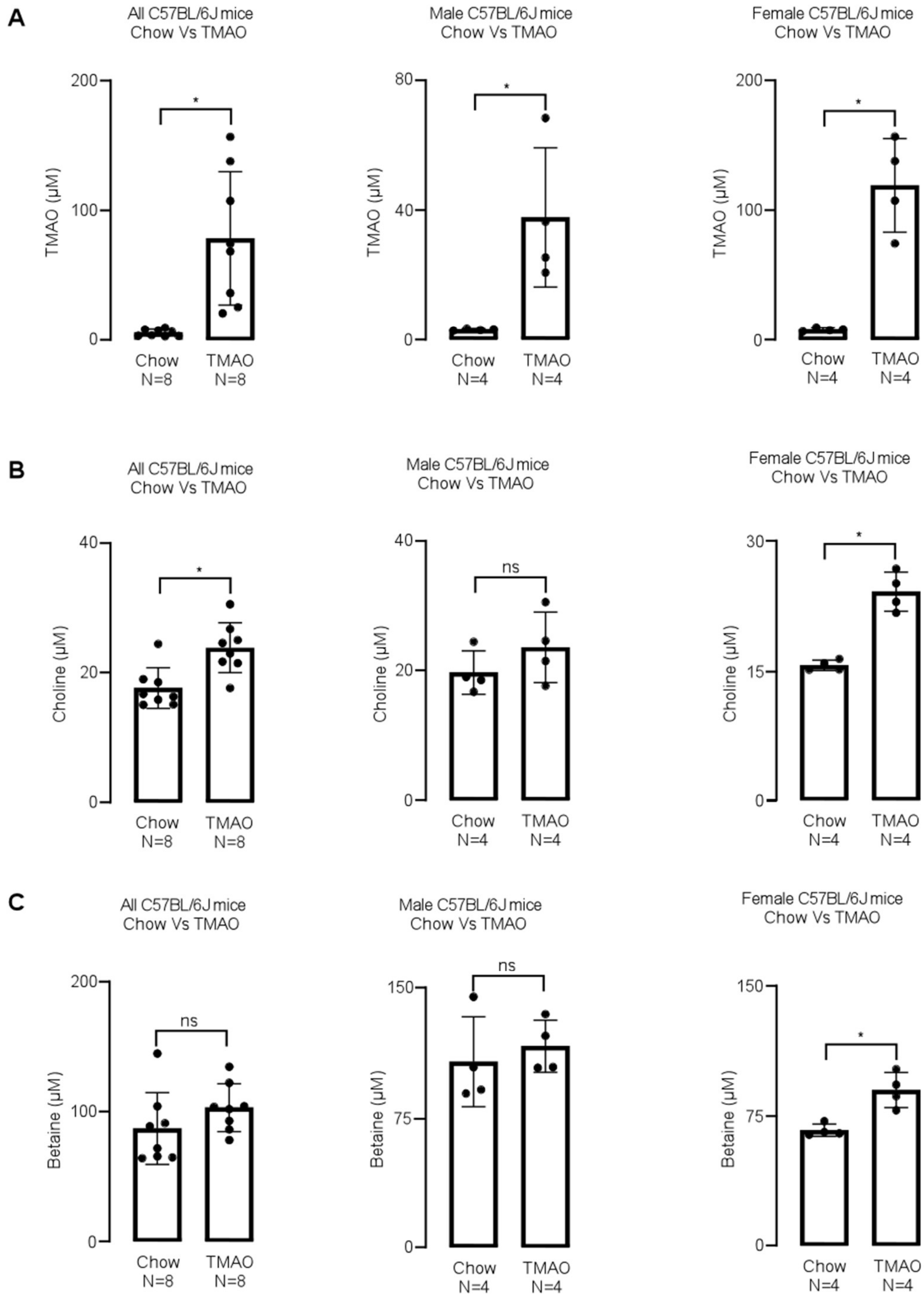

Supplemental Figure 5: Average plasma concentrations of TMAO, choline and betaine in all C57BL/6J mice. Each box plot represents ± SD. A Mann Whitney test was used to assess significant differences between groups. **P <0.05*, ns, non-significant.

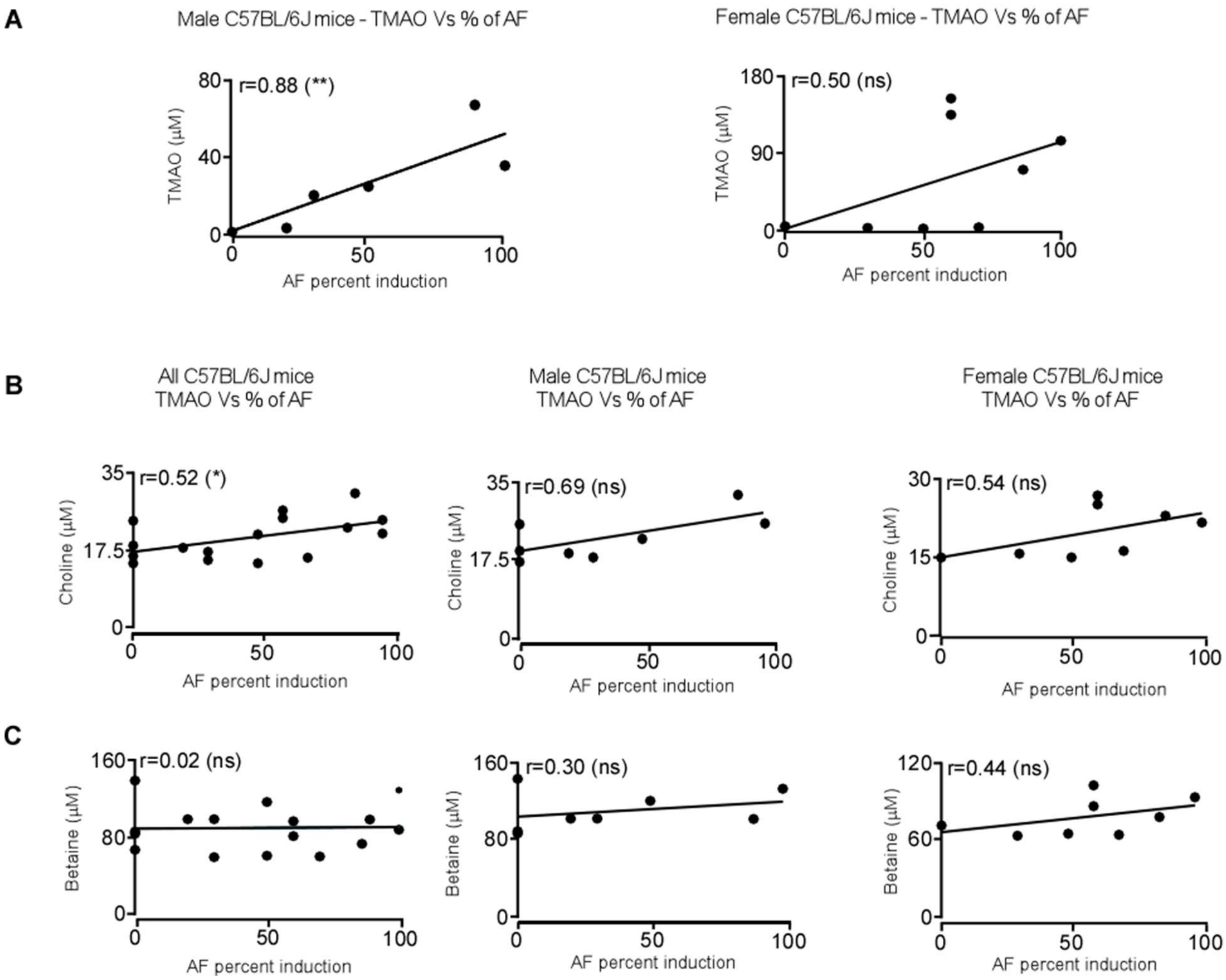

Supplemental Figure 6: TMA levels and AF percent induction correlation in male and femaleC57BL/6J mice. A Pearson correlation coefficient measuring the relationship between TMA levels and AF percent induction. *P* values determined using the linear regression model. **P <0.05, **P < 0.01,* ns, non-significant.

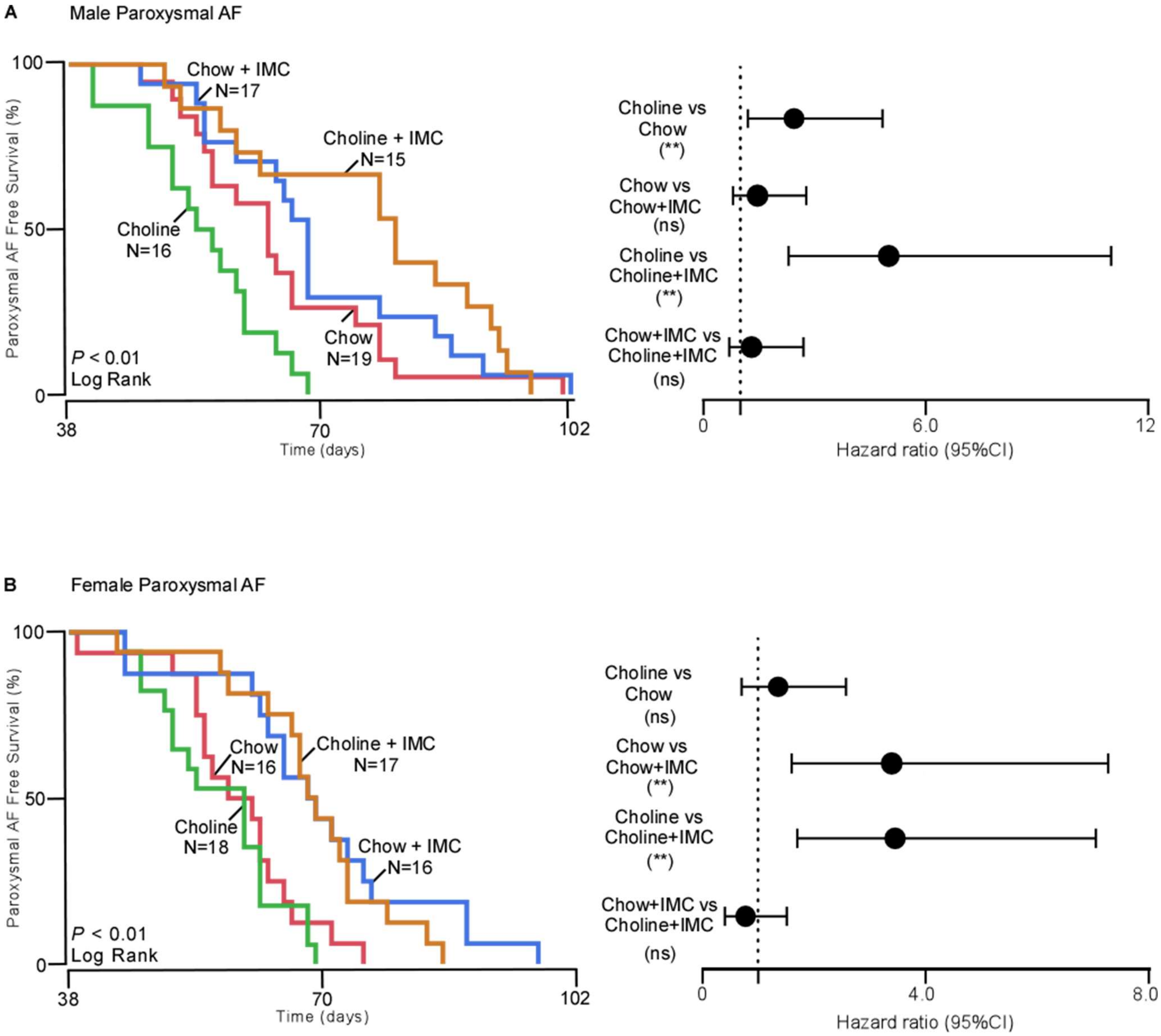

Supplemental Figure 7: The incidence of paroxysmal AF in male (**A**), female (**B**) CREM-IbΔC-X mice represented by a Kaplan Meier Plot (left panel) and Hazard ratios (HRs) (right panel) on chow diet, choline supplemented diet, a chow + IMC supplemented diet, and a choline + IMC supplemented diet. The circles represent the HR and the horizontal bars extend from the lower limit to the upper limit of the 95% CI of the estimate of the HR. HR between respective dietary groups were calculated using a Cox proportional hazard model. AF, atrial fibrillation; IMC, idomethylcholine; CI, confidence interval.. **P <.05, **P < .01*.

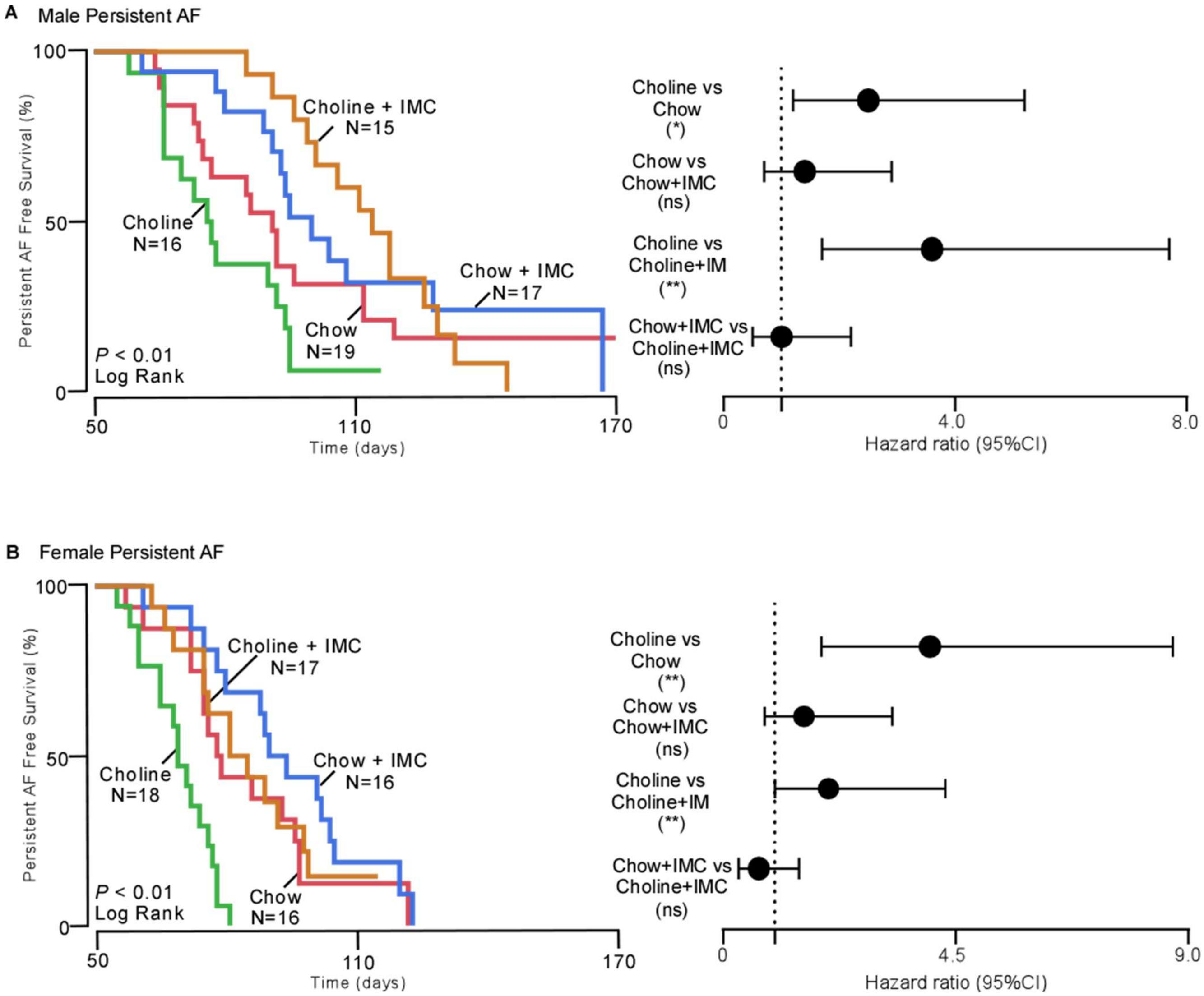

Supplemental Figure 8: The incidence of persistent AF in male (**A**), female (**B**) CREM-IbΔC-X mice represented by a Kaplan Meier Plot (left panel) and Hazard ratios (HRs) (right panel) on chow diet, choline supplemented diet, a chow + IMC supplemented diet, and a choline + IMC supplemented diet. The circles represent the HR and the horizontal bars extend from the lower limit to the upper limit of the 95% CI of the estimate of the HR. HR between respective dietary groups were calculated using a Cox proportional hazard model. AF, atrial fibrillation; IMC, idomethylcholine; CI, confidence interval.. **P <.05, **P < .01*.

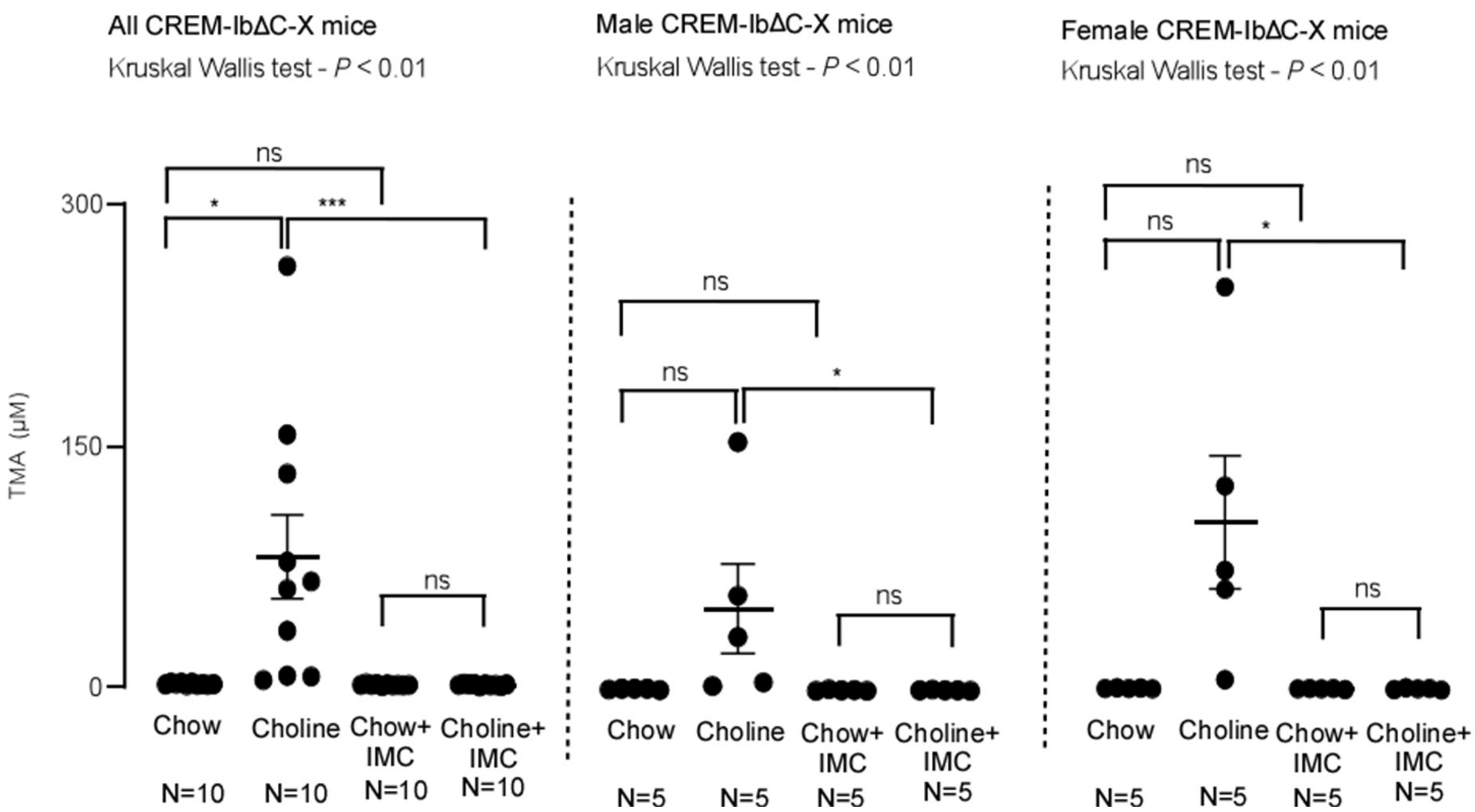

Supplemental Figure 9: TMA plasma concentration was quantified in mouse plasma in a subset of CREM-IbΔC-X mice used in the mouse ECG experiment. The line denotes mean ±SD values. A Kruskal-Wallis test with Dunn’s post hoc analysis was used to determine the significant difference between the medians of four independent diet groups and multiple paired comparison adjustments were performed. *$^{*}P < 0.05$, $^{***}P < 0.001$*, ns, non-significant.

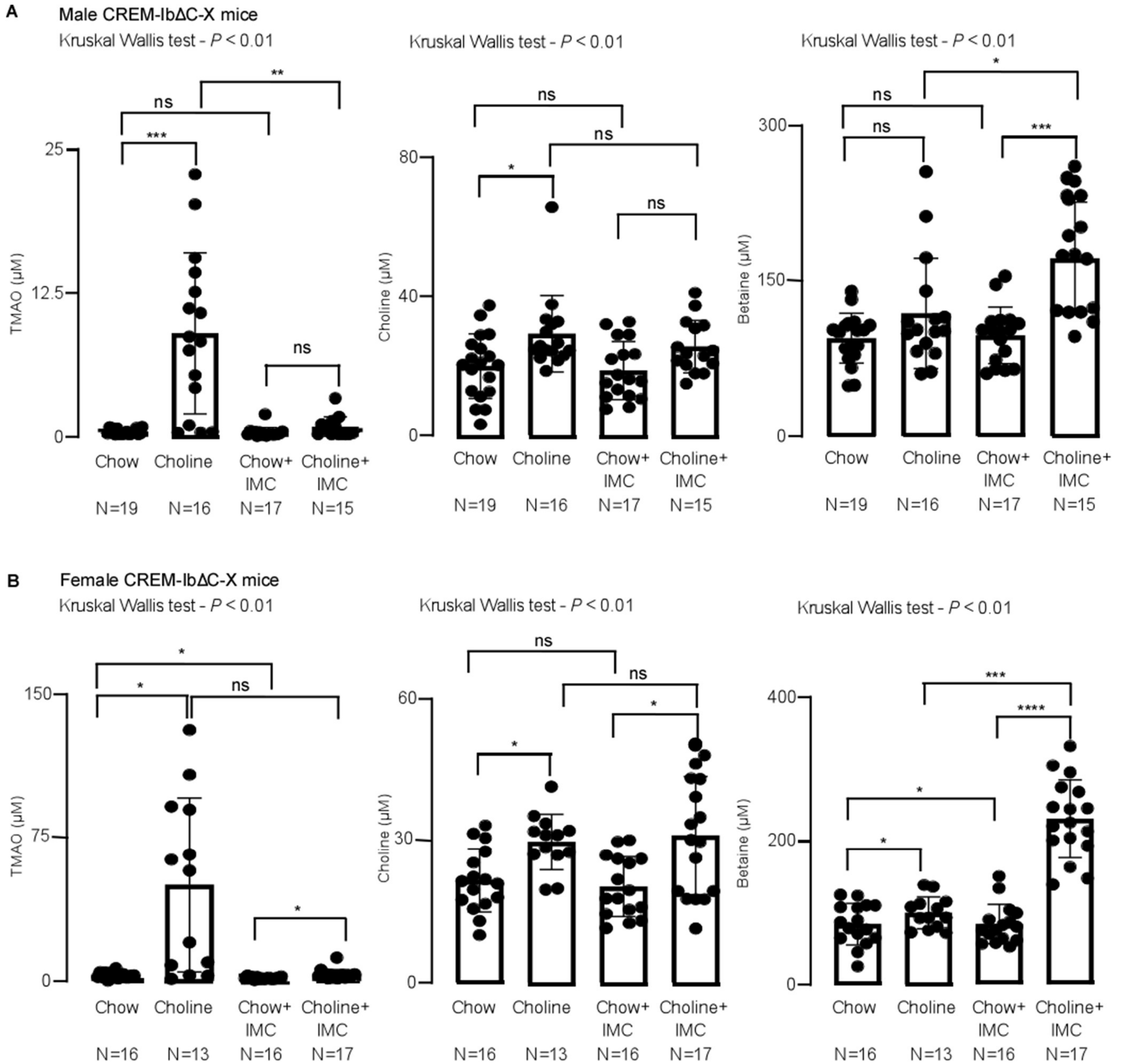

Supplemental Figure 10: Average plasma concentrations of TMAO, choline and betaine in male (**A**), female (**B**) CREM-IbΔC-X mice on respective diets used in ECG study. Each box plot represents mean ± SD. A Kruskal-Wallis test with Dunn's post hoc analysis was used to determine the significant difference between the medians of four independent diet groups and multiple paired comparison adjustments were performed. **P <0.05, **P < 0.01, ***P < 0.001, ****P <0 .0001*, ns, non-significant.

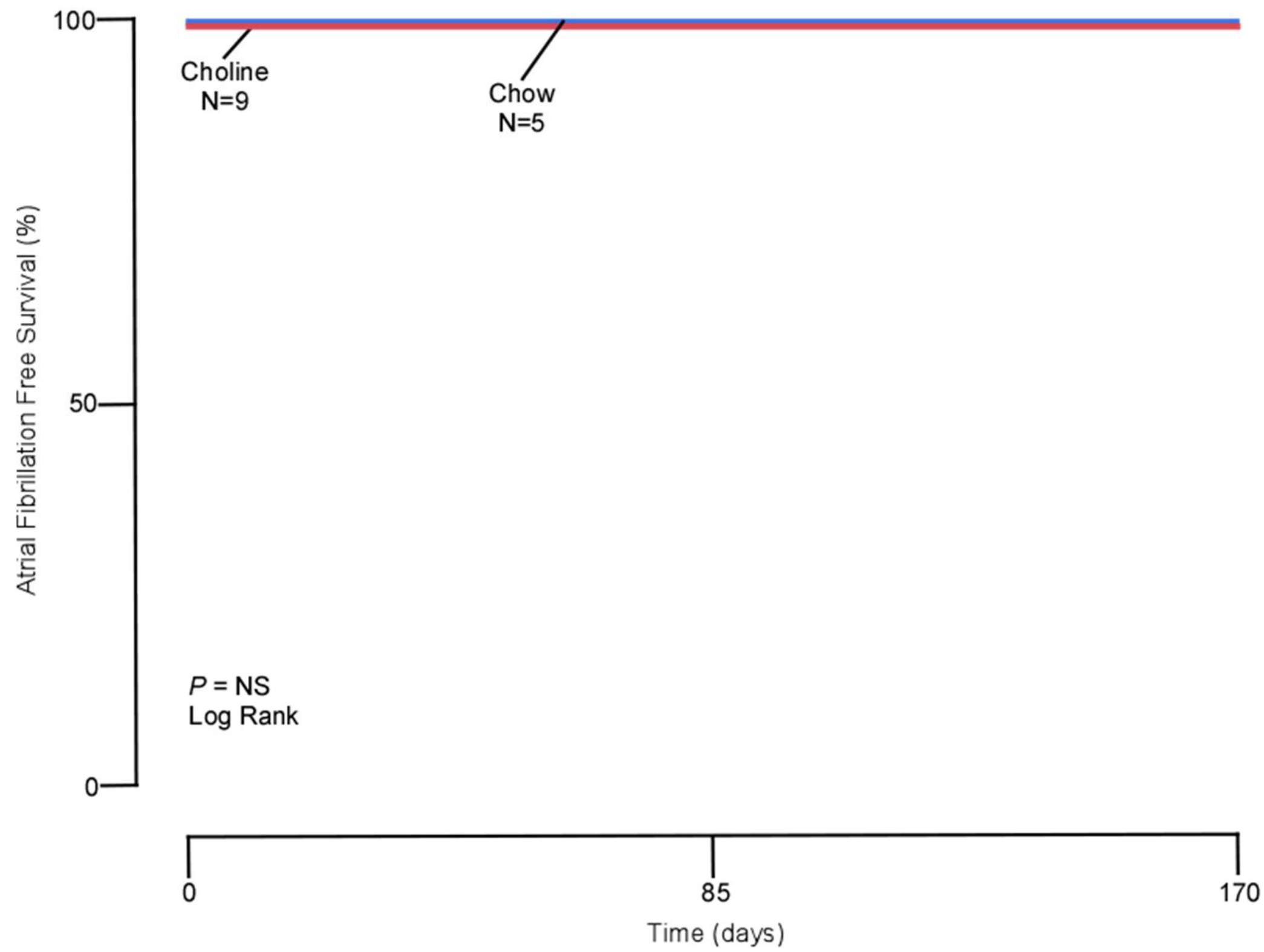

Supplemental Figure 11: Kaplan Meier plot of the incidence of atrial fibrillation on WT mice on a Chow diet (N=5 (n=2 Female, n=3 Male)) and Choline 1.3% supplemented diet [N = 9 (n = 4 Female, n = 5 Male)]. A Mann Whitney test was used to assess significant differences between groups. ns, non-significant.

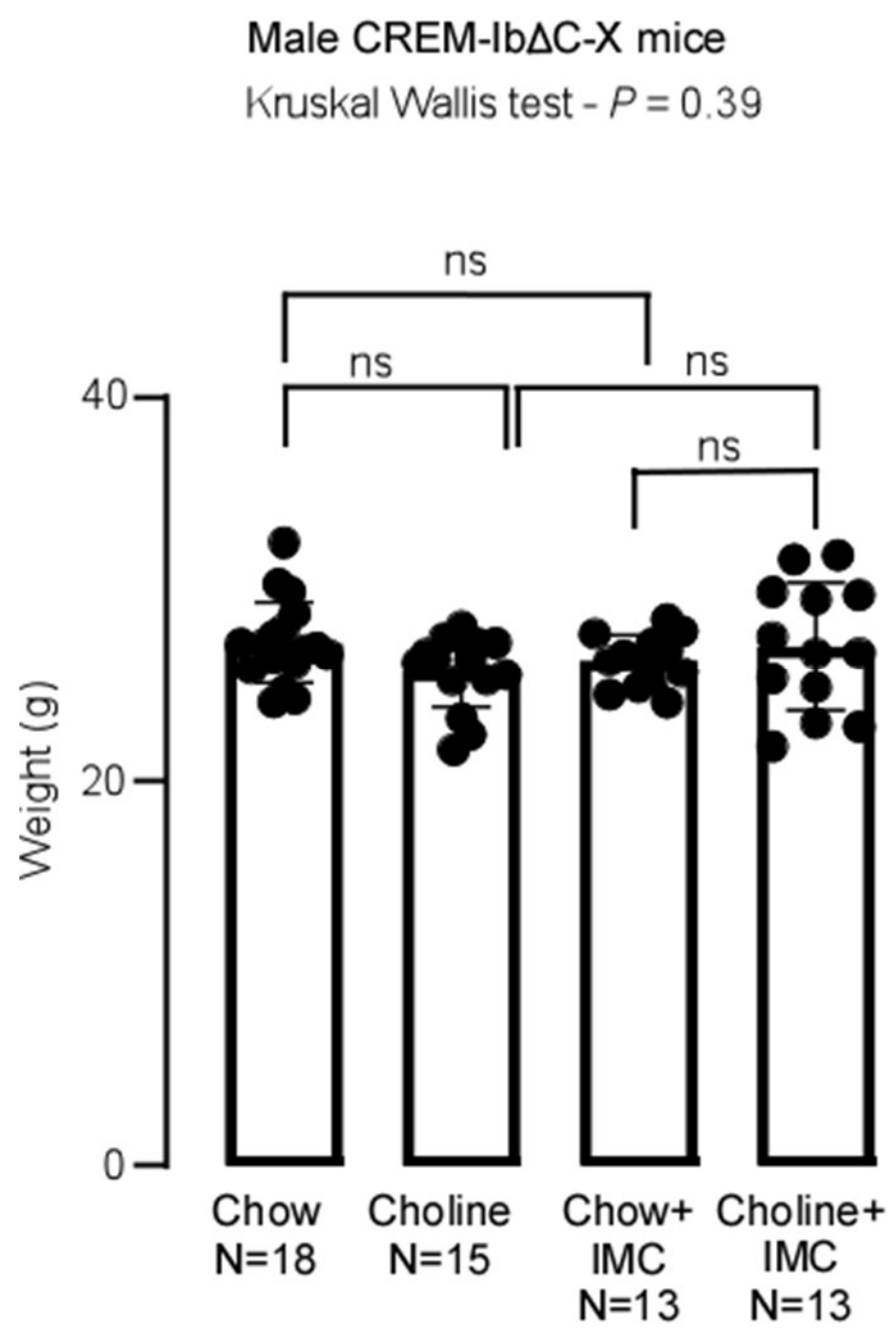

Supplemental Figure 12: Terminal mouse weight of male and female CREM-IbΔC-X mice on respective diets used in the mouse ECG experiments. Each box plot represents mean ±SD. A Kruskal-Wallis test with Dunn's post hoc analysis was used to determine the significant difference between the medians of four independent diet groups and multiple paired comparison adjustments were performed. ns, non-significant.

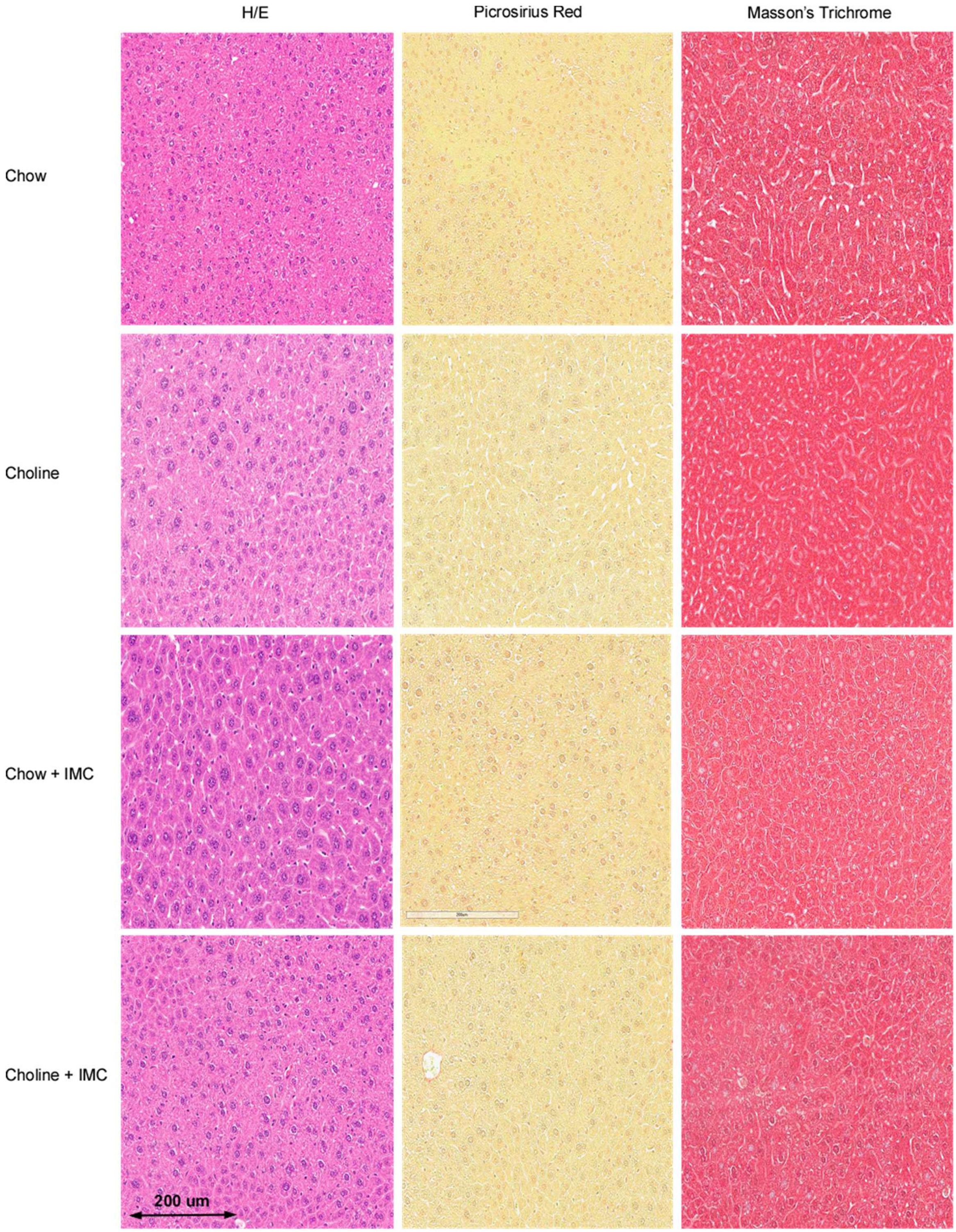

Supplemental Figure 13: Hematoxylin/eosin (H/E), Picrosirius Red, and Masson's Trichrome stained liver sections. Representative liver sections of CREM-IbΔC-X mice used in the serial ECG study at the time. No evident structural or fibrotic changes in liver were noted between CREM-IbΔC-X mice supplemented with choline or respective controls (Chow+IMC and Choline+IMC diet) when compared to Chow CREM-IbΔC-X mice.

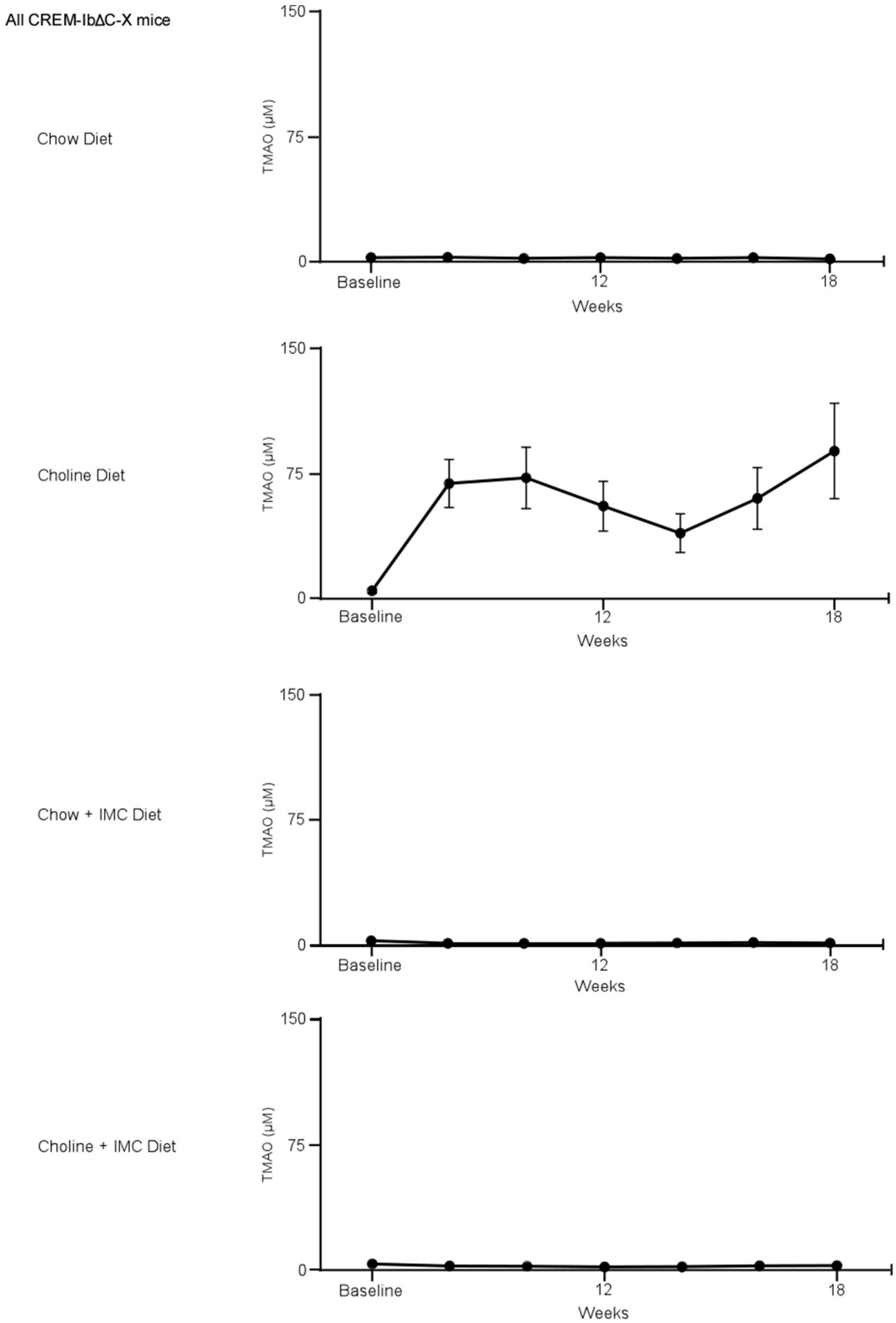

Supplemental Figure 14: Serial average plasma concentrations of TMAO in both male and female CREM-IbΔC-X mice fed on chow diet, choline supplemented diet, a chow + IMC supplemented diet, and a choline + IMC supplemented diet used in ECG study. Baseline refers to CREM-IbΔC-X mice at 4 weeks of age. The line denotes mean ± SEM.

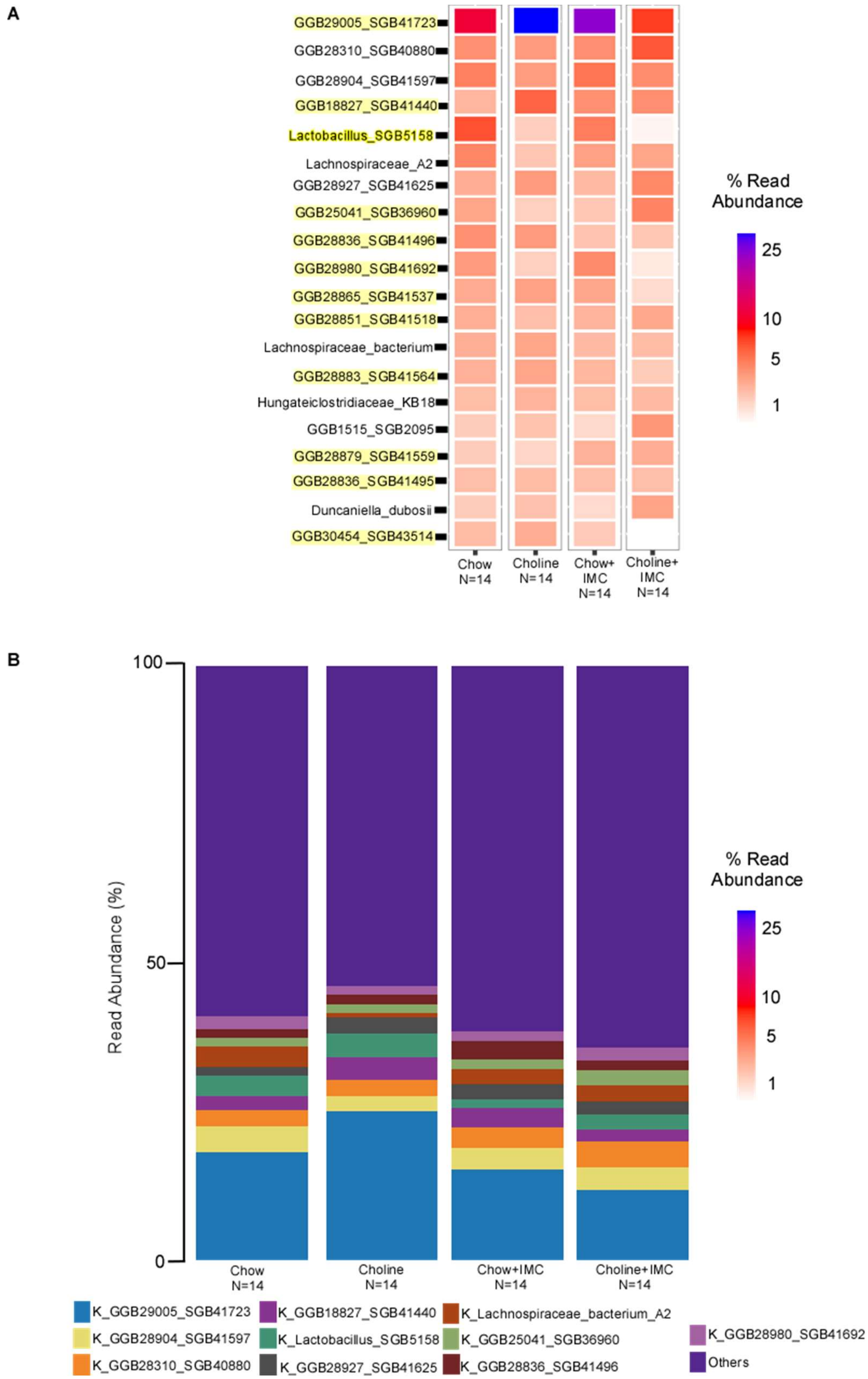

Supplemental Figure 15: (**A**) A heat plot demonstrating the relative read abundance of gut microbiota taxa significantly associated with CREM-IbΔC-X mouse diets (Microbiome Firmicutes are highlighted in yellow). (**B**) A stacked bar plot of the top 10 most abundant species demonstrating the relative read abundance of significant gut microbiota taxa stratified by respective diet.

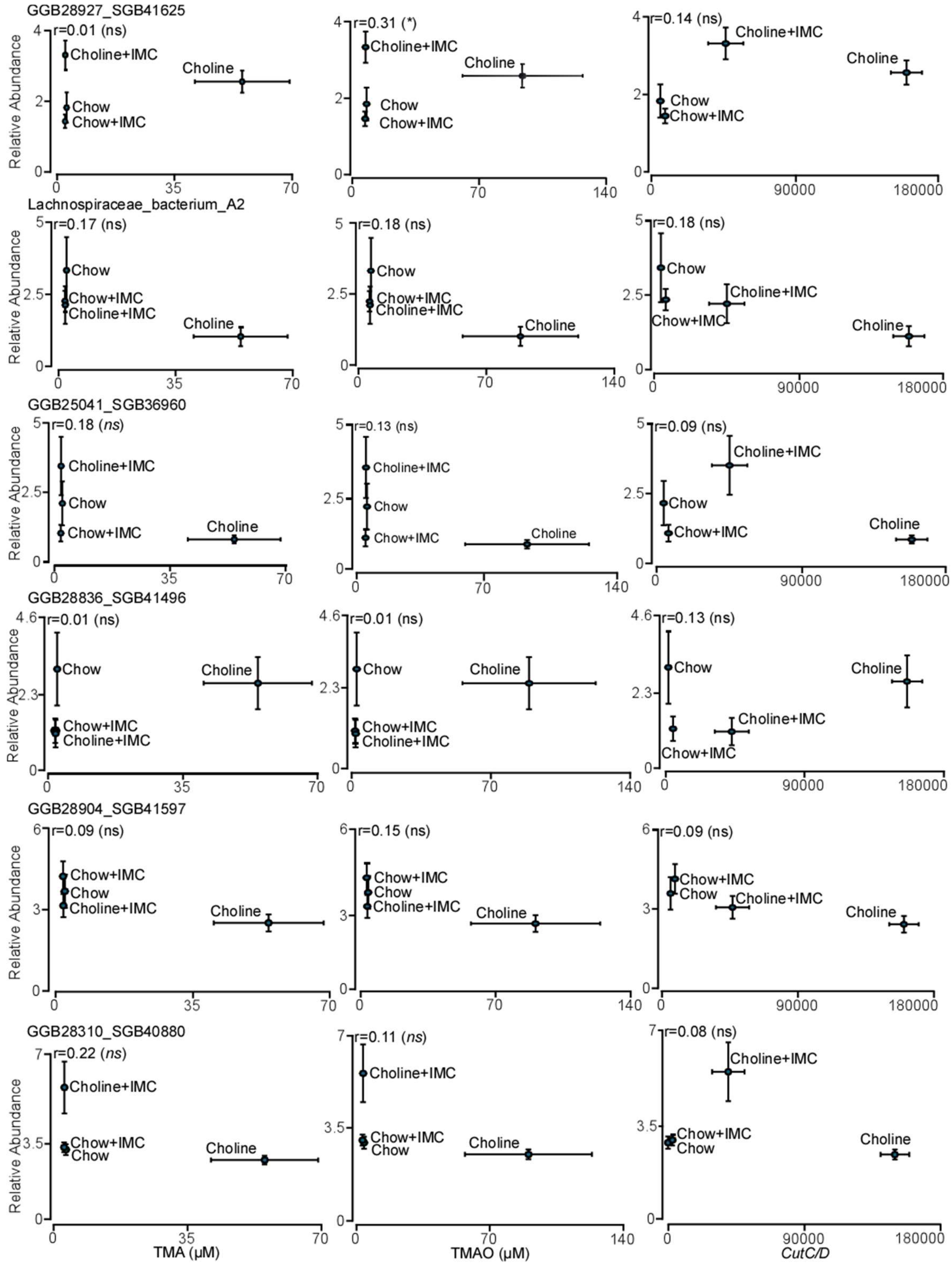

Supplemental Figure 16: Gut microbiota taxa abundance and its association with mouse plasma TMA and TMAO and *CutC/D* counts in linked cecal samples of male and female CREM-IbΔC-X mice on respective diets. The circles represent the mean, and the bars represent the standard errors with regression. *P* values were determined using the linear regression model. **P <0.05*, ns, non-significant.

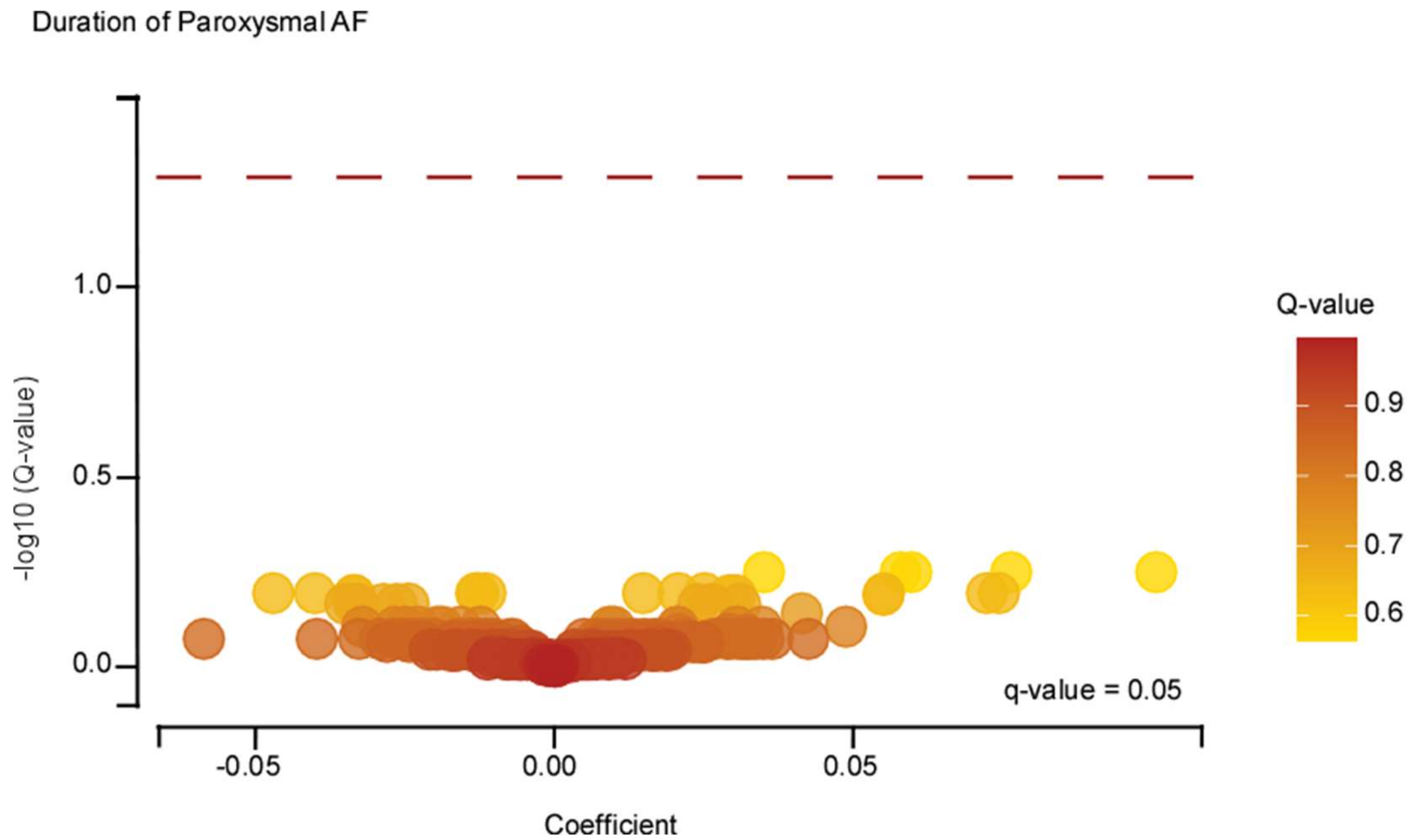

Supplemental Figure 17: The volcano plot showing no significant association of the differential abundance of gut microbiome with the duration of paroxysmal AF. In the plot, the x-axis represents the coefficient (effect size and direction) and the y-axis showing the -log10(Q-value) for statistical significance. Each circle represents a specific microbial species, colored on a gold-to-brickred gradient indicating q-values, with gold being more significant. The dashed dark red line at y≈1.30 marks the significance threshold (q-value=0.05). The Benjamini-Hochberg false discovery rate correction was used to adjust q-values for multiple testing.

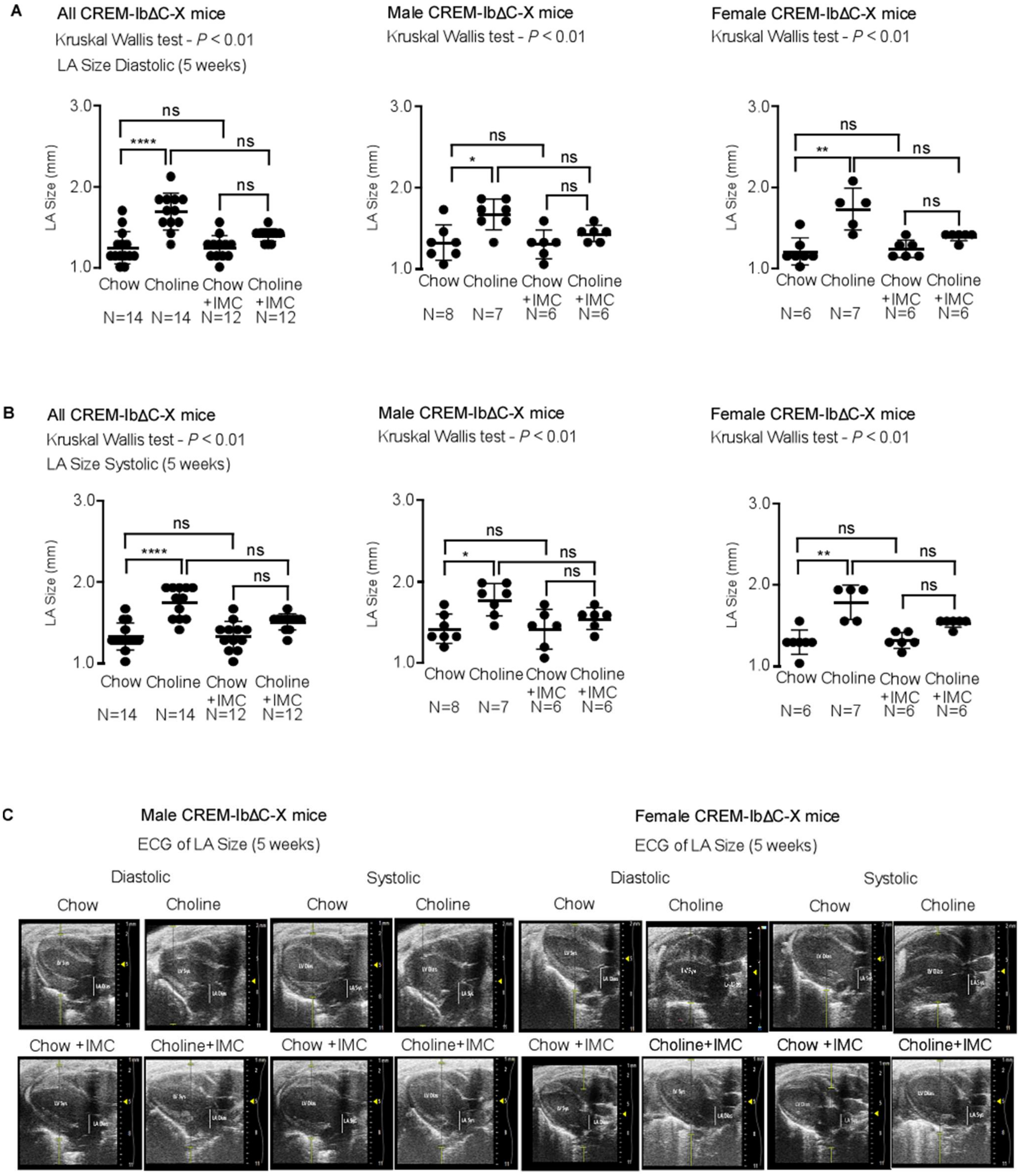

Supplemental Figure 18: Choline diet increases the left atrium (LA) size during atrial diastole and systole. (**A**) LA diastolic in size, male and female CREM-IbΔC-X mice at 5 weeks of age on respective diets. (**B**) LA systolic size in male and female CREM-IbΔC-X mice at 5 weeks of age on respective diets. (**C**) Echocardiography (ECHO) of LA size in female and male CREM-IbΔC-X mice at 5 weeks of age at respective diets. Each line denotes mean ± SD. A Kruskal-Wallis test with Dunn's post hoc analysis was used to determine the significant difference between the medians of four independent diet groups and multiple paired comparison adjustments were performed. **P <0.05, **P < 0.01, ****P < .00001*, ns, non-significant.

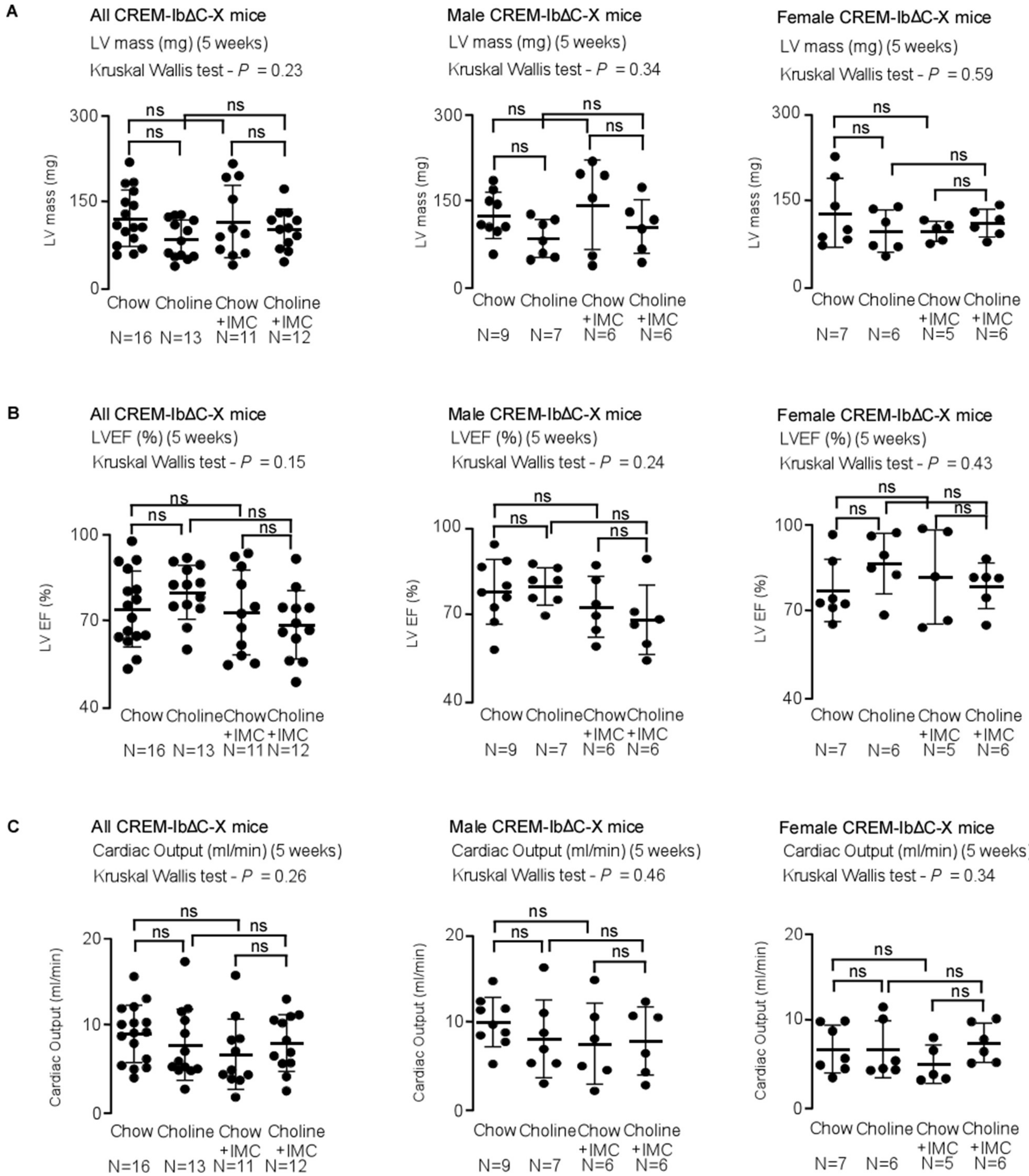

Supplemental Figure 19: LV mass, LVEF, cardiac output between diet groups. (**A**) LV mass, (**B**) LVEF, (**C**) Cardiac ouput in all (left panels), male (mid paneles), and female (right panels) CREM-IbΔC-X mice at 5 weeks of age on respective diets. Each line denotes mean ± SD. A Kruskal-Wallis test with Dunn's post hoc analysis was used to determine the significant difference between the medians of four independent diet groups and multiple paired comparison adjustments were performed. ns, non-significant.

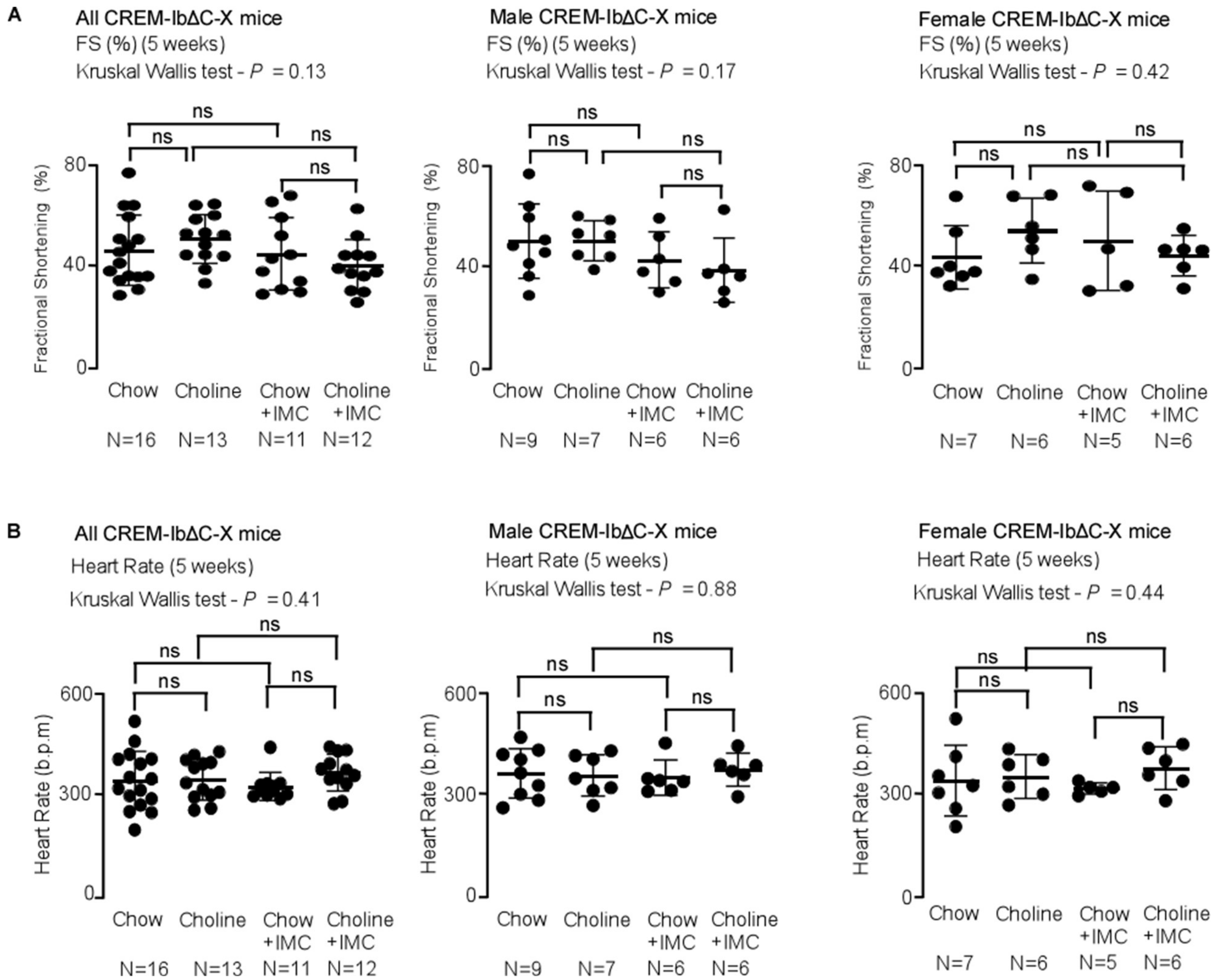

Supplemental Figure 20: Changes in fractional shortening and heart rate between diet groups. (**A**) Fractional shortening mass and (**B**) Heart rate, in all (left panels), male (mid panels), and female (left panels) CREM-IbΔC-X mice at 5 weeks of age on respective diets. Each line denotes mean ± SD. A Kruskal-Wallis test with Dunn's post hoc analysis was used to determine the significant difference between the medians of four independent diet groups and multiple paired comparison adjustments were performed. ns, non-significant.

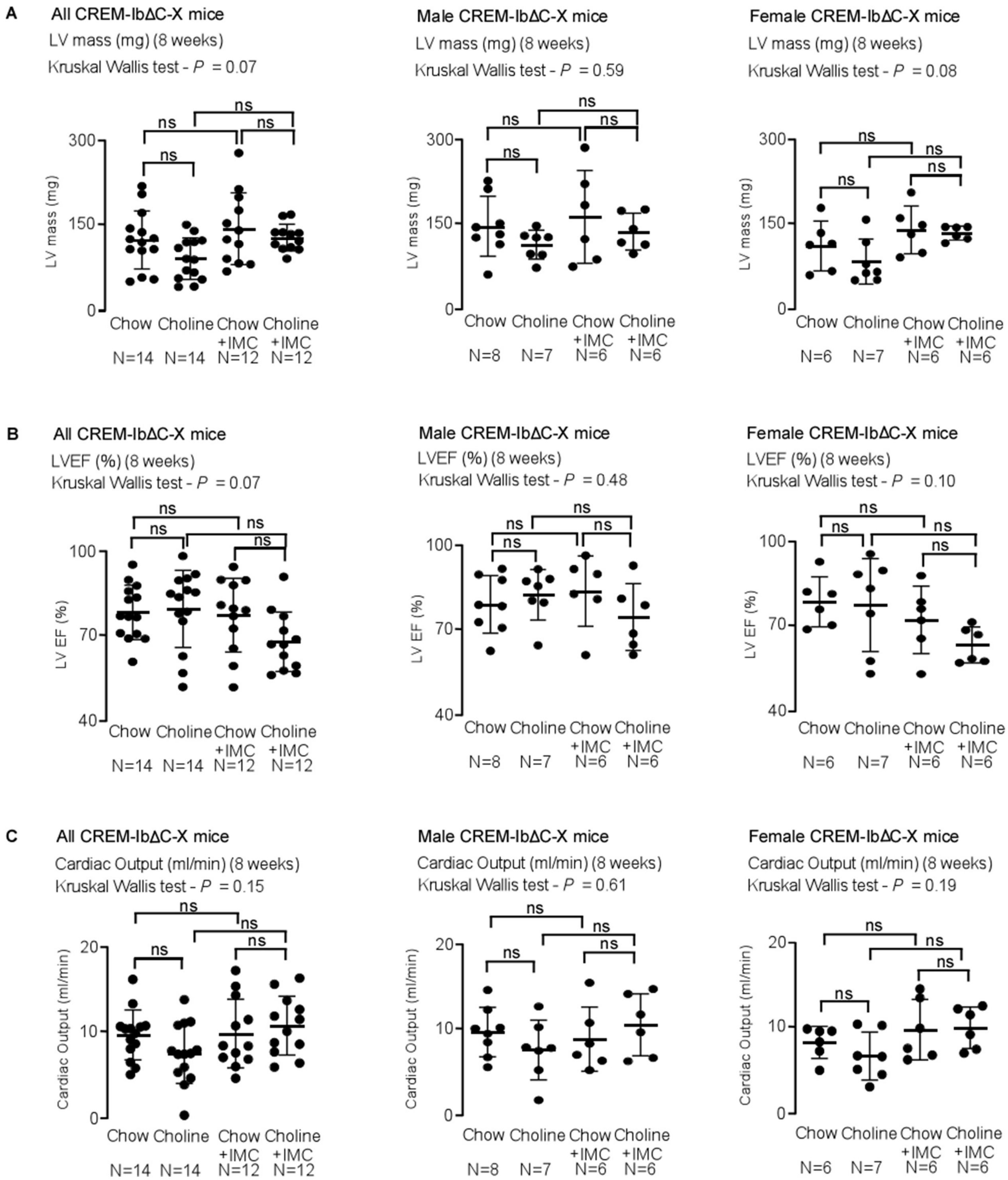

Supplemental Figure 21: LV mass, LVEF, cardiac output between diet groups. (**A**) LV mass, (**B**) LVEF, (**C**) Cardiac ouput in all (left panels), male (mid paneles), and female (right panels) CREM-IbΔC-X mice at 8 weeks of age on respective diets. Each line denotes mean ± SD. A Kruskal-Wallis test with Dunn's post hoc analysis was used to determine the significant difference between the medians of four independent diet groups and multiple paired comparison adjustments were performed. ns, non-significant.

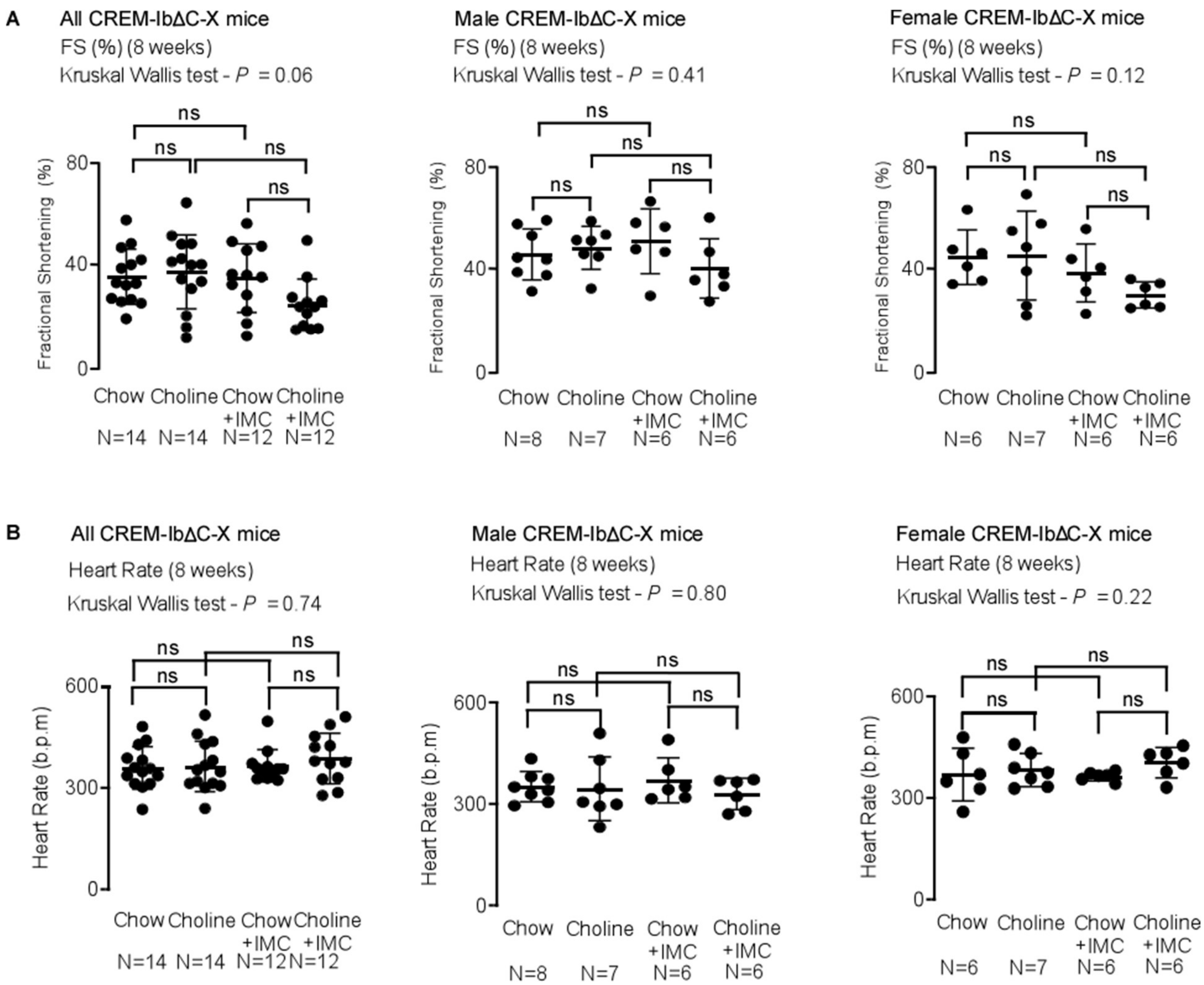

Supplemental Figure 22: Changes in fractional shortening and heart rate between diet groups. (**A**) Fractional shortening mass and (**B**) Heart rate, in all (left panels), male (mid panels), and female (left panels) CREM-IbΔC-X mice at 8 weeks of age on respective diets. Each line denotes mean ± SD. A Kruskal-Wallis test with Dunn's post hoc analysis was used to determine the significant difference between the medians of four independent diet groups and multiple paired comparison adjustments were performed. ns, non-significant.

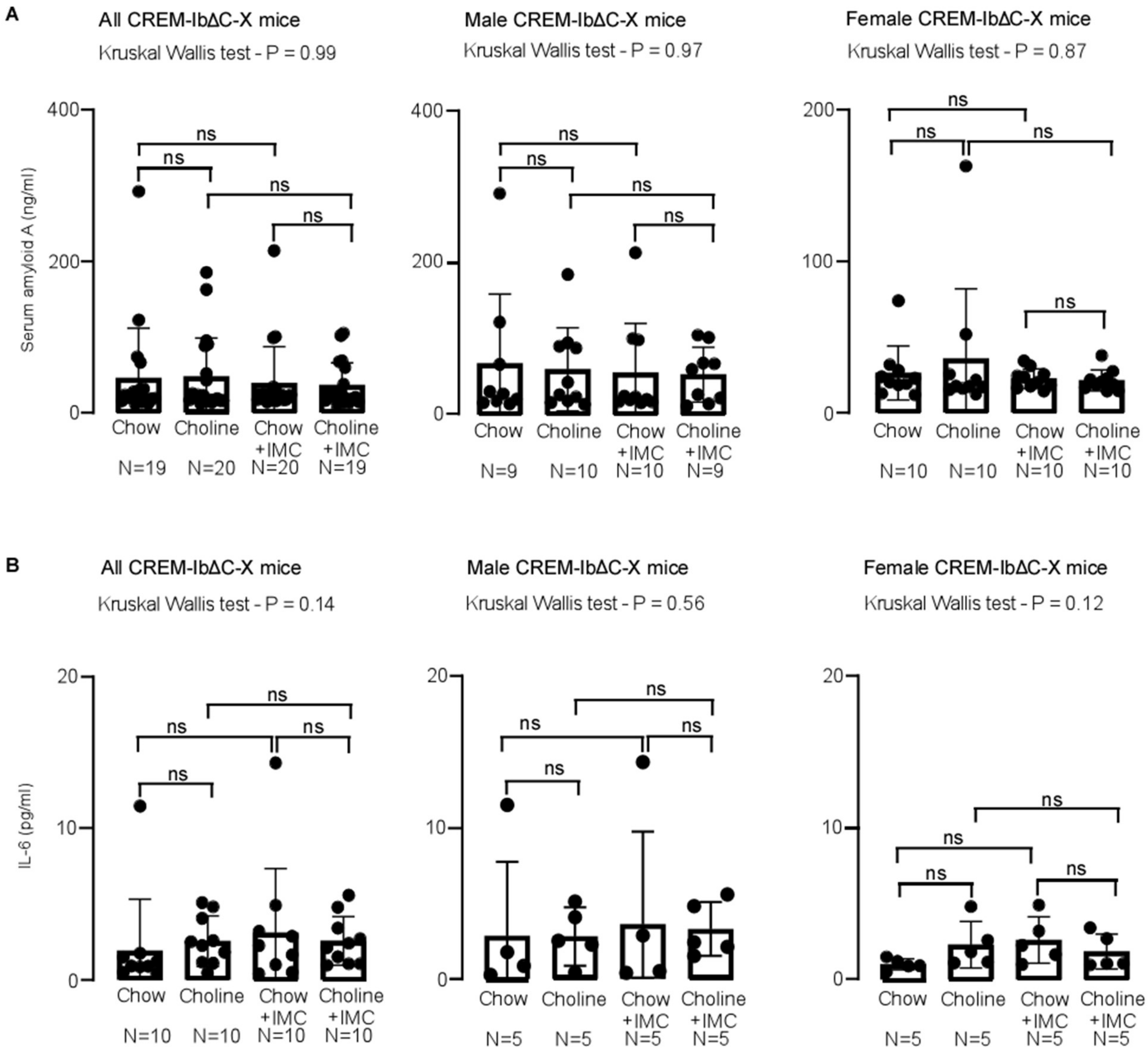

Supplemental Figure 23: (A) Serum amyloid A **(B)** IL-6 plasma levels among the different diet and tretment groups. Each box plot represents mean ± SD. A Kruskal-Wallis test with Dunn's post hoc analysis was used to determine the significant difference between the medians of four independent diet groups and multiple paired comparison adjustments were performed. ns, non-significant.

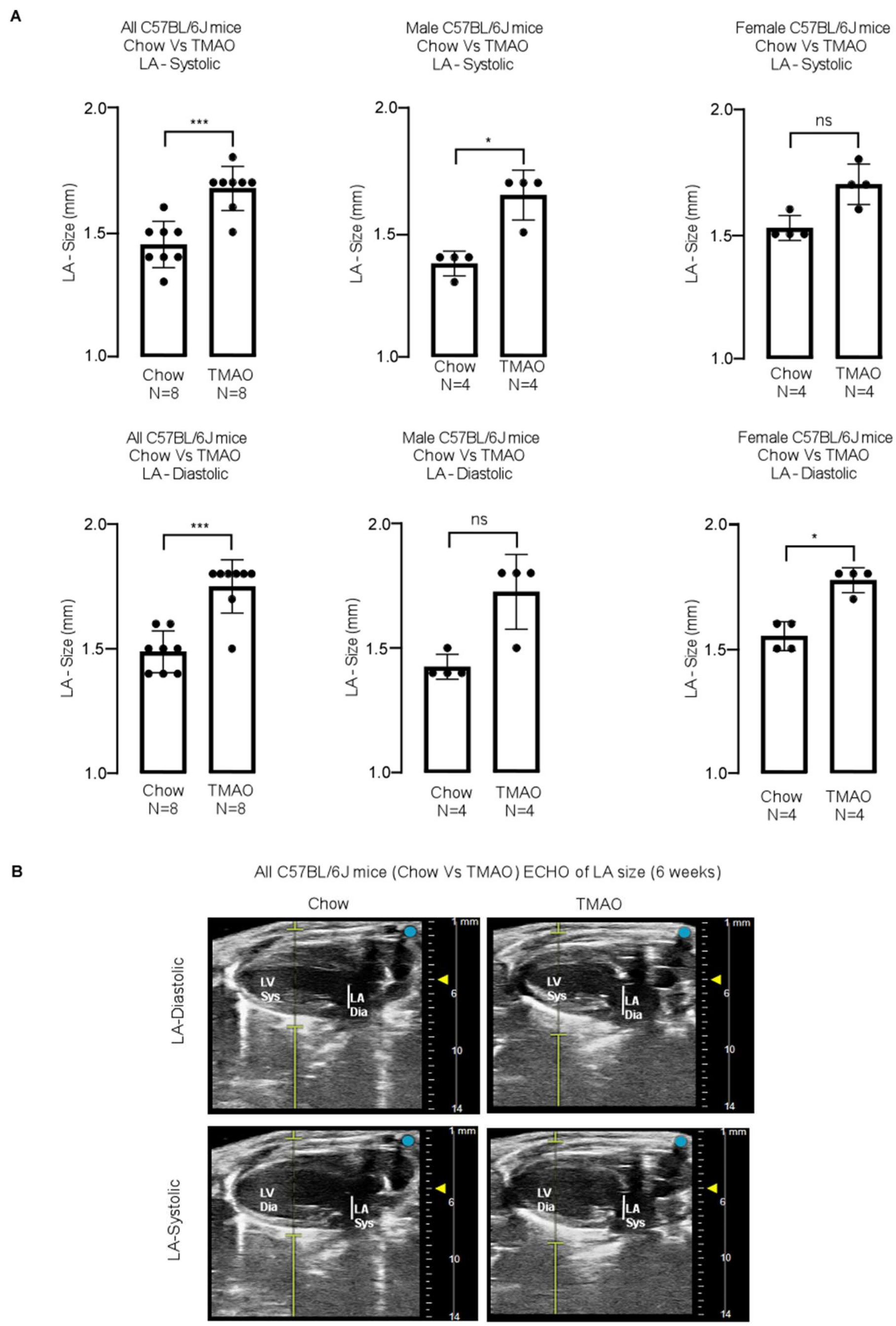

Supplemental Figure 24: TMAO supplementation increases the left atrium (LA) size during the atrial systole and diastole in C57BL/6J mice. (A) LA systolic and diastolic in size, in all C57BL/6J mice at 6 weeks of age on respective diets. (**B**) Echocardiography (ECHO) of LA size in all C57BL/6J mice at 6 weeks of age at respective diets. Each box plot represents mean ±SD. A Mann Whitney test was used to assess significant differences between groups. **P <0.05, ***P < 0.001,* ns, non-significant.

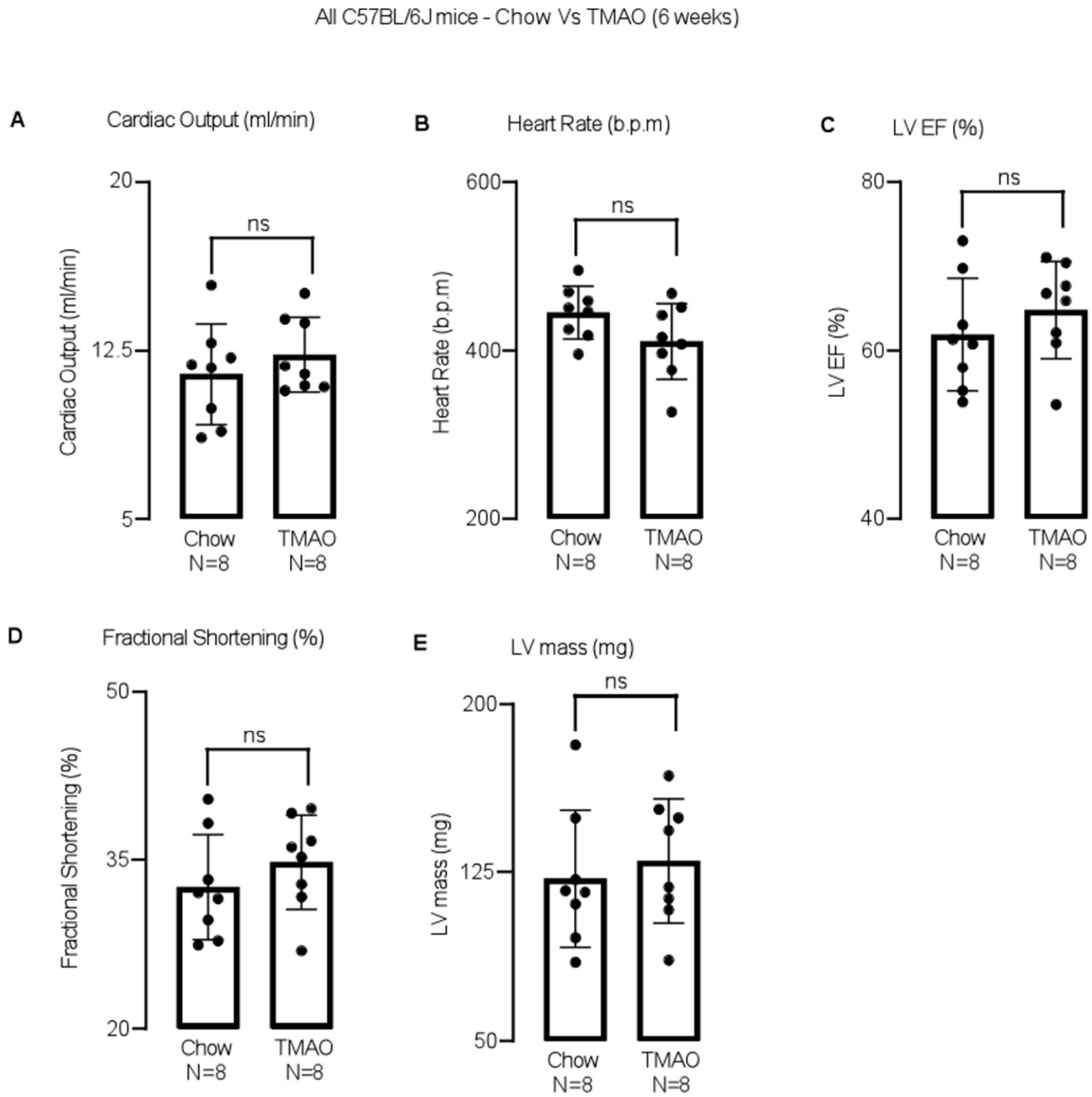

Supplemental Figure 25: LV mass, LVEF, cardiac output between TMAO and chow groups in C57BL/6J mice used in transesophageal pacing AF studies. (**A**) Cardiac ouput, (**B**) Heart rate, (**C**) LVEF, (**D**) Frrational shortening, (**E**) LV mass in C57BL/6J mice at 6 weeks of age on respective diets Each box plot represents mean ±SD. A Mann Whitney test was used to assess significant differences between groups. ns, non-significant.

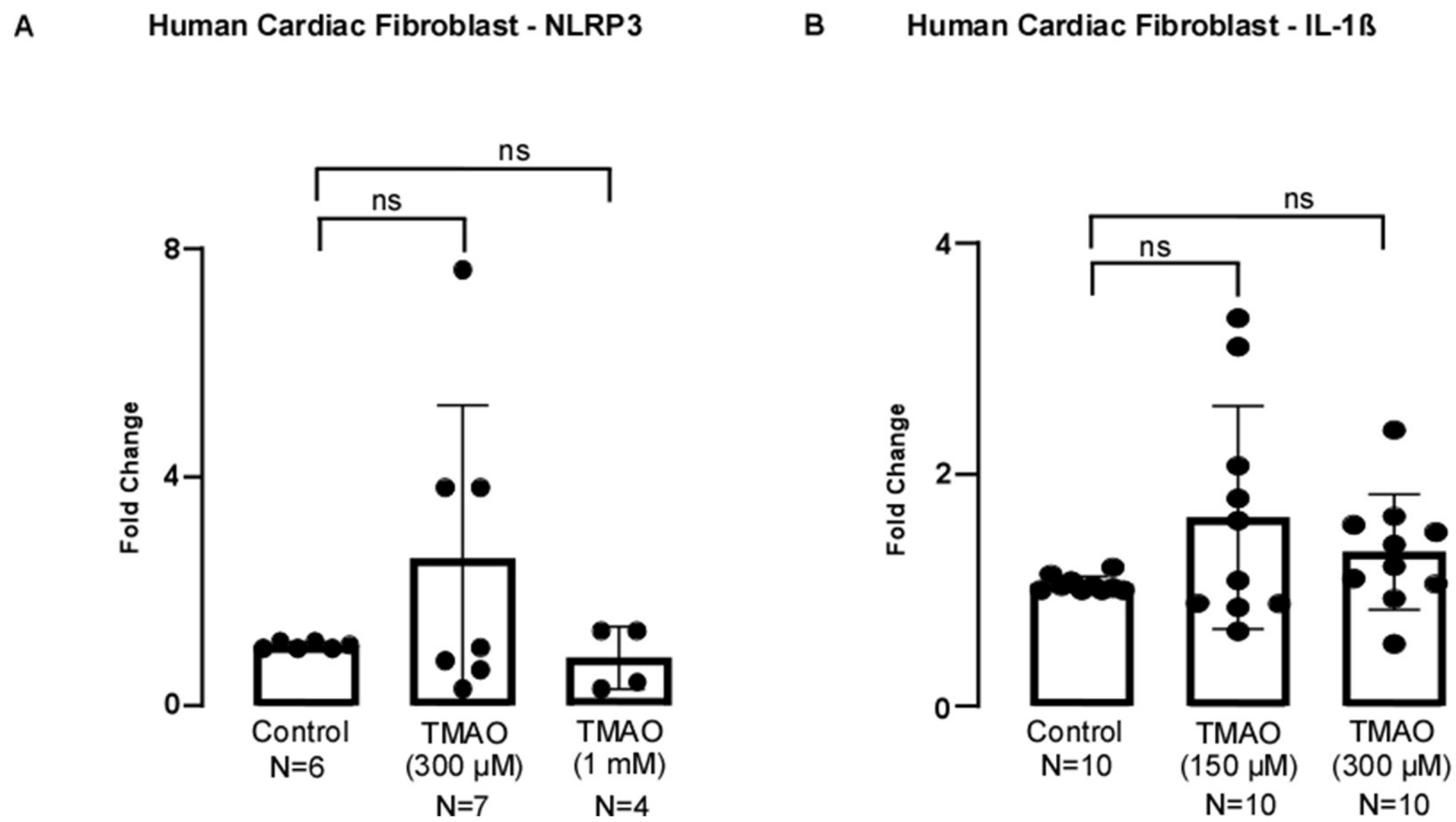

Supplemental Figure 26: (**A**) qPCR of NLRP3 (**B**) IL-β mRNA levels from the human cardiac fibroblast treated with control (water) or TMAO at the concentrations of 150 μM, 300 μM and 1 mM for 24h, presented as fold expression to average mRNA levels present in cells treated using GAPDH as a housekeeping control group. Each box plot represents mean ±SD. A Kruskal-Wallis test with Dunn's post hoc analysis was used to determine the significant difference between the medians of three independent diet groups and multiple paired comparison adjustments were performed. ns, non-significant.

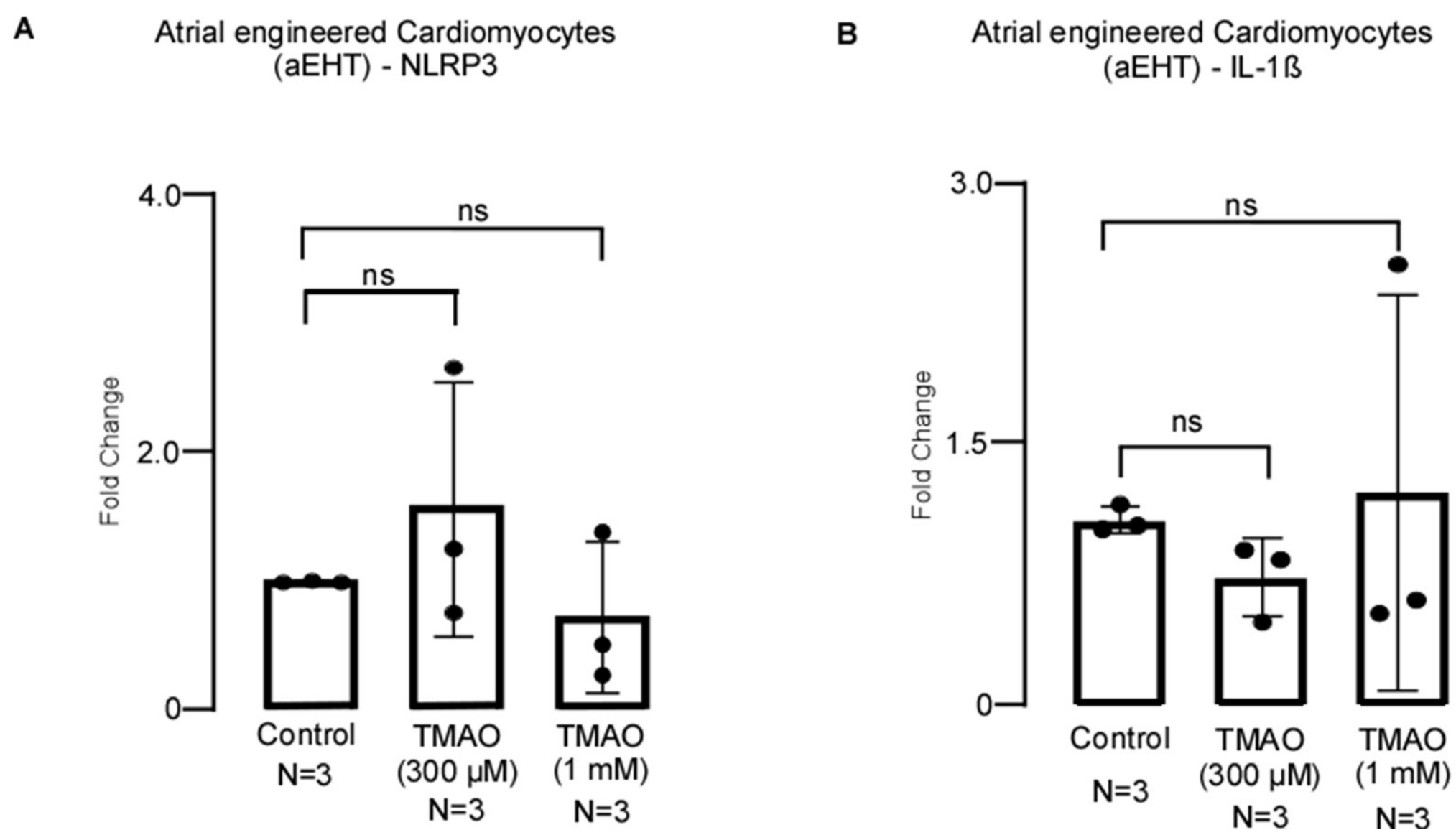

Supplemental Figure 27: (**A**) qPCR of NLRP3 and (**B**) IL-β mRNA levels from atrial engineered cardiomyocytes treated with control (water) or TMAO at the concentrations of 300 µM and 1 mM for 24h, presented as fold expression to average mRNA levels present in cells treated with GAPDH as a housekeeping control group. Each box plot represents mean ±SD. A Kruskal-Wallis test with Dunn's post hoc analysis was used to determine the significant difference between the medians of three independent diet groups and multiple paired comparison adjustments were performedns, non-significant.

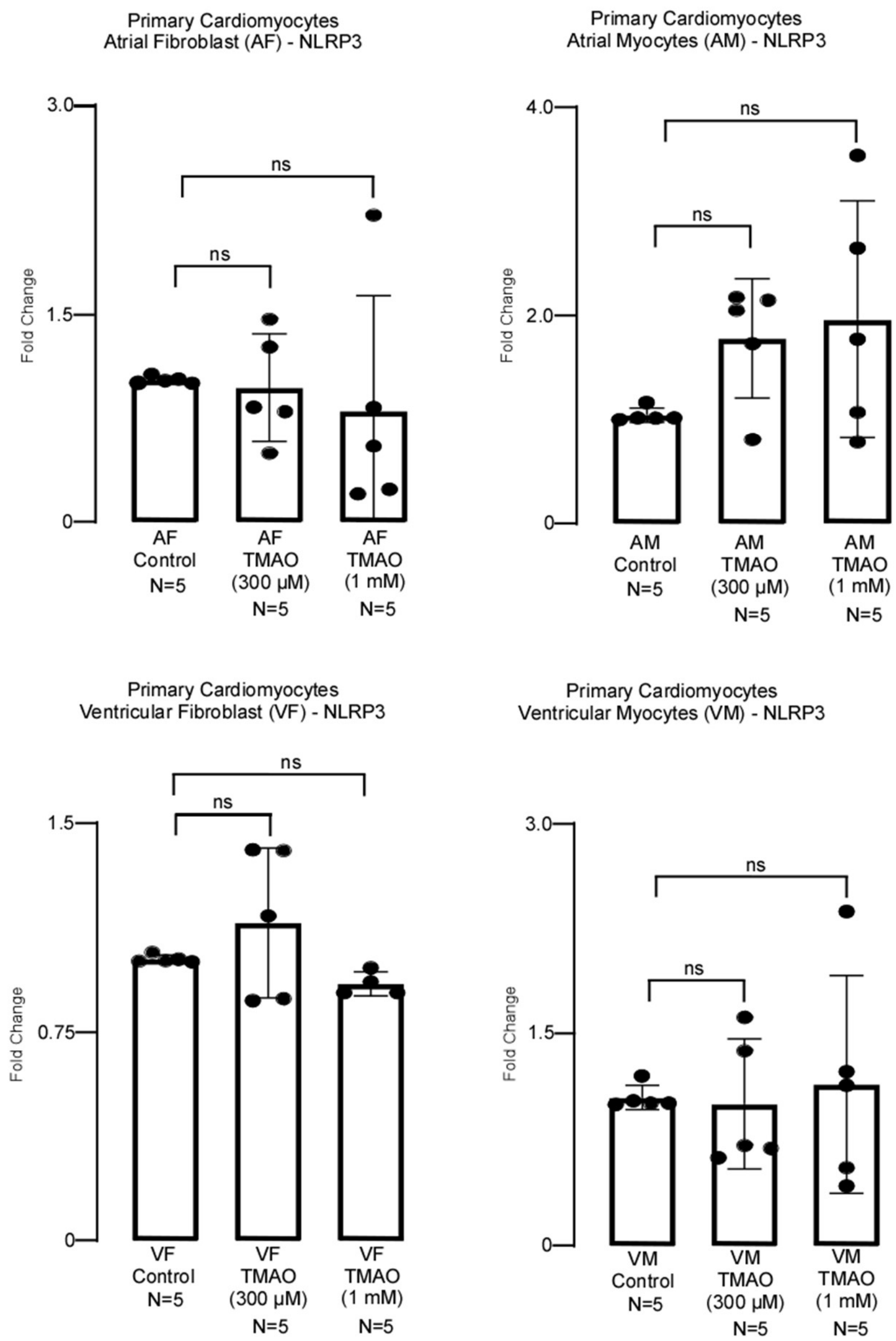

Supplemental Figure 28: qPCR of NLRP3 mRNA levels from the primary atrial and ventricular fibroblast and primary atrial and ventricular myocytes of CREM-IbΔC-X mice treated with control (water) or TMAO at the concentrations of 300 µM and 1 mM for 24h, presented as fold expression to average mRNA levels present in cells treated with GAPDH as a housekeeping control groups. Each box plot represents mean ±SD. A Kruskal-Wallis test with Dunn's post hoc analysis was used to determine the significant difference between the medians of three independent diet groups and multiple paired comparison adjustments were performed. ns, non-significant.

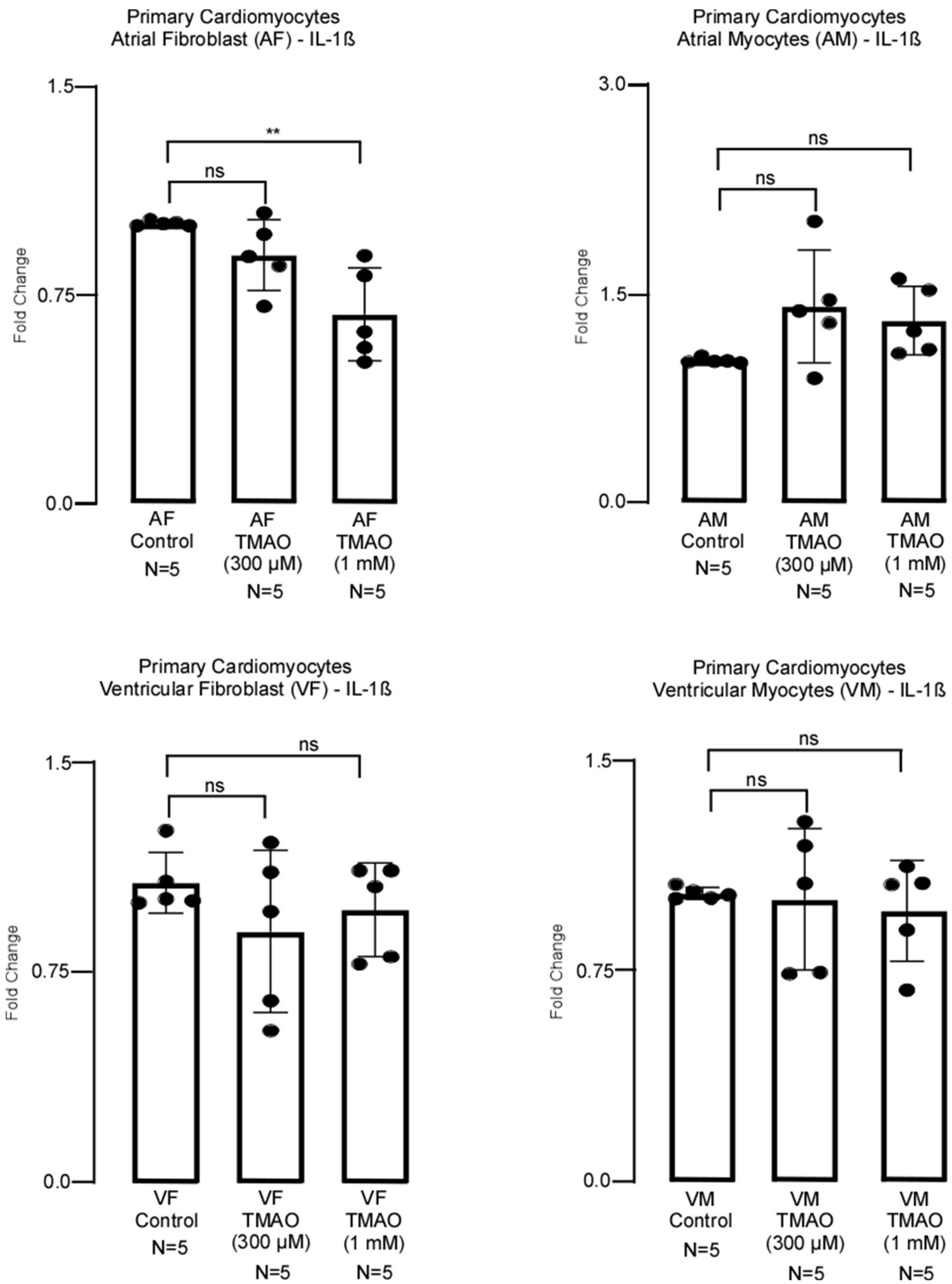

Supplemental Figure 29: qPCR of IL-β mRNA levels from the primary atrial and ventricular fibroblast and primary atrial and ventricular myocytes of CREM-IbΔC-X mice treated with control (water) or TMAO at the concentrations of 300 μM and 1 mM for 24h, presented as fold expression to average mRNA levels present in cells treated with GAPDH, as a housekeeping control groups. Each box plot represents mean ±SD. A Kruskal-Wallis test with Dunn's post hoc analysis was used to determine the significant difference between the medians of three independent diet groups and multiple paired comparison adjustments were performed. *$**P < 0.01$,* ns, non-significant.

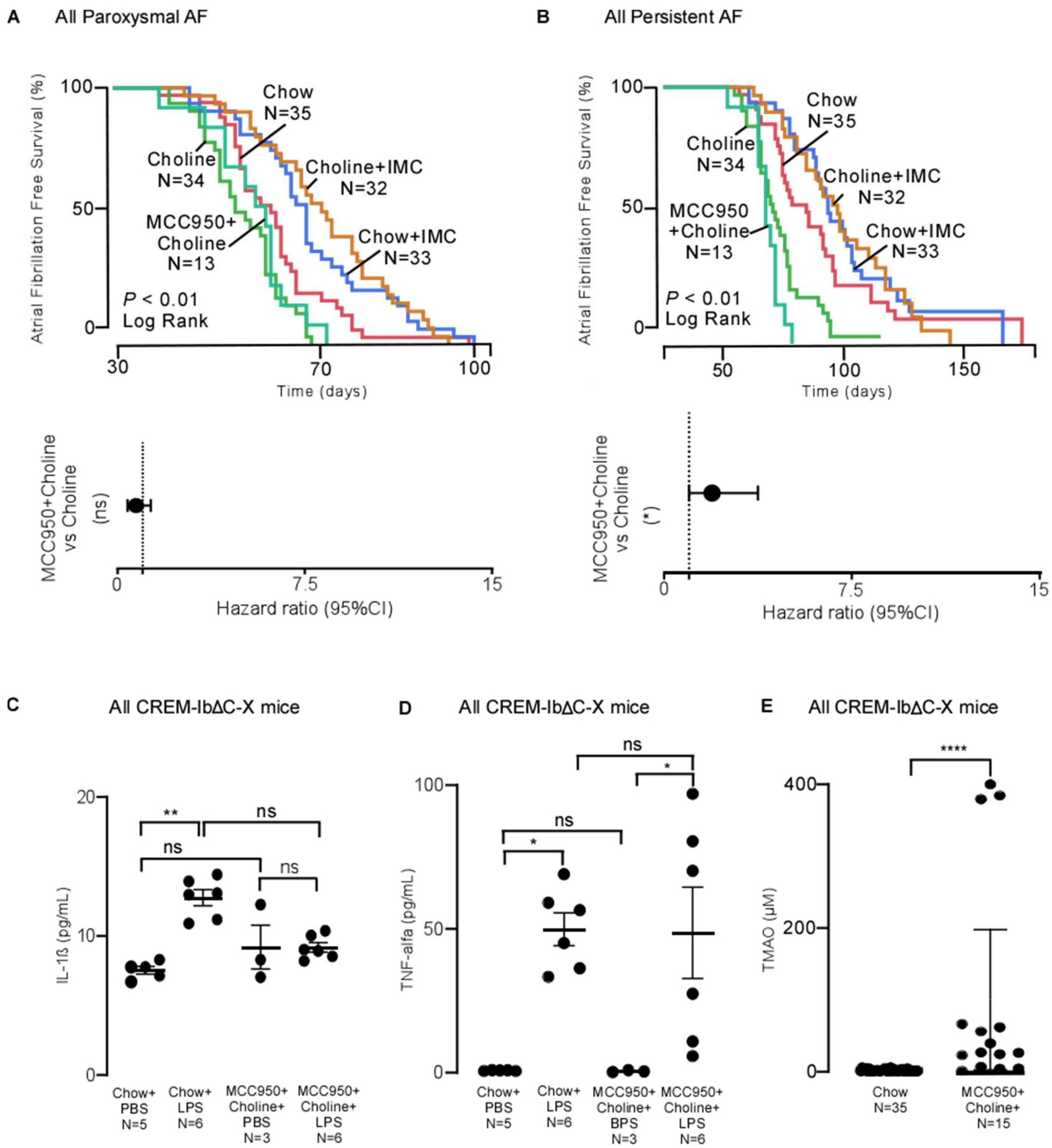

Supplemental Figure 30: Kaplan Meier plots and hazard ratios of the incidence of paroxysmal AF and persistent AF in CREM-IbΔC-X mice on respective diets. The incidence of paroxysmal AF (**A**) and persistent AF (**B**) in all CREM-IbΔC-X mice represented by a Kaplan Meier Plot (top panel) on the following diets: chow diet, choline supplemented diet, a chow + IMC supplemented diet, a choline + IMC supplemented diet, and MCC950 + choline supplemented diet. AF, atrial fibrillation; IMC, idomethylcholine; CI, confidence interval. In (**A,B**) (bottom panels), the circles represent the Hazard ratios (HRs) and the horizontal bars extend from the lower limit to the upper limit of the 95% CI of the estimate of the HR. HR between respective dietary groups were calculated using a Cox proportional hazard model. **P <.05, **P < .01*. Plasma concentration of (**C**)IL-1β and (**D**)TNF-α in all CREM-IbΔC-X mice on respective diets with or without MCC950. A Kruskal-Wallis test with Dunn's post hoc analysis was used to determine the significant difference between the medians of three independent diet groups and multiple paired comparison adjustments were performed. (**E**) Terminal plasma concentration of TMAO in in all CREM-IbΔC-X mice supplemented with chow diet and MCC950 + Choline diets. AF, atrial fibrillation; IMC, idomethylcholine. Each line denotes mean ±SD. A Mann Whitney test was used to assess significant differences between groups. **P <0.05, **P < 0.01, ***P < .00001*, ns, non-significant.

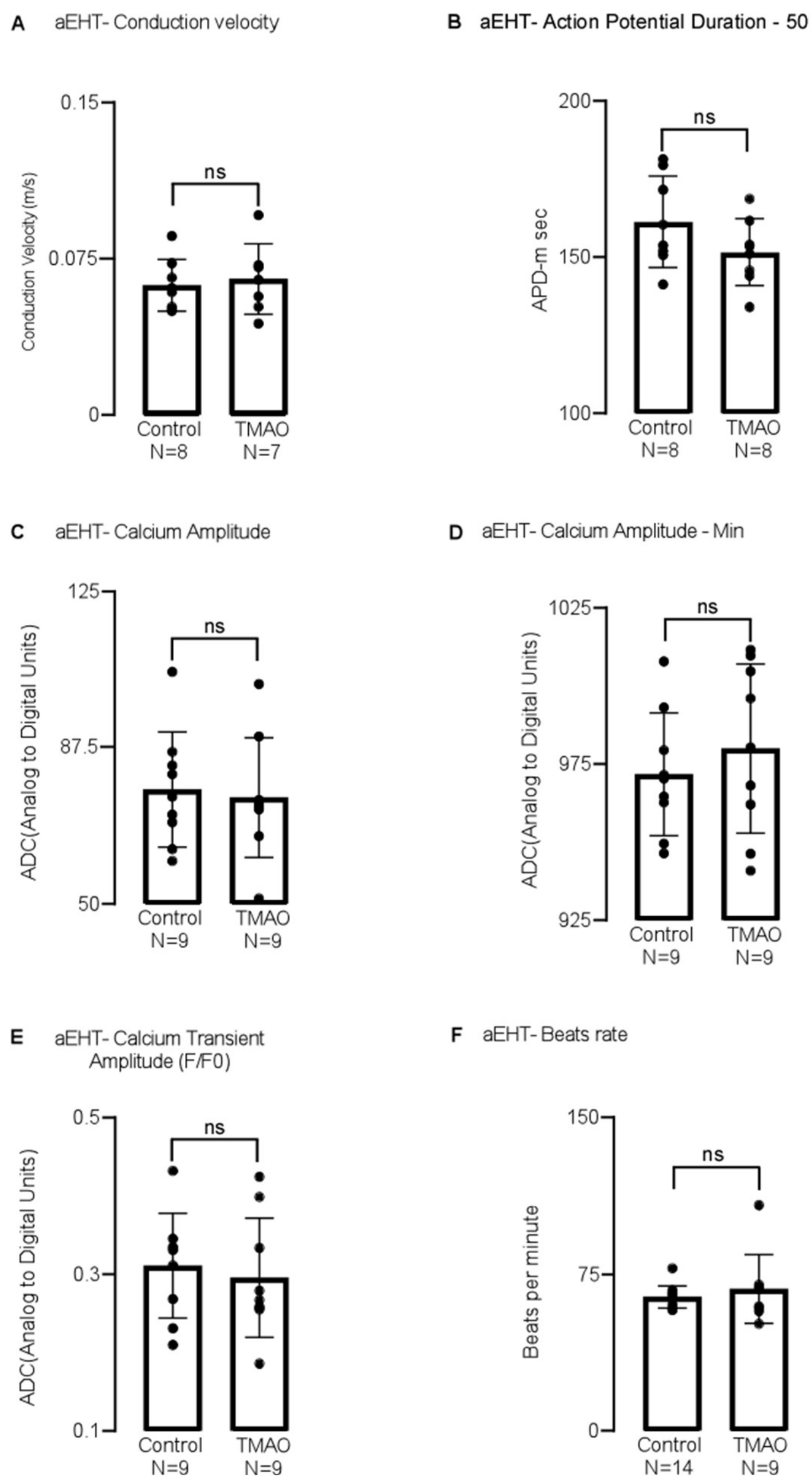

Supplemental Figure 31: Analysis of conduction velocity (CV) and action potential duration (APD) in artrial engineered cardiomycytes (aEHT) using optical mapping on cells exsposed to TMAO vs vehicle. Cell lines were acutely exposed with TMAO for 48 hours. Grouped data of (**A**) CV (**B**) APD 50, (**C**) Calcium amplitude, (**D**) Calcium amplitude minimum, (**E**) Calcium transient amplitude (F/F0) and (**F**) Beats rate. Each box plot represents mean ±SD. All *P* values shown were determined using the Wilcoxon signed rank test to compare the paired measurements before and after TMAO supplementation. ns, non-significant.

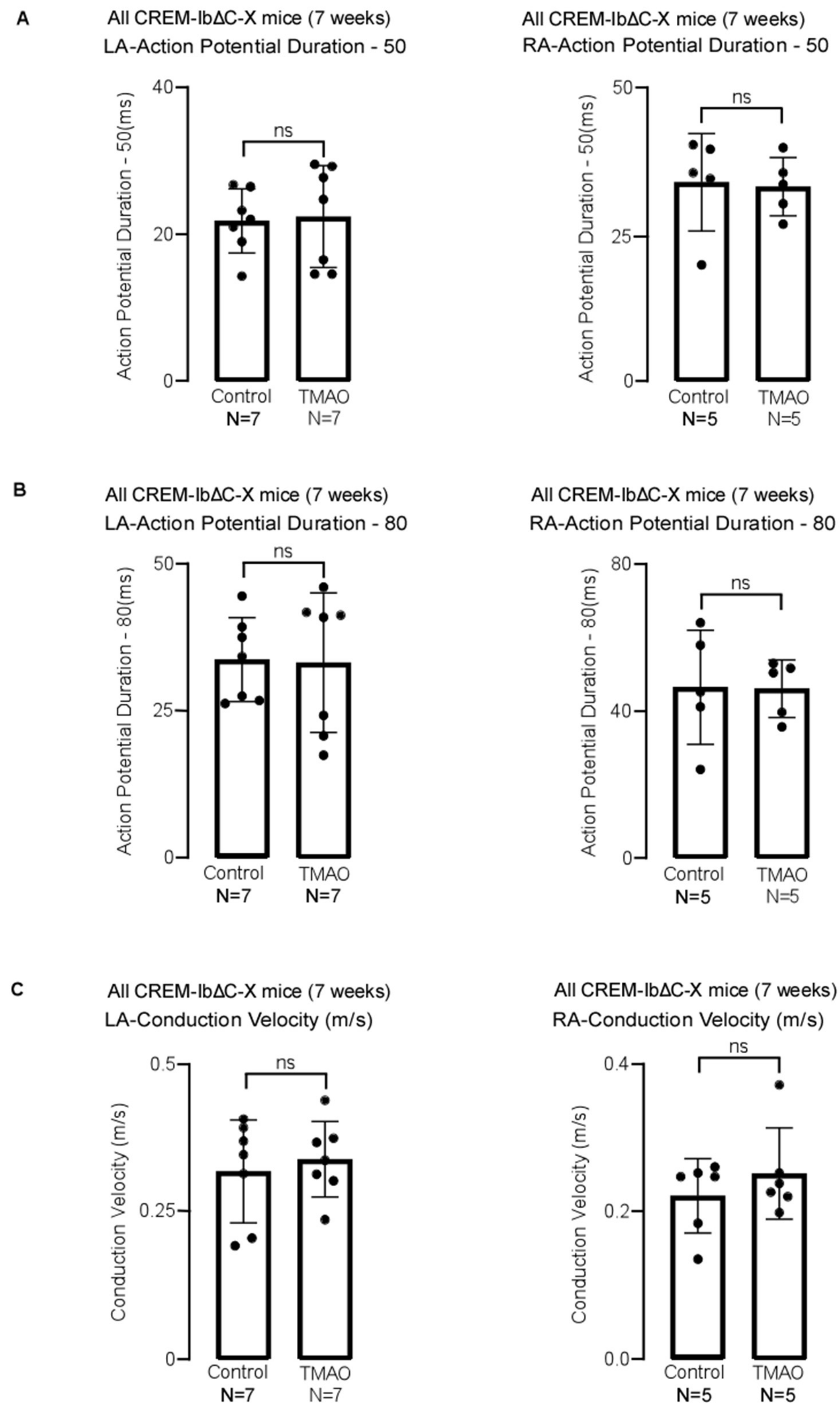

Supplemental Figure 32: Analysis of action potential duration (APD) and conduction velocity (CV) in male and female CREM-IbΔC-X *ex vivo* hearts when acutely exposed to TMAO for 40 min Vs vehicle. Grouped data of (**A**) APD 50 (**B**) APD 80 (**C**) Conduction velocity in left and right atria of all CREM-IbΔC-X mice. Each box plot represents mean ±SD. All *P* values shown were determined using the Wilcoxon signed rank test to compare the paired measurements before and after TMAO supplementation. ns, non-significant.

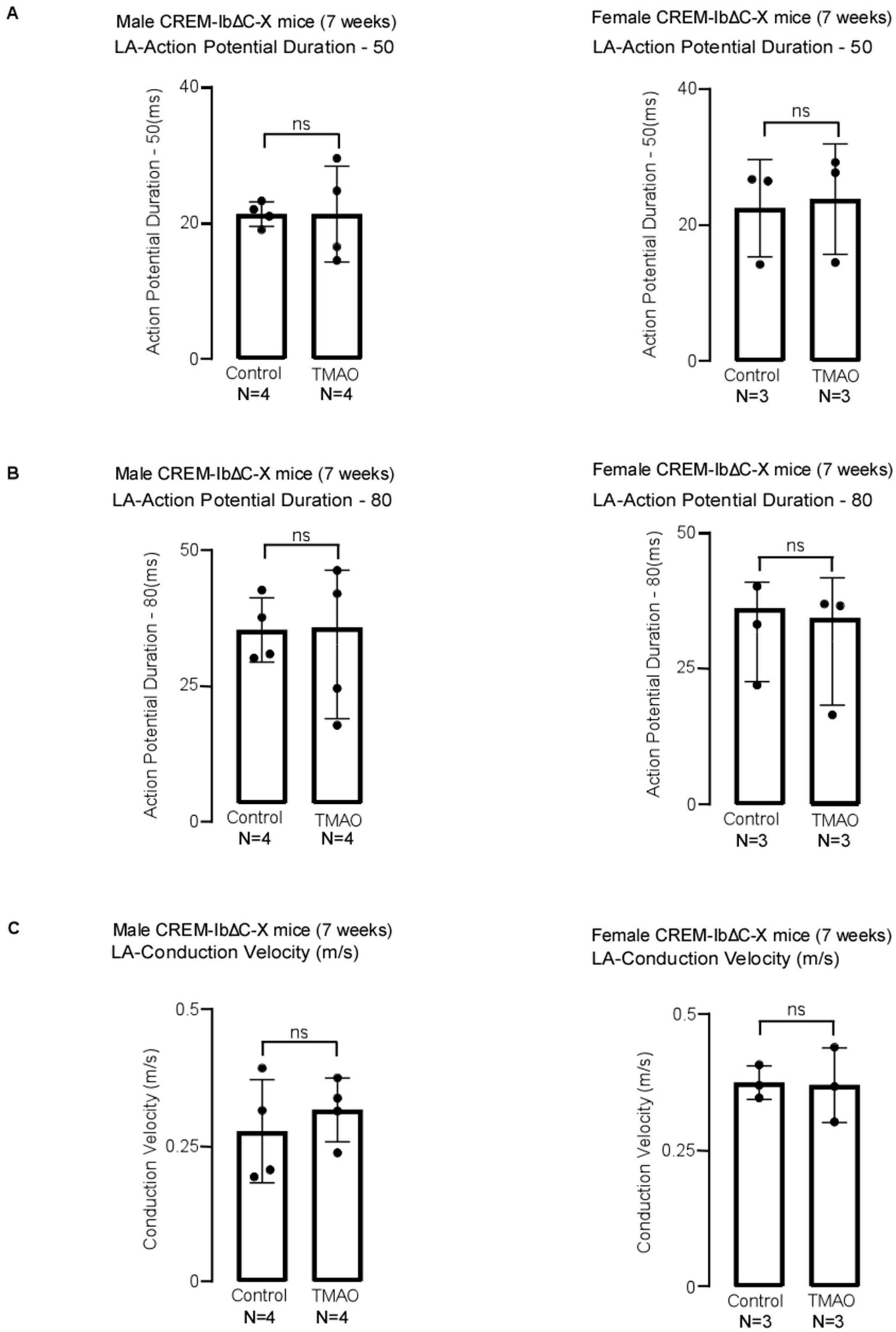

Supplemental Figure 33: Analysis of action potential duration (APD) and Conduction velocity (CV) of left atria in male and female CREM-IbΔC-X mice individually at 7 weeks of age on TMAO vs Control. Grouped data of (**A**) APD 50 (**B**) APD 80 (**C**) Conduction velocity in male only and female only mice. Each box plot represents mean ±SD. All *P* values shown were determined using the Wilcoxon signed rank test to compare the paired measurements before and after TMAO supplementation. ns, non-significant.

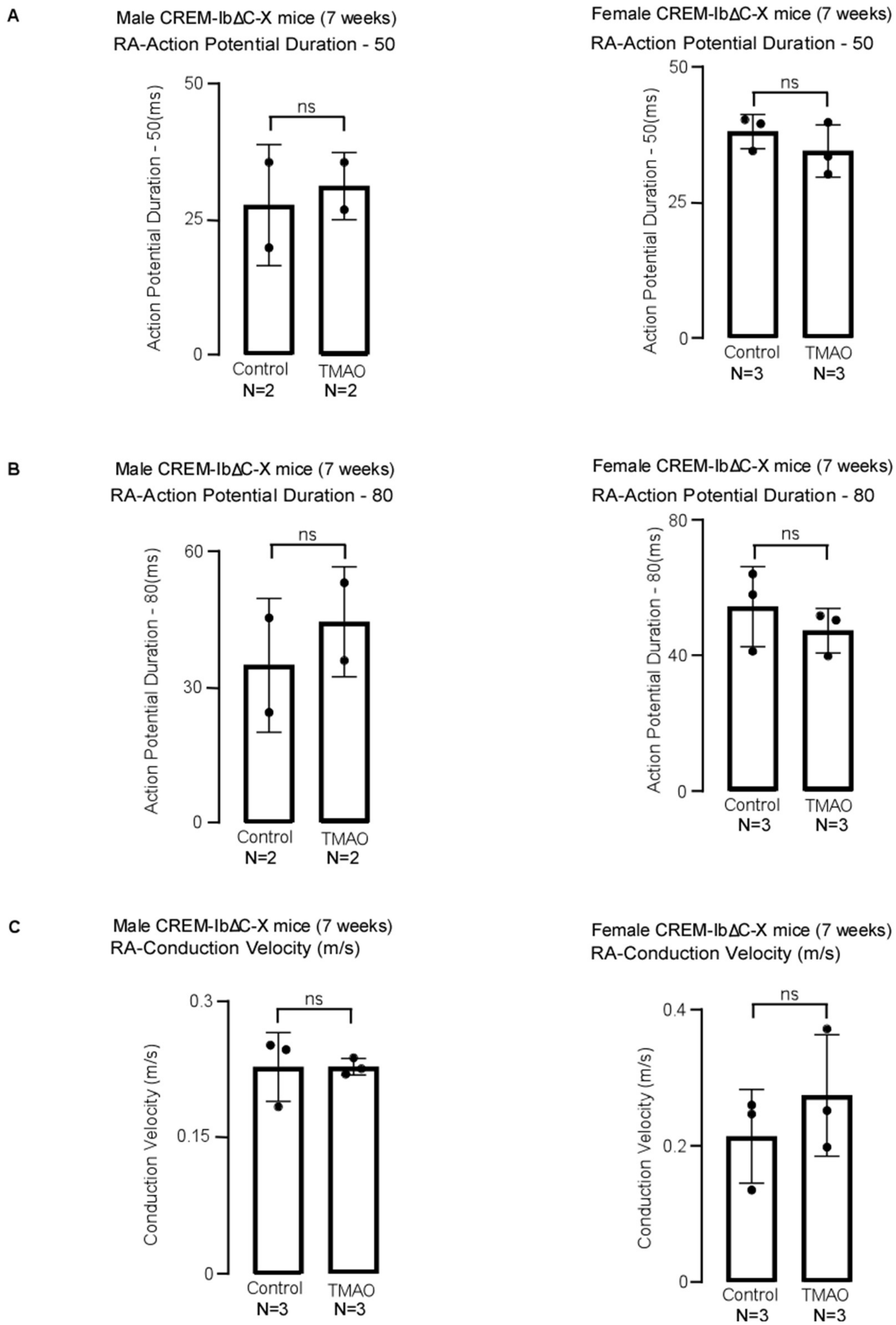

Supplemental Figure 34: Analysis of action potential duration (APD) and conduction velocity (CV) of right atria in male and female CREM-IbΔC-X mice individually at 7 weeks of age on TMAO vs Control. Grouped data of (**A**) APD 50 (**B**) APD 80 (**C**) Conduction velocity in male only and female only mice. Each box plot represents mean ±SD. All *P* values shown were determined using the Wilcoxon signed rank test to compare the paired measurements before and after TMAO supplementation. ns, non-significant.

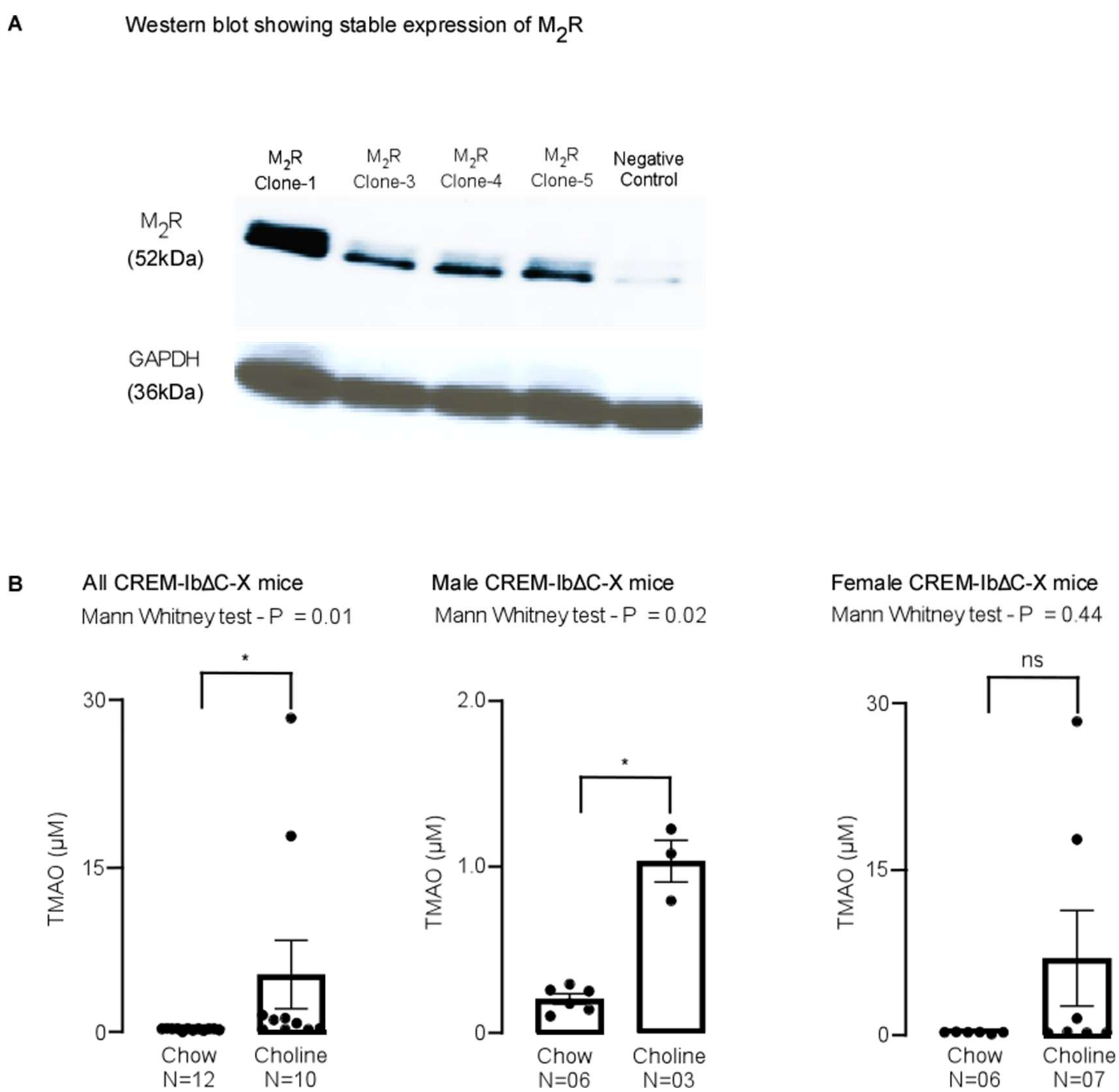

Supplemental Figure 35: (A) Western blot showing stable expression of M_2R (52 kDa) in HEK293 cell clones (Clone 1–4), with HEK293 cells alone used as the negative control. GAPDH (36 kDa) was used as the housekeeping and loading control. (**B**) Plasma concentrations of TMAO in all, both male and female CREM-IbΔC-X mice at 6 weeks of age fed on chow and choline supplemented diet used in the mouse HR experiments. Each box plot represents mean ± SEM. A Mann Whitney test was used to assess significant differences between groups. *$P < 0.05$*, ns, non-significant.

References

1. Wang Z, Klipfell E, Bennett BJ, Koeth R, Levison BS, Dugar B, et al. Gut flora metabolism of phosphatidylcholine promotes cardiovascular disease. *Nature.* 2011;472(7341):57-63.
2. Tchou G, Ponce-Balbuena D, Liu N, Gore-Panter S, Hsu J, Liu F, et al. Decreased FAM13B Expression Increases Atrial Fibrillation Susceptibility by Regulating Sodium Current and Calcium Handling. *JACC Basic Transl Sci.* 2023;8(10):1357-78.
3. Stumpel FT, Stein J, Himmler K, Scholz B, Seidl MD, Skryabin BV, et al. Homozygous CREM-IbDeltaC-X Overexpressing Mice Are a Reliable and Effective Disease Model for Atrial Fibrillation. *Front Pharmacol.* 2018;9:706.
4. Kirchhof P, Marijon E, Fabritz L, Li N, Wang W, Wang T, et al. Overexpression of cAMP-response element modulator causes abnormal growth and development of the atrial myocardium resulting in a substrate for sustained atrial fibrillation in mice. *Int J Cardiol.* 2013;166(2):366-74.
5. Oestereicher MA, Wotton JM, Ayabe S, Bou About G, Cheng TK, Choi JH, et al. Comprehensive ECG reference intervals in C57BL/6N substrains provide a generalizable guide for cardiac electrophysiology studies in mice. *Mamm Genome.* 2023;34(2):180-99.
6. Bukowska A, Felgendreher M, Scholz B, Wolke C, Schulte JS, Fehrmann E, et al. CREM-transgene mice: An animal model of atrial fibrillation and thrombogenesis. *Thromb Res.* 2018;163:172-9.
7. Bledzka KM, Manaserh IH, Grondolsky J, Pfleger J, Roy R, Gao E, et al. A peptide of the amino-terminus of GRK2 induces hypertrophy and yet elicits cardioprotection after pressure overload. *J Mol Cell Cardiol.* 2021;154:137-53.
8. Laurita KR, Khan S, McMahon T, Dennis AT, Li V, Gaivin R, et al. Ventricular arrhythmias in mouse models of diabetic kidney disease. *Sci Rep.* 2021;11(1):20570.
9. McPheeters MT, Wang YT, Werdich AA, Jenkins MW, and Laurita KR. An infrared optical pacing system for screening cardiac electrophysiology in human cardiomyocytes. *PLoS One.* 2017;12(8):e0183761.
10. Koeth RA, Wang Z, Levison BS, Buffa JA, Org E, Sheehy BT, et al. Intestinal microbiota metabolism of L-carnitine, a nutrient in red meat, promotes atherosclerosis. *Nat Med.* 2013;19(5):576-85.
11. Manaserh IH, Bledzka KM, Ampong I, Junker A, Grondolsky J, and Schumacher SM. A cardiac amino-terminal GRK2 peptide inhibits insulin resistance yet enhances maladaptive cardiovascular and brown adipose tissue remodeling in females during diet-induced obesity. *J Mol Cell Cardiol.* 2023;183:81-97.
12. Bolger AM, Lohse M, and Usadel B. Trimmomatic: a flexible trimmer for Illumina sequence data. *Bioinformatics.* 2014;30(15):2114-20.
13. Blanco-Miguez A, Beghini F, Cumbo F, McIver LJ, Thompson KN, Zolfo M, et al. Extending and improving metagenomic taxonomic profiling with uncharacterized species using MetaPhlAn 4. *Nat Biotechnol.* 2023;41(11):1633-44.
14. Li XS, Wang Z, Cajka T, Buffa JA, Nemet I, Hurd AG, et al. Untargeted metabolomics identifies trimethyllysine, a TMAO-producing nutrient precursor, as a predictor of incident cardiovascular disease risk. *JCI Insight.* 2018;3(6).
15. Roberts AB, Gu X, Buffa JA, Hurd AG, Wang Z, Zhu W, et al. Development of a gut microbe-targeted nonlethal therapeutic to inhibit thrombosis potential. *Nat Med.* 2018;24(9):1407-17.
16. Shih DM, Zhu W, Schugar RC, Meng Y, Jia X, Miikeda A, et al. Genetic Deficiency of Flavin-Containing Monooxygenase 3 (Fmo3) Protects Against Thrombosis but Has Only a Minor Effect on Plasma Lipid Levels-Brief Report. *Arterioscler Thromb Vasc Biol.* 2019;39(6):1045-54.
17. Mohan ML, Nagatomo Y, Saha PP, Mukherjee SD, Engelman T, Morales R, et al. The IgG3 subclass of beta1-adrenergic receptor autoantibodies is an endogenous biaser of beta1AR signaling. *Mol Biol Cell.* 2021;32(7):622-33.

18. Burridge PW, Holmstrom A, and Wu JC. Chemically Defined Culture and Cardiomyocyte Differentiation of Human Pluripotent Stem Cells. *Curr Protoc Hum Genet.* 2015;87:21 3 1- 3 15.
19. Devalla HD, Schwach V, Ford JW, Milnes JT, El-Haou S, Jackson C, et al. Atrial-like cardiomyocytes from human pluripotent stem cells are a robust preclinical model for assessing atrial-selective pharmacology. *EMBO Mol Med.* 2015;7(4):394-410.
20. Lemme M, Ulmer BM, Lemoine MD, Zech ATL, Flenner F, Ravens U, et al. Atrial-like Engineered Heart Tissue: An In Vitro Model of the Human Atrium. *Stem Cell Reports.* 2018;11(6):1378-90.
21. Breckwoldt K, Letuffe-Breniere D, Mannhardt I, Schulze T, Ulmer B, Werner T, et al. Differentiation of cardiomyocytes and generation of human engineered heart tissue. *Nat Protoc.* 2017;12(6):1177-97.
