# Supplementary material for: Gut microbial trimethylamine N-oxide generation promotes risk of atrial fibrillation via muscarinic receptor–mediated autonomic dysfunction
Source: J Clin Invest. 2026 Jul 24;136(17):e201684. doi: 10.1172/JCI201684 (PMC13528925; doi:10.1172/JCI201684)
Supplement: Supplementary file 2 [file jci-136-201684-s217.pdf]

Anti-HA

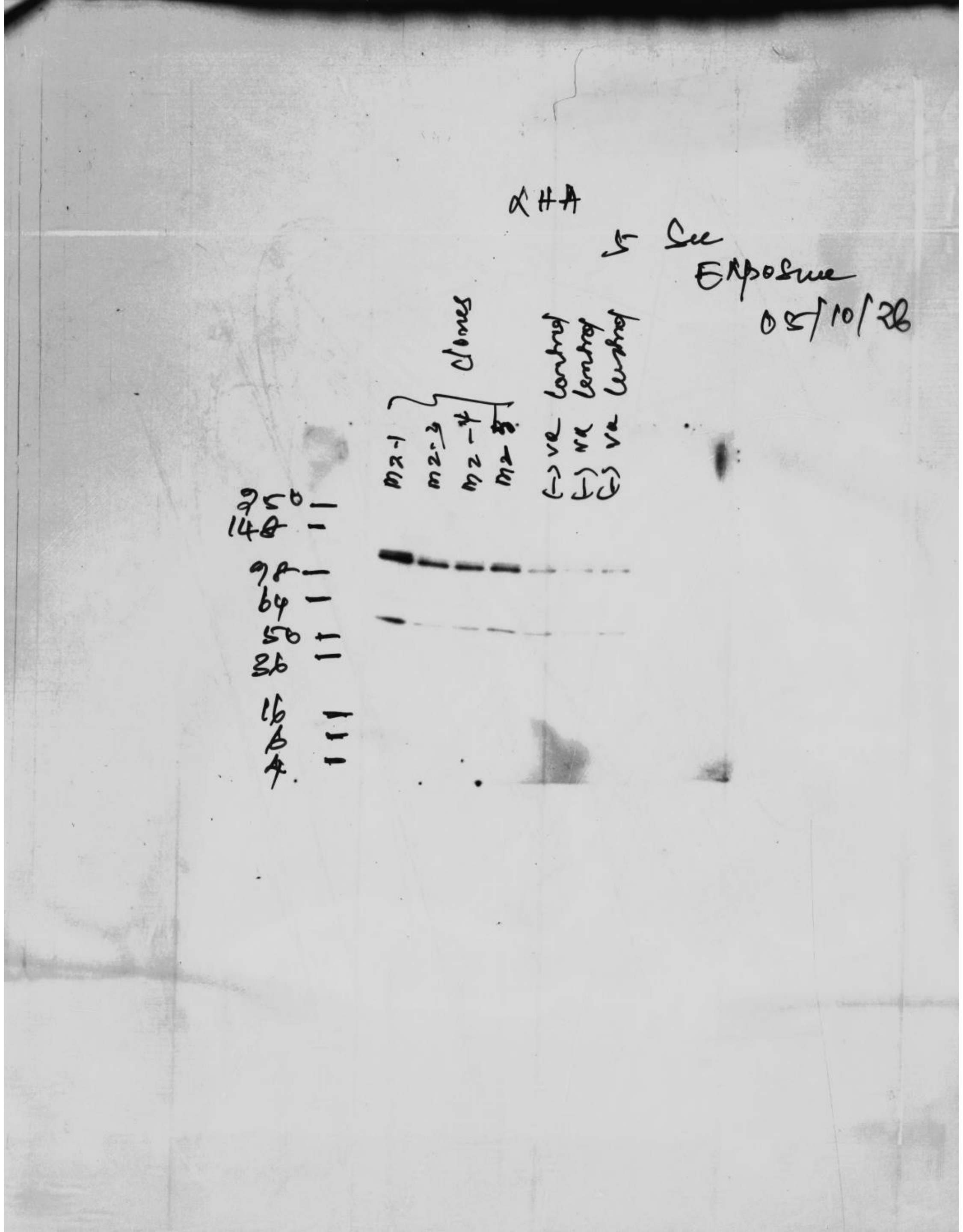
αHA
5 sec
Exposure
05/10/26
clones
mz-1
mz-2
mz-4
(-) ve control
(+) ve control
(-) ve control
250
148
98
64
50
36
16
6
4

Gapdh

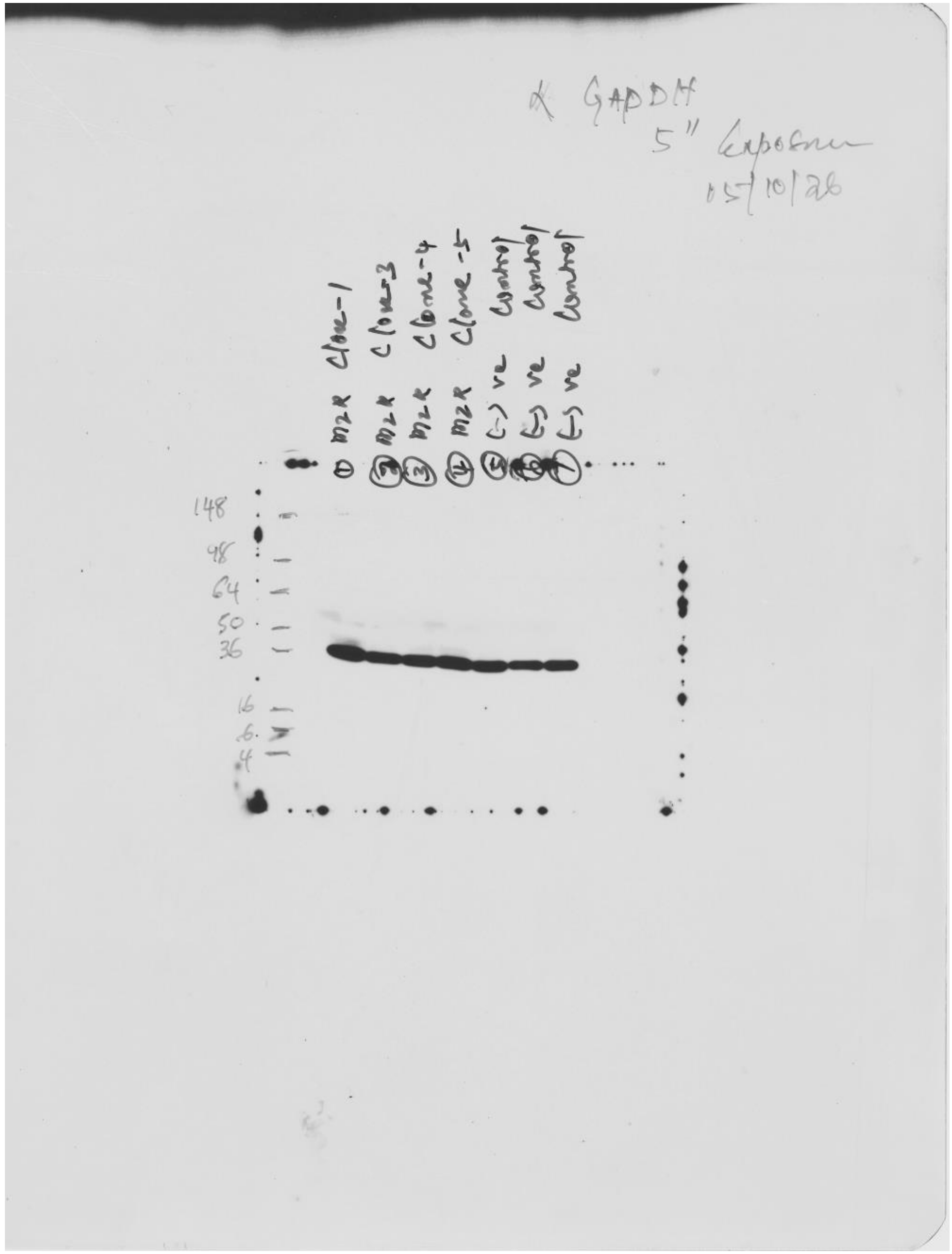
GAPDH
5"
M2R Clone-1
M2R Clone-3
M2R Clone-4
M2R Clone-5
(-) ve Control
(-) ve Control
(-) ve Control
148
98
64
50
36
16
6
4
